# Supplementary material for: High-quality, genome-wide SNP genotypic data for pedigreed germplasm of the diploid outbreeding species apple, peach, and sweet cherry through a common workflow
Source: PLoS One. 2019 Jun 27;14(6):e0210928. doi: 10.1371/journal.pone.0210928 (PMC6597046; doi:10.1371/journal.pone.0210928)

**High-quality, genome-wide SNP genotypic data for pedigreed germplasm of the diploid outbreeding species apple, peach, and sweet cherry through a common workflow**

Stijn Vanderzande, Nicholas P Howard, Lichun Cai, Cassia Da Silva Linge, Laima Antanaviciute, Marco CAM Bink, Johannes W Kruisselbrink, Nahla Bassil, Ksenija Gasic, Amy Iezzoni, Eric Van de Weg, Cameron Peace

**S1 Fig.** Call rates observed for individuals classified as having good, intermediate, or bad quality of genotypic data as defined by their B-allele frequency plot outcome. Higher call rates are observed for individuals with better quality of genotypic data.


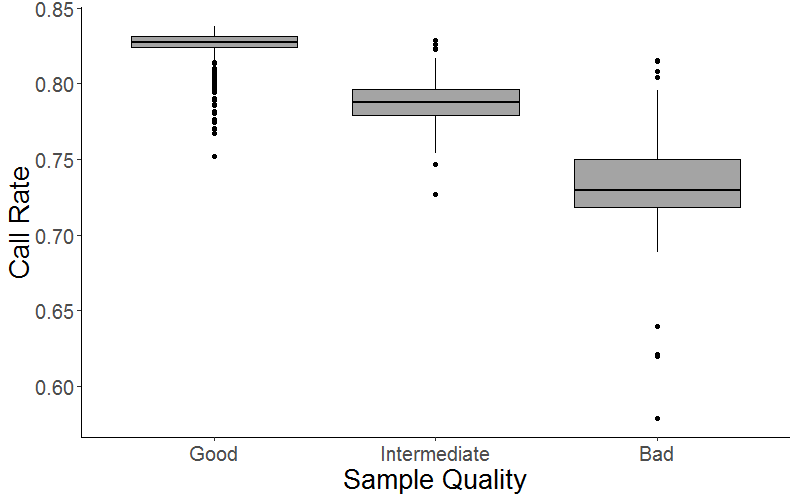

Supplement: S1 Fig — Higher call rates are observed for individuals with better quality of genotypic data. (DOCX) [file pone.0210928.s011.docx]
