# Supplementary material for: High-quality, genome-wide SNP genotypic data for pedigreed germplasm of the diploid outbreeding species apple, peach, and sweet cherry through a common workflow
Source: PLoS One. 2019 Jun 27;14(6):e0210928. doi: 10.1371/journal.pone.0210928 (PMC6597046; doi:10.1371/journal.pone.0210928)
Supplement: S1 File — (DOCX) [file pone.0210928.s013.docx]

**High-quality, genome-wide SNP genotypic data for pedigreed germplasm of the diploid outbreeding species apple, peach, and sweet cherry through a common workflow**

Stijn Vanderzande, Nicholas P Howard, Lichun Cai, Cassia Da Silva Linge, Laima Antanaviciute, Marco CAM Bink, Johannes W Kruisselbrink, Nahla Bassil, Ksenija Gasic, Amy Iezzoni, Eric Van de Weg, Cameron Peace

**S1 File. Detailed description of some procedures performed during the steps of the workflow**

*Initial genetic map for apple*

For apple, an integrated genetic map based on five full-sib families with ‘Honeycrisp’ as common parent [1] was used as a framework to help align additional SNPs on the 8K array. The relative order of SNPs in the map of Howard and co-workers (2017) [1] was adjusted to be consistent with the ‘Golden Delicious’ double haploid genome sequence v1.1 [2] whenever this did not result in false detection of double recombination for the original mapping populations. Then, SNPs that were included in the iGL map [3] but not included by Howard and co-workers (2017) [1] were aligned based on relative marker order between common markers of both maps and the ‘Golden Delicious’ double haploid genome sequence v1.1 [2]. In cases of conflict between the iGL map and the reference genome, only the iGL map was used as reference. Genetic positions of newly added SNPs were determined so that, in the new map, they had the same position relative to the position of flanking markers as these SNPs did in the iGL map. Finally, any remaining unmapped SNPs were positioned based solely on relative physical positions according to the ‘Golden Delicious’ double haploid genome sequence v1.1 [2]. When the genetic position in the iGL map was known for repositioned or newly added SNPs, their genetic position in the new map was determined so that they had the same position relative to the position of flanking markers as they did in the iGL map. When no genetic position in the iGL map was available, the genetic position was determined so that, in the new map, they had the same position relative to the position of flanking markers as they did in the physical genome.

*Sample sheet preparation*

The received sample sheet(s) was (were) adjusted in Microsoft Excel as follows before using it as input for GenomeStudio®:

- The sample sheet was saved as an ‘xls(x)’ file to avoid the loss of ‘SentrixBarcode’ information that occasionally occurs when saving it as a ‘.csv’ file.
- When individuals were separated over multiple iScan runs and sample sheets, the ‘[Data]’ sections of each sample sheet were combined into one.
- A copy of the ‘Sample_ID’ column in the ‘[Data]’ section was added and named ‘Sample_Original’.
- Sample names in the ‘Sample_ID’ were adjusted to remove any spaces or special characters (needed for some software) and avoid long names or names that could be interpreted as dates (or other special formats) by Excel.
- Duplicate and parental information was added to the ‘Replicate’, ‘Parent1’, and ‘Parent2’ columns considering the adjusted names in the ‘Sample_ID’ column.
- The resulting sample sheet was saved both as a ‘.xlsx’ file for future editing and as a ‘.csv’ file to serve as an input file for GenomeStudio®.

*B-allele frequency plots*

In GenomeStudio®, the histogram of the B-allele frequency was plotted for each individual by opening the ‘Histogram plot’ function of the ‘Full Data Table’, choosing the first individual in the ‘Columns’ section, and then choosing ‘B Allele Freq’ in the ‘Sub Columns’ section. The histogram for the ‘B-allele frequency’ could then be plotted for each individual by scrolling through the individuals in the ‘Columns’ section.

To create B-allele frequency plots according to Chagné and co-workers (2015) [4], a subset of SNPs was created by applying the filter parameters described in S4A Table in the ‘SNP Table’ of GenomeStudio®. Next, the ‘Full Data Table’ of GenomeStudio® was adjusted to only contain the B-allele frequency of each sample: in the ‘Column Chooser’ function of GenomeStudio®, ‘B Allele Freq’ was added to the ‘Displayed Subcolumns’ section while all other subcolumns were removed from this section. The resulting ‘Full Data Table’ was exported using the ‘export displayed data to a file’ function. The exported ‘Data Table’ was further adjusted to the following format: the first column contained the SNPs name, the second column contained the SNP’s cumulative position, and all subsequent columns contained the samples’ B-allele-frequencies.

Each SNP’s cumulative genomic position was determined as follows: the chromosome number corresponding to the SNP was multiplied by the power of ten which ensured that the outcome was larger than any possible position within any chromosome (e.g., if the largest physical position within any chromosome was 456,437 bp, all chromosome numbers were multiplied by 1,000,000 or 10^6^ as this is the first power of 10 that is larger than 456,437. Similarly, if the largest genetic position within any chromosome was 145 cM, each chromosome number was multiplied by 1000 or 10^3^). Then, the physical or genetic position within the chromosome was added to the adjusted chromosome number to obtain the cumulative genomic position of that SNP. The resulting file was then loaded into R [5].

An ad hoc R-script (S2 File) generated a pdf file that contained a plot for each individual where ‘B-allele frequency’ values were plotted for the subset of SNP markers that were ordered according to their cumulative position on a genetic linkage map or reference genome sequence.

*ASSIsT input files*

The ‘Final Report’ and ‘DNA Report’ were generated using the ‘Report Wizard’ under the ‘Reports’ option of the ‘Analysis’ section. The best ‘redo’ was chosen based on the ‘10^th^ Percentile GC score’ and excluded samples were removed from the report. For the ‘Final Report’, ‘GTScore’, ‘Theta’, and ‘R’ were added to the default ‘Displayed Fields’ and data was grouped ‘by SNP’. For the ‘DNA Report’, samples were exported by ‘Sample ID’. The pedigree input file was created in Excel by copying the ‘Sample_ID’, ‘Parent1’, and ‘Parent2’ columns from the ‘[Data]’ section of the sample sheet used to create the GenomeStudio® project, adjusting the column names to ‘//SampleID’, ‘Mother’, and ‘Father’, respectively, and saving the resulting file as a tab-delimited text file. The (optional) map was created in Excel by having the SNP Names as given by GenomeStudio® in the first column and their corresponding chromosome and position within the chromosome (either physical or genetic) as the second and third column, respectively. Column names were set to ‘//SNPid’, ‘Chromosome’, and ‘Position’ and the resulting file was saved as a tab-delimited text file

*Plink analysis*

Plink input files generated with were copied into the folder that contained the PLINK executable (plink.exe). Then, a ‘command window’ or ‘PowerShell window’ was opened in this folder and the ‘plink.exe --file [*filename]* –missing-genotype - --genome full’ or ‘\plink.exe --file [*filename]* –missing-genotype - --genome full’ command was given, respectively, where [filename] was the name of the PLINK input files used. The resulting ‘plink.genome’ was opened in Excel and the ‘PI_HAT’ column was used to represent the proportion of identity-by-descent (IBD) between each pair of individuals.

*R script for PC and PPC relationships*

To use the R script, the ‘.gtypes’ ASSIsT output file was further adjusted to the following format: the first column contained an individual’s ‘Sample ID’, the second and third columns contained the individual’s ‘Mother ID’ and ‘Father ID’, respectively, and the subsequent columns contained the individual’s genotypic data. Any missing parental information was set to ‘-’. All alleles found in the data set were defined in the ‘AlleleList’ parameter whereas characters used for missing genotypes or missing alleles were defined in the ‘MissGT’ and ‘MissAllele’ parameters respectively. After loading all functions defined in the R-script, the ‘CheckParAll()’ function was used to identify Mendelian-inconsistent errors for individuals with at least one known parent in the data set. To identify missing parents, the ‘FindPosParComb()’ function of the ad hoc R-script (S3 File) was used to find PC and PPC relationships. The maximum number of PC errors and PPC errors to still accept a PC relationship and PPC relationship, respectively, were set with the ‘thresholdPE’ and ‘thresholdPPE’ parameters of the ‘FindPosParComb()’ function, respectively.

*AB+AA-AA test*

The AB+AA-AA test to confirm grandparents-grandchild relationships counts the number of SNPs for which the grandchild has an ‘AB’ genotype and all the grandparents (or one parent and both the grandparents through the other parent) have an ‘AA’ genotype. This number should be zero as the ‘B’ allele present in the grandchild should be present in at least one of its grandparents. A similar count can be performed for the SNPs for which the grandchild is ‘AB’ and all grandparents have a ‘BB’ genotype. This count should also be zero.

*FlexQTL input files*

Three input files were needed to run FlexQTL DataPrepper: a map file, a pedigree file, and a data file. The map file was obtained by adjusting the ASSIsT map input file as follows: Column names were changed to ‘MarkerId’, Group’, and ‘Position’ and the file was saved as a comma-delimited file (.csv). The pedigree file was obtained by adjusting the ASSIsT pedigree input file as follows: column names were changed to ‘Name’, ‘Parent1’, and ‘Parent2’ and the file was saved in the ‘.csv’ format. The data file was obtained by converting the ‘FlexQTLDataPrepper’ from ASSIsT to the ‘.csv’ format. The data file (.dat) generated by FlexQTL DataPrepper was adjusted to ensure all individuals had either both parents specified or none. Any individual that had only one known parent was given a dummy parent. These dummy parents, as well as any named parent not in the data set, were added to the data input file with all their genotypic data set to missing.

*Manual SNP calling*

To correct calling within GenomeStudio®, individuals belonging to a single miscalled cluster were chosen using the ‘Lasso Mode’ of the ‘SNP Graph’. After ‘right-clicking’ on the ‘SNP Graph’, the ‘Define X Cluster Using Selected Samples’ was chosen where ‘X’ was the appropriate genotype cluster (‘AA’, ‘AB’, or ‘BB’). The few SNPs that could not have their genotype clusters assigned simultaneously in GenomeStudio® (e.g., because clusters were too closely positioned; one of the clusters for homozygous individuals was between x=0.4 and x=0.6, which is true for part of the paralogous SNP one of the homozygous clusters according to the ASSIsT Reference Manual p14 [6]; or because null alleles were present) were genotyped as follows. Individuals belonging to a single cluster were selected using the ‘Lasso Mode’ of the ‘SNP Graph’ in GenomeStudio®. ‘Sample_IDs’ of the chosen individuals were transferred to Excel by highlighting the ‘Sample_ID’ column in the ‘Sample Table’, using the ‘copy’ function of the ‘Samples Table’, and pasting them into Excel. In Excel, the copied ‘SampleIDs’ were then assigned a genotype call. This process was repeated until all individuals had their genotype assigned.

*Haploblock and haplotype determination*

For phasing, pedigree data was adjusted to ensure accurate phasing and haploblock border determination. parental information in the data input file of FlexQTL^TM^ was adjusted so that the pedigree was trimmed to remove intermediate progenitors without genotypic data unless they were represented by more than four direct offspring. Because Visual FlexQTL^TM^ does not consider any individual without offspring (e.g., new breeding selections) in haploblock determination, dummy offspring with missing genotypic data were added for individuals that did not have any offspring in the data set yet whose recombinations were desired to contribute to determination of haploblock borders. After phasing using FlexQTL^TM^, Visual FlexQTL^TM^ was used to define haploblock borders under ‘Tools>Export>Export haplotype blocks file’, creating the ‘HaploBlocks.map’ file that assigns each marker to a haploblock and could be used as input for PediHaplotyper.

For SNP phasing within haploblocks, the pedigree had to be trimmed as in haploblock determination to remove intermediate progenitors without genotypic data unless they were represented by more than four direct offspring. However, dummy offspring introduced for haploblock determination were removed again before phasing the data. FlexQTL^TM^ was then run again (parameter settings in S4D Table), with the resulting phasing found in the output file named ‘mhaplotypes.csv’, which was used as an input for PediHaplotyper.

The PediHaplotyper package [7] was loaded into R and the working directory was set to the location of the input files created above (‘HaploBlocks.map’, ‘mhaplotypes.csv’, ‘flexqtl.par’, and ‘flexqtl.sort’). In R, the function ‘fq_haplotyping_session(sessionID=’prefix", mapfile="HaploBlocks.map")’ was used to create the haplotype output files in the working directory where ‘prefix’ was user-defined text that prefixed all output file names. The ‘prefix_hballeleles.dat’ output file listed the composition of each haplotype of each haploblock and the ‘prefix_flexqtl.dat’,’prefix_flexqtl.map’, and ‘prefix_flexqtl.par’ output files were used as input files for FlexQTL^TM^ for further data curation of the haplotyped data sets.

*References*

1. Howard NP, van de Weg E, Bedford DS, Peace CP, Vanderzande S, Clark MD, et al. Elucidation of the ‘Honeycrisp’ pedigree through haplotype analysis with a multi-family integrated SNP linkage map and a large apple (*Malus*×*domestica*) pedigree-connected SNP data set. Hortic Res. 2017;4: 17003. doi:10.1038/hortres.2017.3

2. Daccord N, Celton J-M, Linsmith G, Becker C, Choisne N, Schijlen E, et al. High-quality *de novo* assembly of the apple genome and methylome dynamics of early fruit development. Nat Genet. 2017;49: 1099–1106. doi:10.1038/ng.3886

3. Di Pierro EA, Gianfranceschi L, Di Guardo M, Koehorst-van Putten HJ, Kruisselbrink JW, Longhi S, et al. A high-density, multi-parental SNP genetic map on apple validates a new mapping approach for outcrossing species. Hortic Res. 2016;3: 16057. doi:10.1038/hortres.2016.57

4. Chagné D, Kirk C, Whitworth C, Erasmuson S, Bicknell R, Sargent DJ, et al. Polyploid and aneuploid detection in apple using a single nucleotide polymorphism array. Tree Genet Genomes. 2015;11: 94. doi:10.1007/s11295-015-0920-8

5. R Core Team. R: A Language and Environment for Statistical Computing [Internet]. Vienna, Austria: R Foundation for Statistical Computing; 2018. Available: http://www.R-project.org/

6. Di Guardo M, Micheletti D, Bianco L, Koehorst-van Putten HJJ, Longhi S, Costa F, et al. ASSIsT: an automatic SNP scoring tool for in- and outbreeding species - Reference Manual. 2015.

7. Voorrips RE, Bink MCAM, Kruisselbrink JW, Koehorst-van Putten HJJ, van de Weg WE. PediHaplotyper: software for consistent assignment of marker haplotypes in pedigrees. Mol Breed. 2016;36. doi:10.1007/s11032-016-0539-y
