# Supplementary material for: High-quality, genome-wide SNP genotypic data for pedigreed germplasm of the diploid outbreeding species apple, peach, and sweet cherry through a common workflow
Source: PLoS One. 2019 Jun 27;14(6):e0210928. doi: 10.1371/journal.pone.0210928 (PMC6597046; doi:10.1371/journal.pone.0210928)
Supplement: S4 File — (PDF) [file pone.0210928.s016.pdf]

United States Department of Agriculture
National Institute of Food and Agriculture

High-quality, genome-wide SNP genotypic data for pedigreed germplasm of the diploid outbreeding species apple, peach, and sweet cherry through a common workflow

Stijn Vanderzande, Nicholas P Howard, Lichun Cai, Cassia Da Silva Linge, Laima Antanaviciute, Marco CAM Bink,Johannes W Kruisselbrink, Nahla Bassil, Ksenija Gasic, Amy Iezzoni, Eric Van de Weg, Cameron Peace

S4 File: Hands-on guideline on how to perform data curation using the steps described in this study

RosBREED
DISEASE RESISTANCE × HORTICULTURAL QUALITY → SUPERIOR CULTIVARS

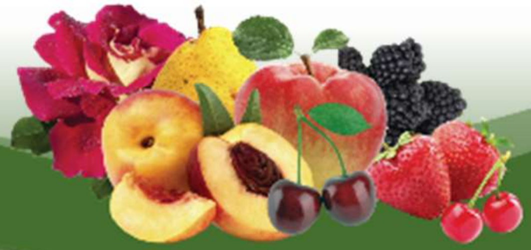

USDA

United States Department of Agriculture

National Institute of Food and Agriculture

Input data for Infinium arrays

RosBREED

DISEASE RESISTANCE × HORTICULTURAL QUALITY → SUPERIOR CULTIVARS

iSCAN data

Results for 1 array
(24 samples)

Sample sheet(s)
(.csv or .xls(x)format)

Name	Date modified	Type	Size
202017910001	9/26/2018 12:33 PM	File folder	
202017910002	9/26/2018 12:33 PM	File folder	
202017910004	9/26/2018 12:33 PM	File folder	
202017910005	9/26/2018 12:33 PM	File folder	
202017910007	9/26/2018 12:33 PM	File folder	
202017910014	9/26/2018 12:33 PM	File folder	
202017910021	9/26/2018 12:33 PM	File folder	
202017910025	9/26/2018 12:34 PM	File folder	
202017910036	9/26/2018 12:34 PM	File folder	
202017910037	9/26/2018 12:34 PM	File folder	
202017910042	9/26/2018 12:34 PM	File folder	
202017910045	9/26/2018 12:34 PM	File folder	
SweetCherry 062818.csv	7/20/2018 10:25 AM	Microsoft Excel C...	16 KB
SweetCherry 062818.xlsx	7/20/2018 10:21 AM	Microsoft Excel W...	29 KB

Manifest file received separately (.bpm format; describes probe content of the array)

RosBREED_Cherry_15k_15069617X343343_A1.bpm	7/20/2018 10:02 AM	BPM File	2,487 KB

iSCAN data

In result folder of an array

Results for 1 sample: rows 202017910001_R01C01_1_Focus_scan#1_swath#1_... through 202017910001_R01C01_Red.idat

202017910001.sdf	6/21/2018 10:10 AM	SDF File	32 KB
202017910001_qc.txt	6/28/2018 10:04 AM	TXT File	11 KB
202017910001_R01C01_1_Focus_scan#1_swath#1_...	6/28/2018 9:36 AM	JPEG image	214 KB
202017910001_R01C01_1_Green.xml	6/28/2018 9:37 AM	XML File	2 KB
202017910001_R01C01_1_Red.xml	6/28/2018 9:37 AM	XML File	2 KB
202017910001_R01C01_1-Swath1_Grn.jpg	6/28/2018 9:36 AM	JPEG image	2,100 KB
202017910001_R01C01_1-Swath1_Red.jpg	6/28/2018 9:36 AM	JPEG image	1,983 KB
202017910001_R01C01_Grn.idat	6/28/2018 9:37 AM	IDAT File	174 KB
202017910001_R01C01_Red.idat	6/28/2018 9:37 AM	IDAT File	174 KB
202017910001_R01C02_1_Focus_scan#1_swath#1_...	6/28/2018 9:39 AM	JPEG image	210 KB
202017910001_R01C02_1_Green.xml	6/28/2018 9:39 AM	XML File	2 KB
202017910001_R01C02_1_Red.xml	6/28/2018 9:39 AM	XML File	2 KB
202017910001_R01C02_1-Swath1_Grn.jpg	6/28/2018 9:39 AM	JPEG image	2,079 KB

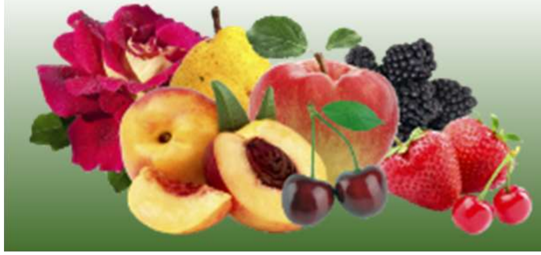

Sample sheet

Run info: rows 1–5 · Manifest info: rows 12–13 · Samples info: rows 16 onward

	A	B	C	D	E	F	G	H	I	J	K	L
1	[Header]											
2	Investigator Name											
3	Project Name											
4	Experiment Name											
5	Date											
5												
7	**NOTE**											
3	The following columns are required: Sample_ID , SentrixBarcode_A, SentrixPosition_A (and _B, _C etc.. if you have multiple manifests)											
9	All other columns are optional; any desired additional columns may be added here or "on the fly" during analysis											
0	Column order doesn't matter											
1												
2	[Manifests]											
3	A	RosBREED_Cherry_15k_15069617X343343_A1										
4												
5	[Data]											
6	Sample_ID	Samp	Sample_Well	SentrixBarcode_A	SentrixPo	Gender	Sample_G	Replicate	Parent1	Parent2	Source	
7	Rainier		A01	202017910001	R01C01		Chip1				Cherry	
8	Sandra_Rose		A02	202017910001	R02C01		Chip1				Cherry	
9	PC7144-11		A03	202017910001	R03C01		Chip1				Cherry	
:0	7147-9		A04	202017910001	R04C01		Chip1				Cherry	
:1	PC7309-4		A05	202017910001	R05C01		Chip1				Cherry	
:2	99F/132R4		A06	202017910001	R06C01		Chip1				Cherry	
:3	Ambrunes		A07	202017910001	R07C01		Chip1				Cherry	
:4	PC8011-6		A08	202017910001	R08C01		Chip1				Cherry	
:5	PC8012-5		A09	202017910001	R09C01		Chip1				Cherry	
[illegible]	[illegible]		[illegible]	[illegible]	[illegible]		[illegible]				[illegible]	

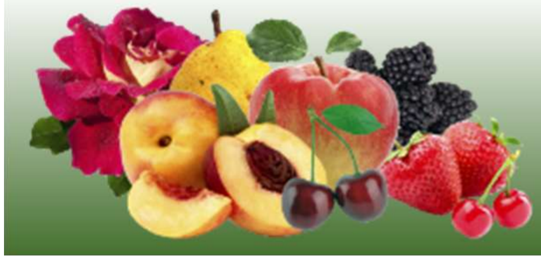

Sample sheet – sample data

[Data]										
Sample_ID	Samp	Sample_Well	SentrixBarcode_A	SentrixPosition_A	Gender	Sample_G	Replicate	Parent1	Parent2	Source
Rainier		A01	202017910001	R01C01		Chip1				Cherry
Sandra_Rose		A02	202017910001	R02C01		Chip1				Cherry
PC7144-11		A03	202017910001	R03C01		Chip1				Cherry
7147-9		A04	202017910001	R04C01		Chip1				Cherry
PC7309-4		A05	202017910001	R05C01		Chip1				Cherry
[illegible]		[illegible]	[illegible]	[illegible]		[illegible]				[illegible]

Sample’s name

Sample's original position in plate

Array number* (matches folder name)

Sample's position on array (matches filenames of sample)

Often not filled in!

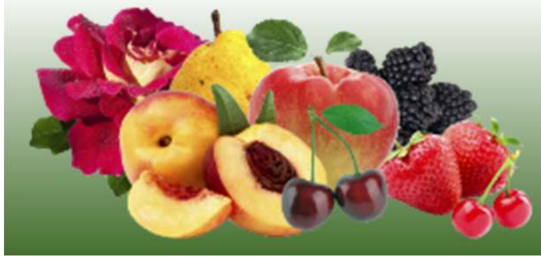

RosBREED

DISEASE RESISTANCE × HORTICULTURAL QUALI

SentrixBarcode_A	S
2.01382E+11	R
2.01382E+11	R
2.01382E+11	R
2.01382E+11	R
2.01382E+11	R
2.01382E+11	R
2.01382E+11	R
[illegible]	[illegible]

*Sometimes saved as ‘scientific number’ and last digits can get lost when saved as “.csv” file (make a “.xls(x)” copy!)

Sample sheet – sample data

- Sample_ID, SentrixBarcode_A and SentrixPosition_A are required, other columns are optional and many user-defined columns can be added, e.g.:
  - Alternative names
  - Tissue source
  - Subpopulation
  - Sample quality/ploidy (when known)

- Parent names must match names used under Sample_ID (if present in data set)

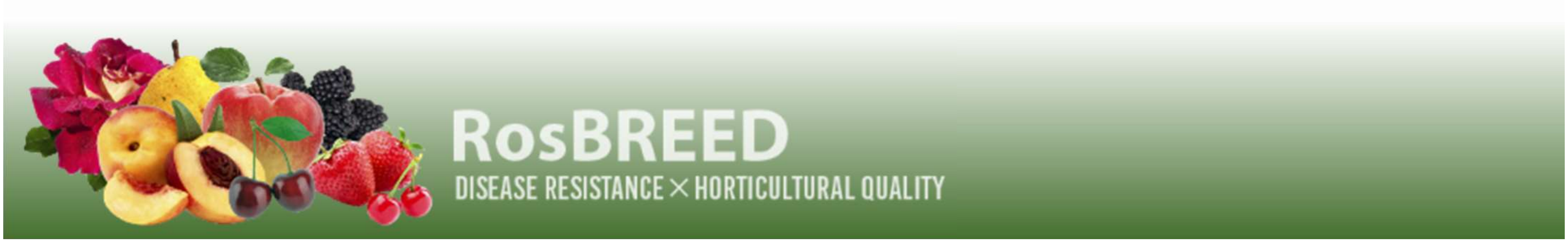

Sample sheet – sample_ID

- Check ‘Sample_ID’ of samples
  - Sometimes typing errors occurred
- Many software don’t accept spaces in names
  - Create new ‘Sample_ID’ column and save original Sample_ID as ‘Sample_ID_Original’
  - Remove spaces from new ‘Sample_ID’ column
  - Optional: Create abbreviated names for long names
- Parent and replicates names must match names used under new Sample_ID (if present in data set)

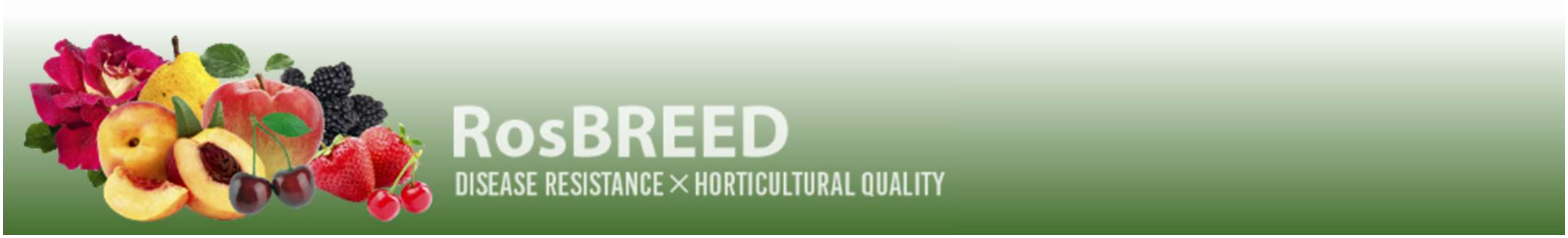

Sample sheet – sample data

16	Sample_ID	Alternative_Name	Sample_W	SentrixBarcode_	Sentrix	Gend	Sample	Replicate
353	Rainier		A01	202017910001	R01C01		Chip1	~~Rainier rep01~~
354	Rainier rep01		B08	201903100009	R08C01		Chip3	Rainier
355	Regina		B03	202017910007	R03C02		Chip5	
356	Salmo		B09	202017910036	R09C02		Chip 9	
357	Sandra Rose		G05	201903100003	R05C02		Chip2	~~Sandra Rose rep01~~
358	Sandra Rose rep01		A02	202017910001	R02C01		Chip1	Sandra Rose
359	Santina		H02	202017910025	R02C02		Chip8	~~Santina rep01~~
360	Santina rep01		H05	201903100003	R07C02		Chip2	Santina

Will lead to 2 versions of same report

- Replicates
  - Keep one original name
    - Easy to find in files generated in next steps
  - Add rep numbers for other replicates
    - Refer to original name as replicate

Combining sample sheets

- Move all array result folders into same folder (needed to load data)
  - GenomeStudio does not allow result folders to be spread across multiple subfolders
- Open first sample sheet and save first as **new** ".xls(x)" file (avoid loss of original sample sheet fila and loss of array number)
  - Make sure data columns match
  - Paste data rows below existing data,
    - Repeat until all data is included

United States Department of Agriculture

National Institute of Food and Agriculture

GenomeStudio®

RosBREED

DISEASE RESISTANCE × HORTICULTURAL QUALITY → SUPERIOR CULTIVARS

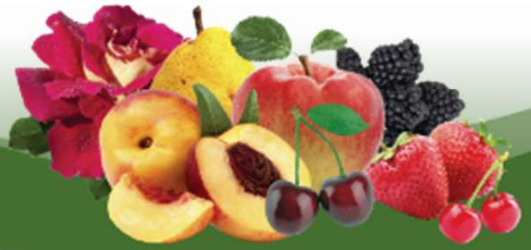

United States
Department of
Agriculture

National Institute
of Food and
Agriculture

Loading Data

RosBREED

DISEASE RESISTANCE × HORTICULTURAL QUALITY → SUPERIOR CULTIVARS

GenomeStudio

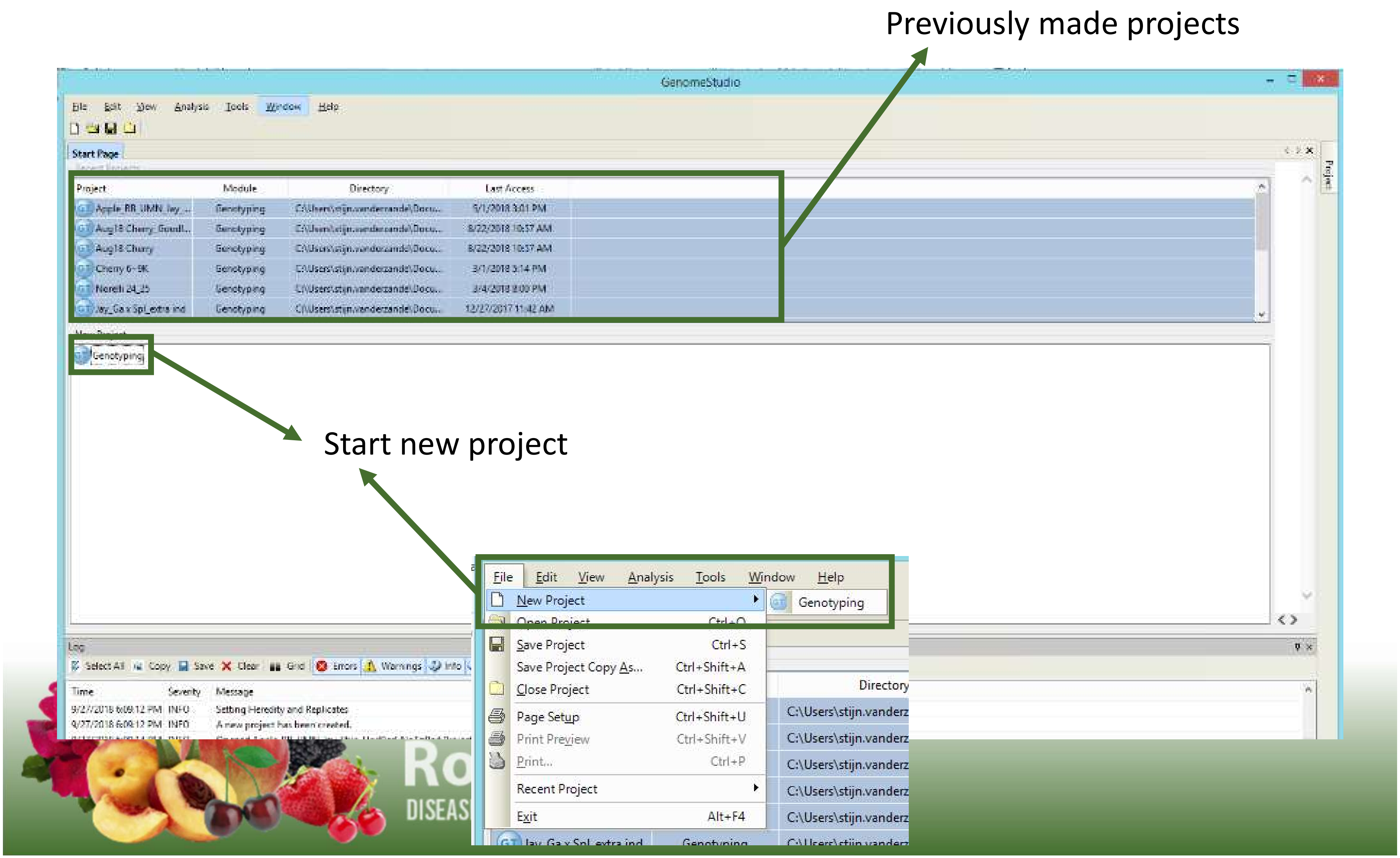

Loading data

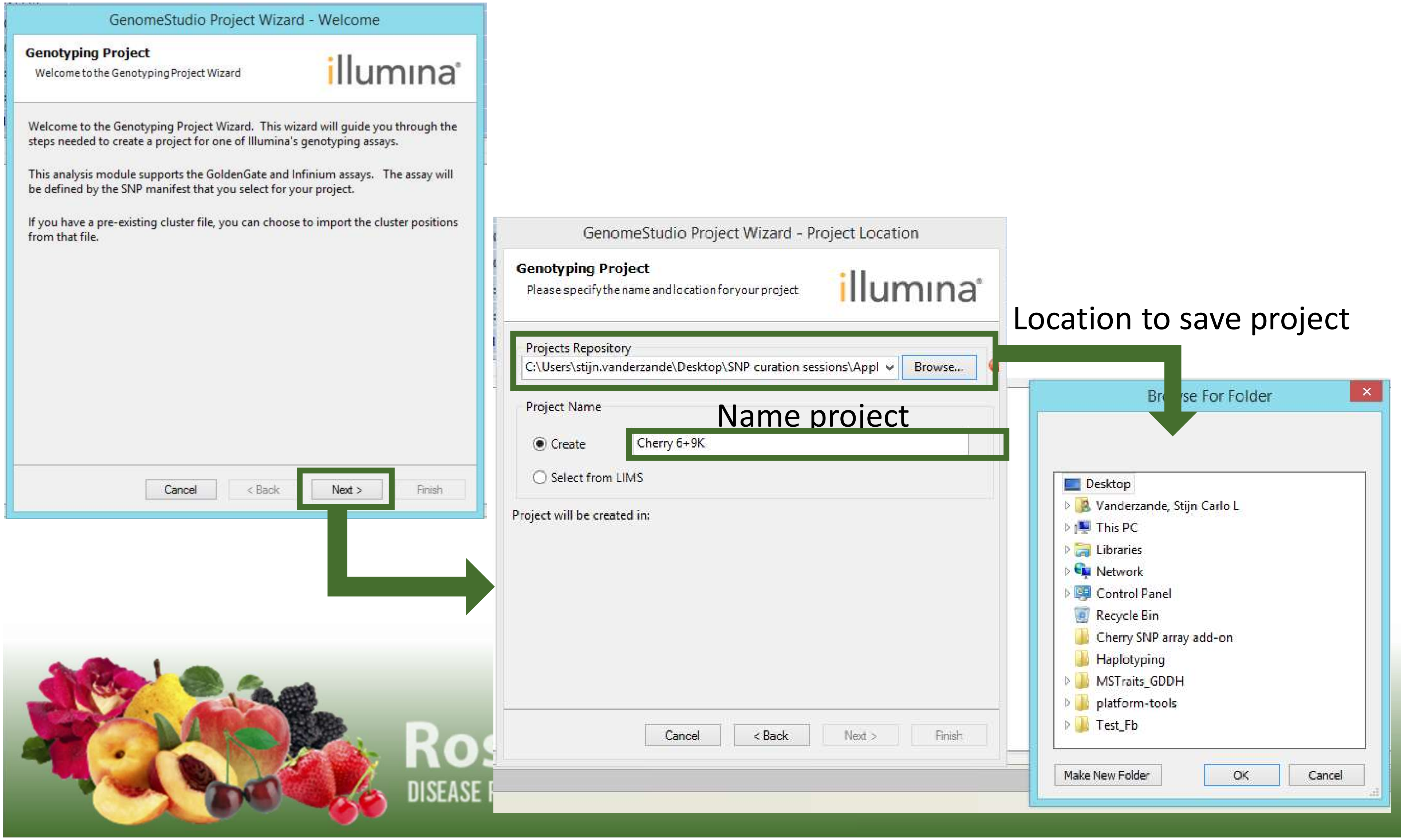

Loading data

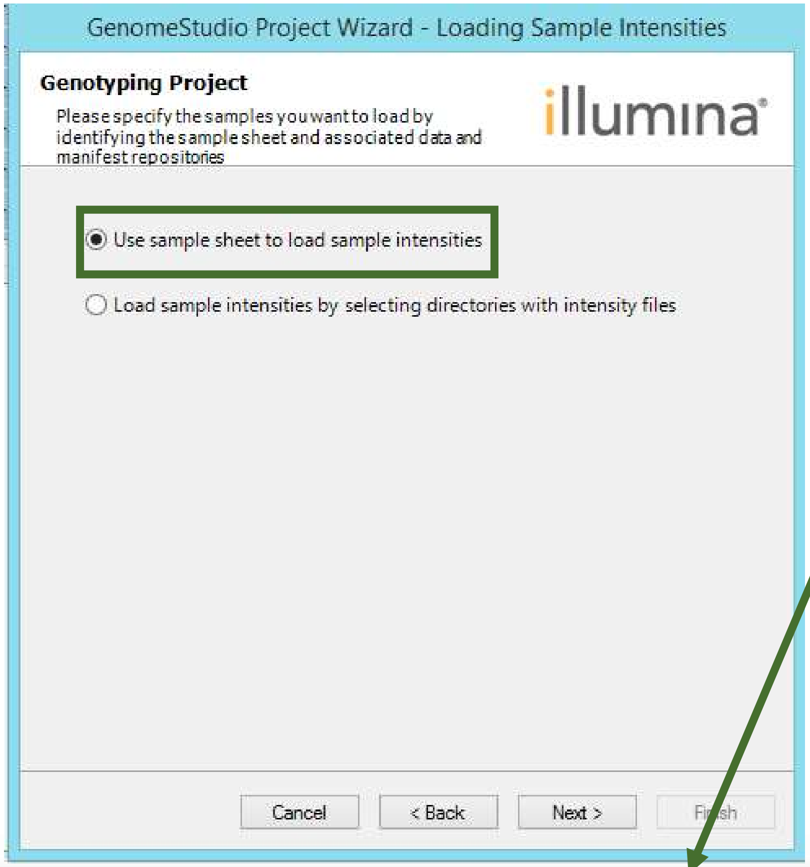

1 sample sheet file is loaded here

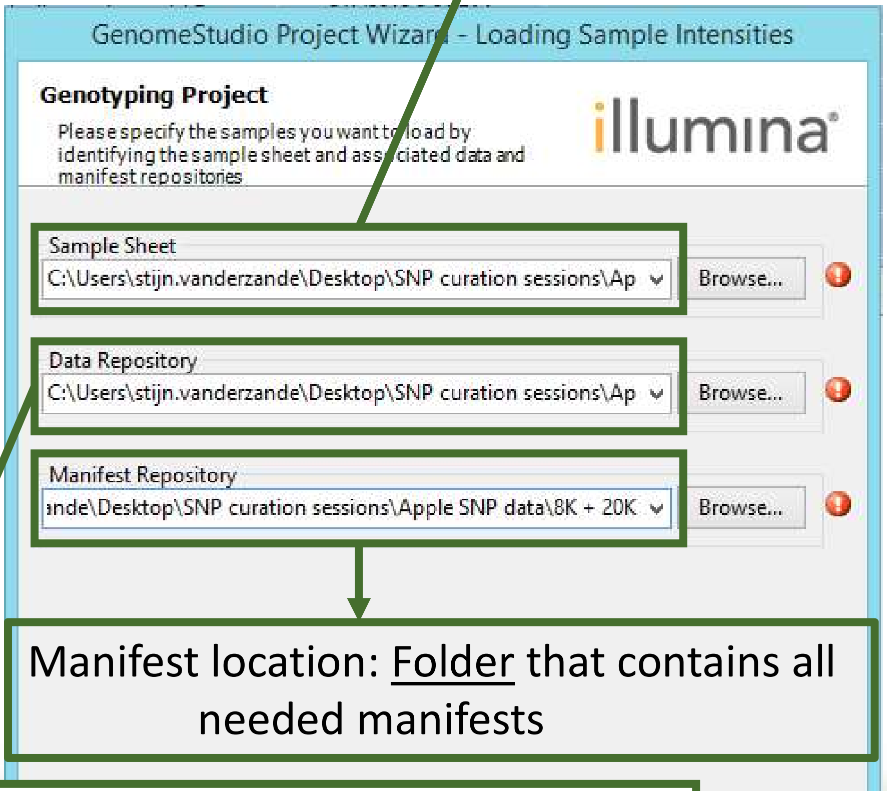

Manifest location: Folder that contains all needed manifests

Data location: Folder that contains all needed SNP array folders

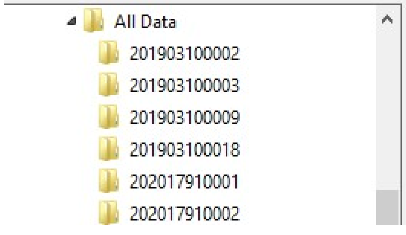

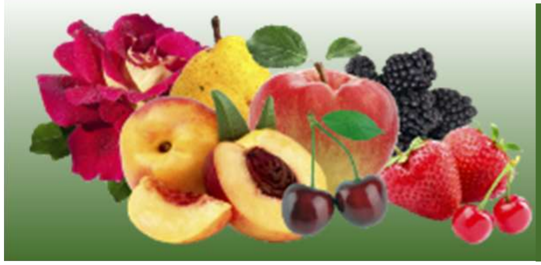

Loading data

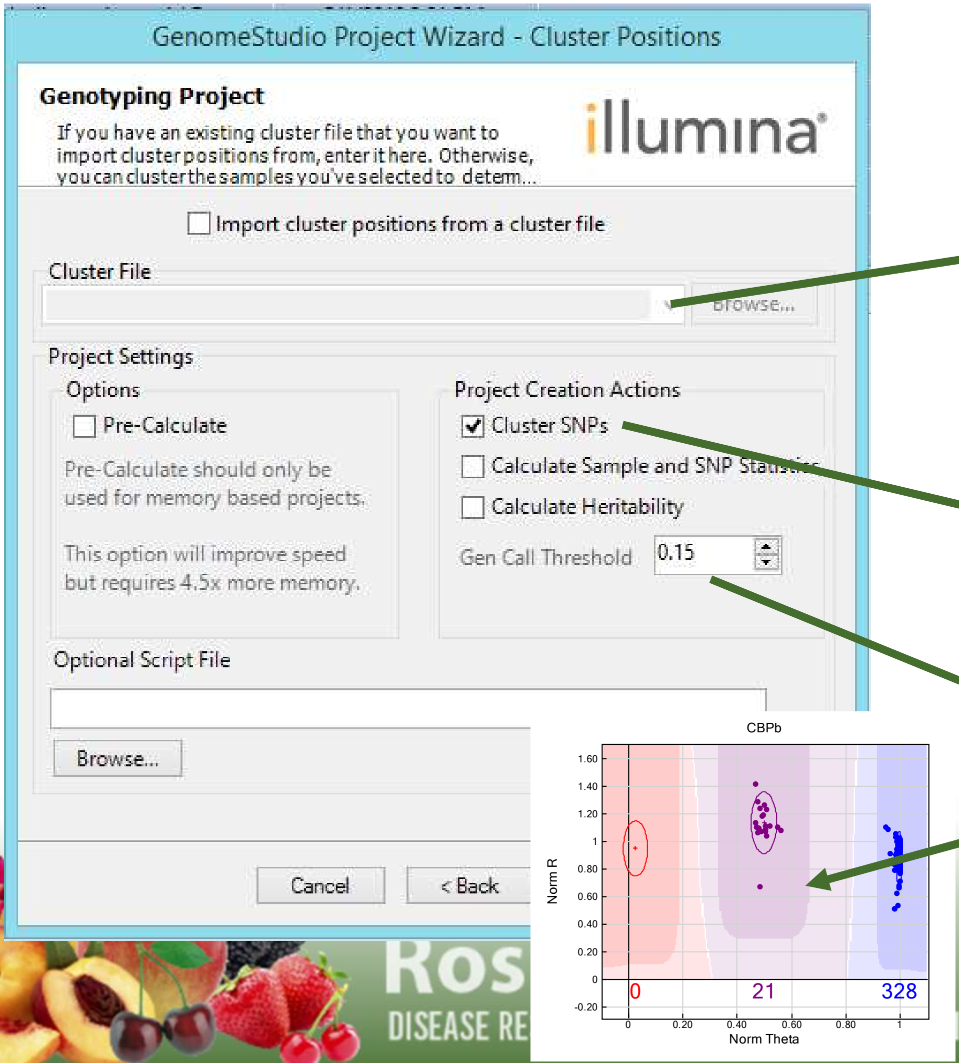

A file designating custom or pre-set cluster positions can be loaded here instead of the default auto-clustering

Cluster SNPs

-0.15 is standard
-Defines area within which individuals will get a genotype call (outside will be 'no calls')

Adding additional samples

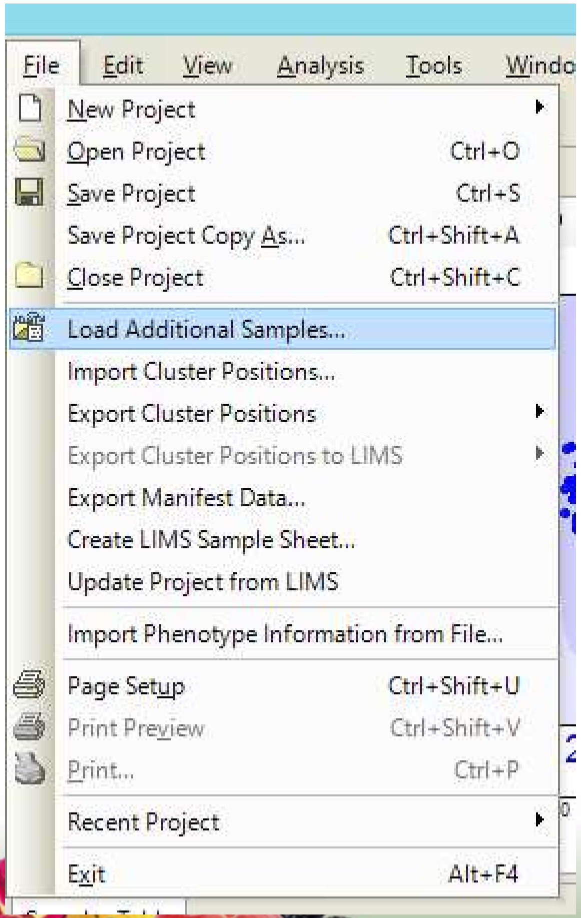

- File > Load Additional Samples…
  - Same process as before

RosBREED
DISEASE RESISTANCE × HORTICULTURAL QUALITY

United States Department of Agriculture

National Institute of Food and Agriculture

First look at data

RosBREED

DISEASE RESISTANCE × HORTICULTURAL QUALITY → SUPERIOR CULTIVARS

GenomeStudio® Layout

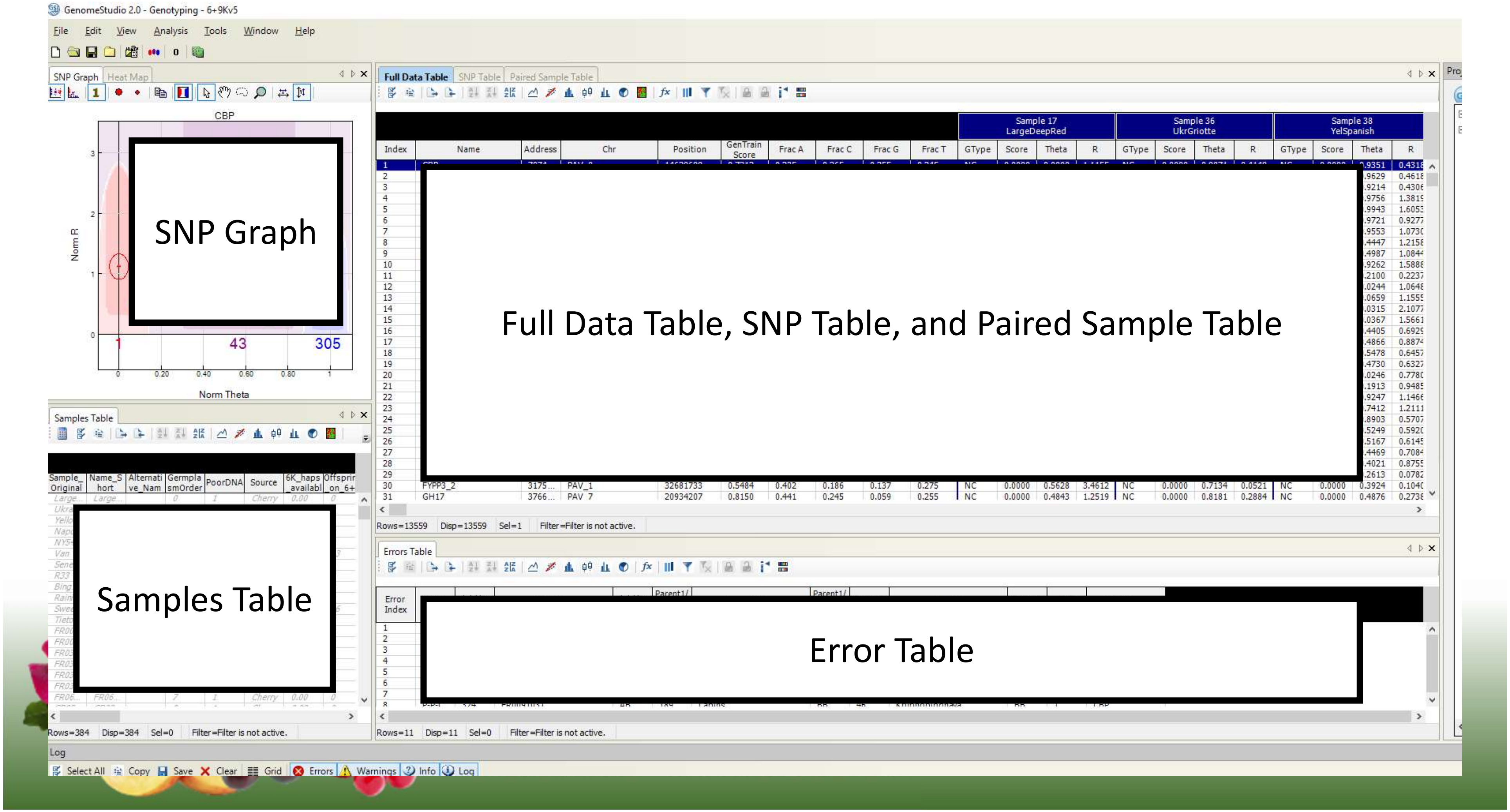

SNP Graph

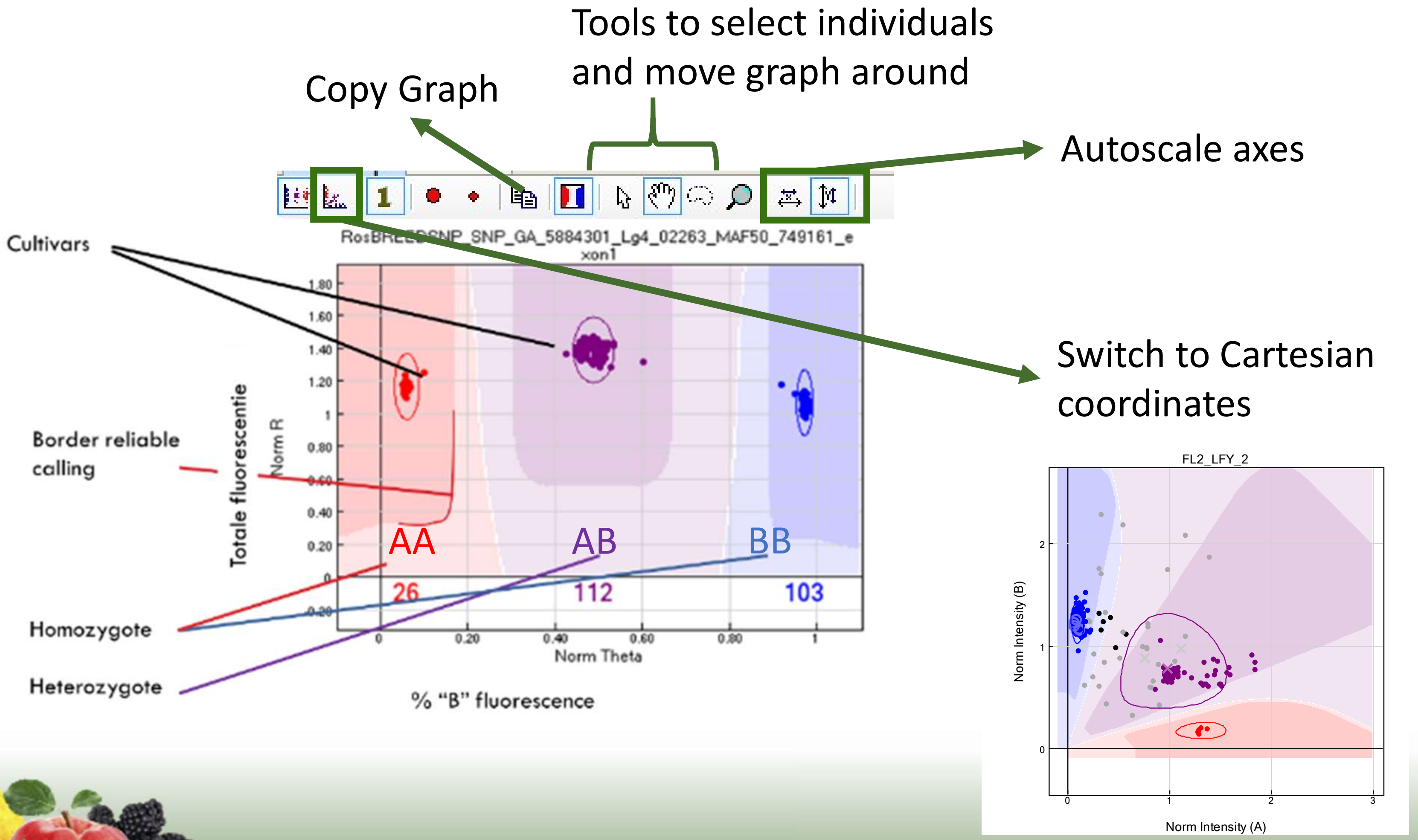

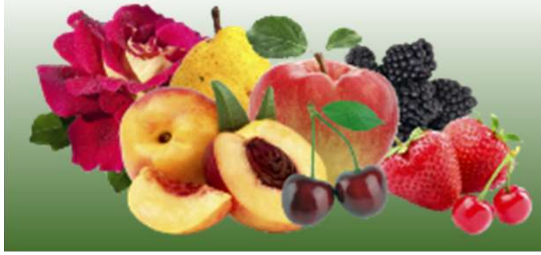

RosBREED

DISEASE RESISTANCE × HORTICULTURAL QUALITY

Full data table

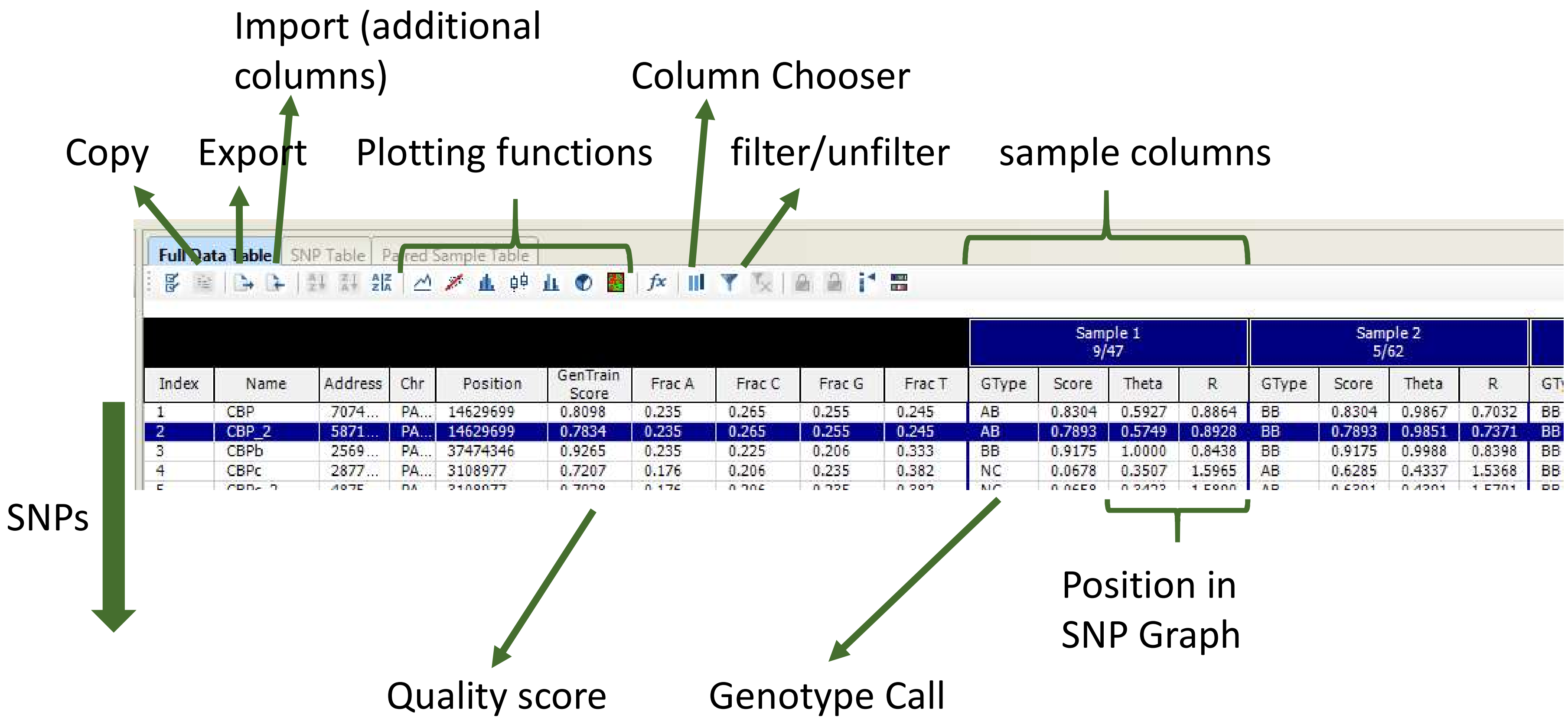

SNP data table

Full Data Table | **SNP Table** | Paired Sample Table

Index	Name	Chr	Position	ChiTest100	Het Excess	AA Freq	AB Freq	BB Freq	Call Freq	Minor Freq	Aux	P-C Errors	P-P-C Errors	Rep Errors	10% GC	50% GC	SNP	# Calls	# no calls	Plus/Minus Strand	Custom Cluster #	Ad
1	CBP	PA...	14629699	0.8328	0.0211	0.0029	0.1232	0.8739	1.0000	0.0645	0	0	1	0	0.8304	0.8304	[T/C]	349	0		3	70
2	CBP_2	PA...	14629699	0.8328	0.0211	0.0029	0.1232	0.8739	1.0000	0.0645	0	0	1	0	0.7893	0.7893	[T/C]	349	0		3	58
[illegible]	[illegible]	[illegible]	[illegible]	[illegible]	[illegible]	[illegible]	[illegible]	[illegible]	[illegible]	[illegible]	[illegible]	[illegible]	[illegible]	[illegible]	[illegible]	[illegible]	[illegible]	[illegible]	[illegible]		[illegible]	[illegible]

How well did the SNP perform?

Are parents and offspring matching? (see later)

Was the SNP polymorphic?

Full Data Table | **SNP Table** | Paired Sample Table

SNP	# Calls	# no calls	Plus/Minus Strand	Custom Cluster #	Address	GenTrain Score	Orig Score	Edited	Cluster Sep	AA T Mean	AA T Dev	AB T Mean	AB T Dev	BB T Mean	BB T Dev	AA R Mean	AA R Dev	AB R Mean	AB R Dev	BB R Mean	BB R Dev	Address2	Norm ID
					RosBREED_Cherry_15k_15069617X343343_A1.bpm																		
[T/C]	349	0		3	7074231	0.8098	0.8098	0	0.9001	0.0706	0.0224	0.5832	0.0346	0.9914	0.0074	0.4874	0.1000	0.9331	0.1088	0.7781	0.1000	0	2
[T/C]	349	0		3	5871731	0.7834	0.7834	0	0.9221	0.0760	0.0224	0.5850	0.0328	0.9888	0.0075	0.4652	0.1000	0.9608	0.1093	0.7820	0.1000	0	2

“Array specific” data

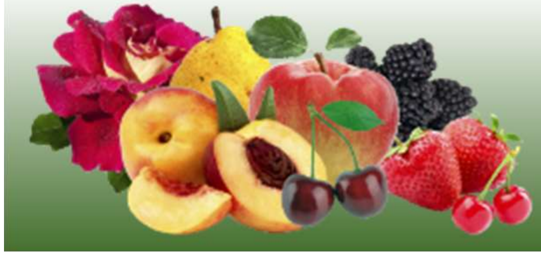

Sample Table

- All columns of sample sheet
- Sample statistics (need to calculated)
- Linked with SNP graph
    - Selecting in table will highlight in SNP g
      vice versa

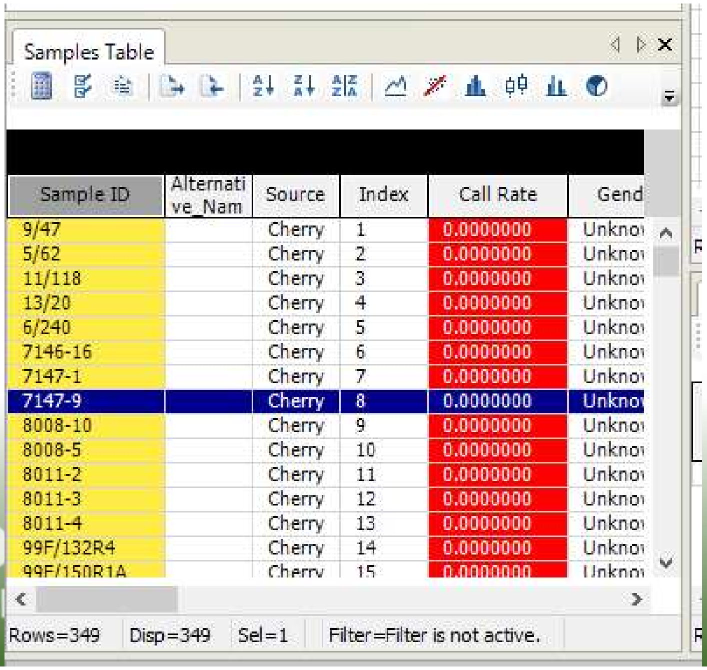

Samples Table

Sample ID	Alternative_Nam	Source	Index	Call Rate	Gend
9/47		Cherry	1	0.0000000	Unknov
5/62		Cherry	2	0.0000000	Unknov
11/118		Cherry	3	0.0000000	Unknov
13/20		Cherry	4	0.0000000	Unknov
6/240		Cherry	5	0.0000000	Unknov
7146-16		Cherry	6	0.0000000	Unknov
7147-1		Cherry	7	0.0000000	Unknov
7147-9		Cherry	8	0.0000000	Unknov
8008-10		Cherry	9	0.0000000	Unknov
8008-5		Cherry	10	0.0000000	Unknov
8011-2		Cherry	11	0.0000000	Unknov
8011-3		Cherry	12	0.0000000	Unknov
8011-4		Cherry	13	0.0000000	Unknov
99F/132R4		Cherry	14	0.0000000	Unknov
99F/150R1A		Cherry	15	[illegible]	[illegible]

Rows=349 Disp=349 Sel=1 Filter=Filter is not active.

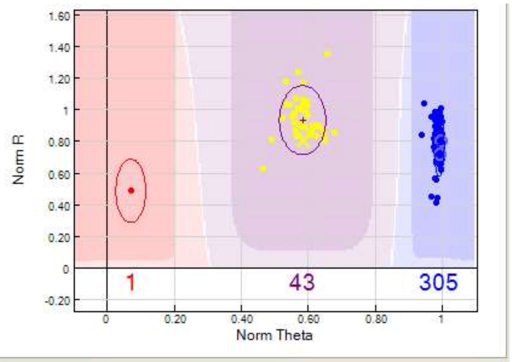

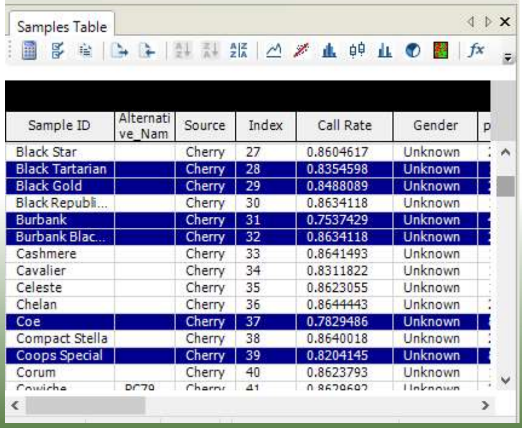

Samples Table

Sample ID	Alternative_Nam	Source	Index	Call Rate	Gender
Black Star		Cherry	27	0.8604617	Unknown
Black Tartarian		Cherry	28	0.8354598	Unknown
Black Gold		Cherry	29	0.8488089	Unknown
Black Republi...		Cherry	30	0.8634118	Unknown
Burbank		Cherry	31	0.7537429	Unknown
Burbank Blac...		Cherry	32	0.8634118	Unknown
Cashmere		Cherry	33	0.8641493	Unknown
Cavalier		Cherry	34	0.8311822	Unknown
Celeste		Cherry	35	0.8623055	Unknown
Chelan		Cherry	36	0.8644443	Unknown
Coe		Cherry	37	0.7829486	Unknown
Compact Stella		Cherry	38	0.8640018	Unknown
Coops Special		Cherry	39	0.8204145	Unknown
Corum		Cherry	40	0.8623793	Unknown
Cowiche	[illegible]	Cherry	41	[illegible]	Unknown

Error Table

- Shows errors between replicates and parent(s) and offspring
  - Will be discussed further
  - Linked to SNP graph
    - Replicates: square
    - Parent(s) and offspring: circle(s) and cross, respectively

Errors Table

Error Index	Error Type	Child/Rep Index	Child/Rep	Child/Rep GType	Parent1/Rep Index	Parent1/Rep	Parent1/Rep GType	Parent2 Index	Parent2	Parent2 GType	SNP Index	SNP Name
1	P-C	110	FR008T049	AA	234	Krupnoplodnaya	BB				8	COL2
2	P-P-C	118	FR012T061	AA	238	Lapins	AB	341	Venus	BB	8	COL2
3	P-P-C	221	Gil-Peck	AB	256	Napoleon	BB	220	Giant	BB	8	COL2

Error Index	Error Type	Child/Rep Index	Child/Rep	Child/Rep GType	Parent1/Rep Index	Parent1/Rep	Parent1/Rep GType	Parent2 Index	Parent2	Parent2 GType	SNP Index	SNP Name
1	P-C	110	FR008T049	AA	234	Krupnoplodnaya	BB				8	COL2
2	P-P-C	118	FR012T061	AA	238	Lapins	AB	341	Venus	BB	8	COL2
3	P-P-C	221	Gil-Peck	AB	256	Napoleon	BB	220	Giant	BB	8	COL2

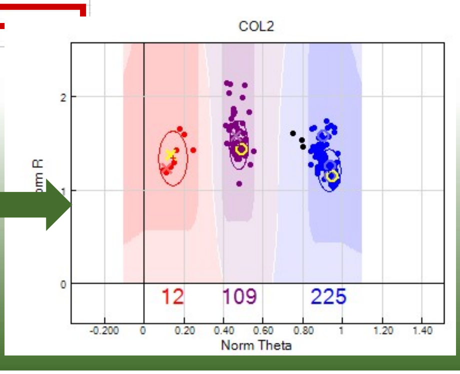

DISEASE RESISTANCE × HORTICULTURAL QUALITY

USDA

United States Department of Agriculture

National Institute of Food and Agriculture

Sample quality and ploidy

RosBREED

DISEASE RESISTANCE × HORTICULTURAL QUALITY → SUPERIOR CULTIVARS

Checking Sample Quality

- Visual inspection of SNP graphs

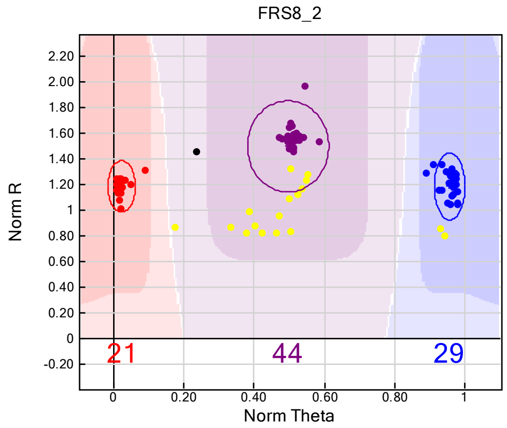

Some individuals are regularly located outside main clusters

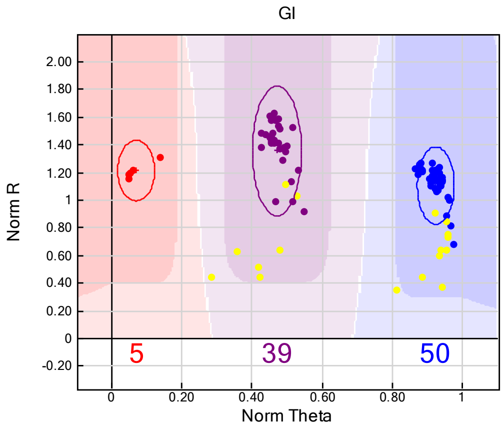

More individuals outside main clusters
*use ‘ctrl’ + left mouse clicks to add new individual to selection

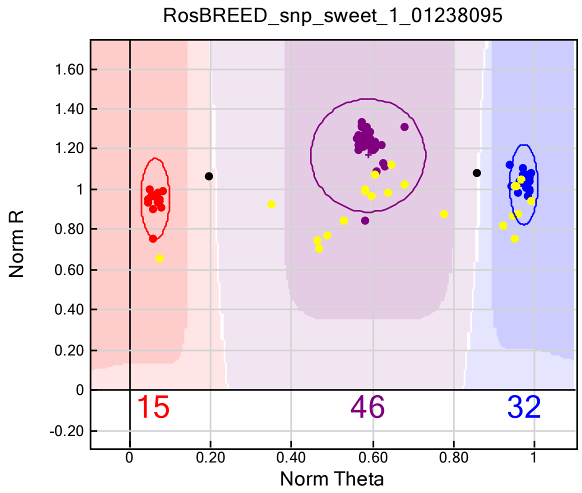

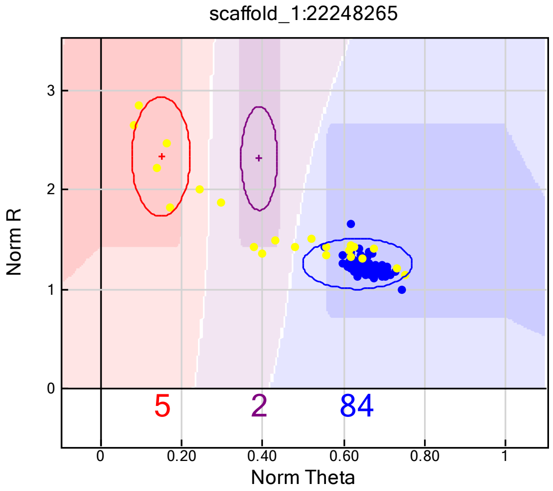

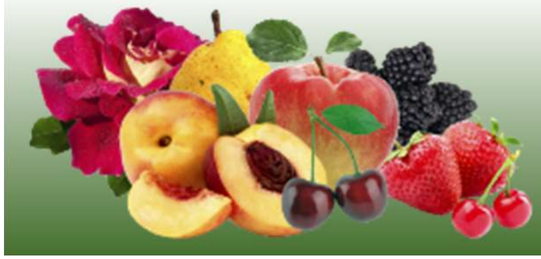

RosBREED
DISEASE RESISTANCE × HORTICULTURAL QUALITY

Checking Sample Quality

1. Histogram function of Full Data Table

Options greyed out?!

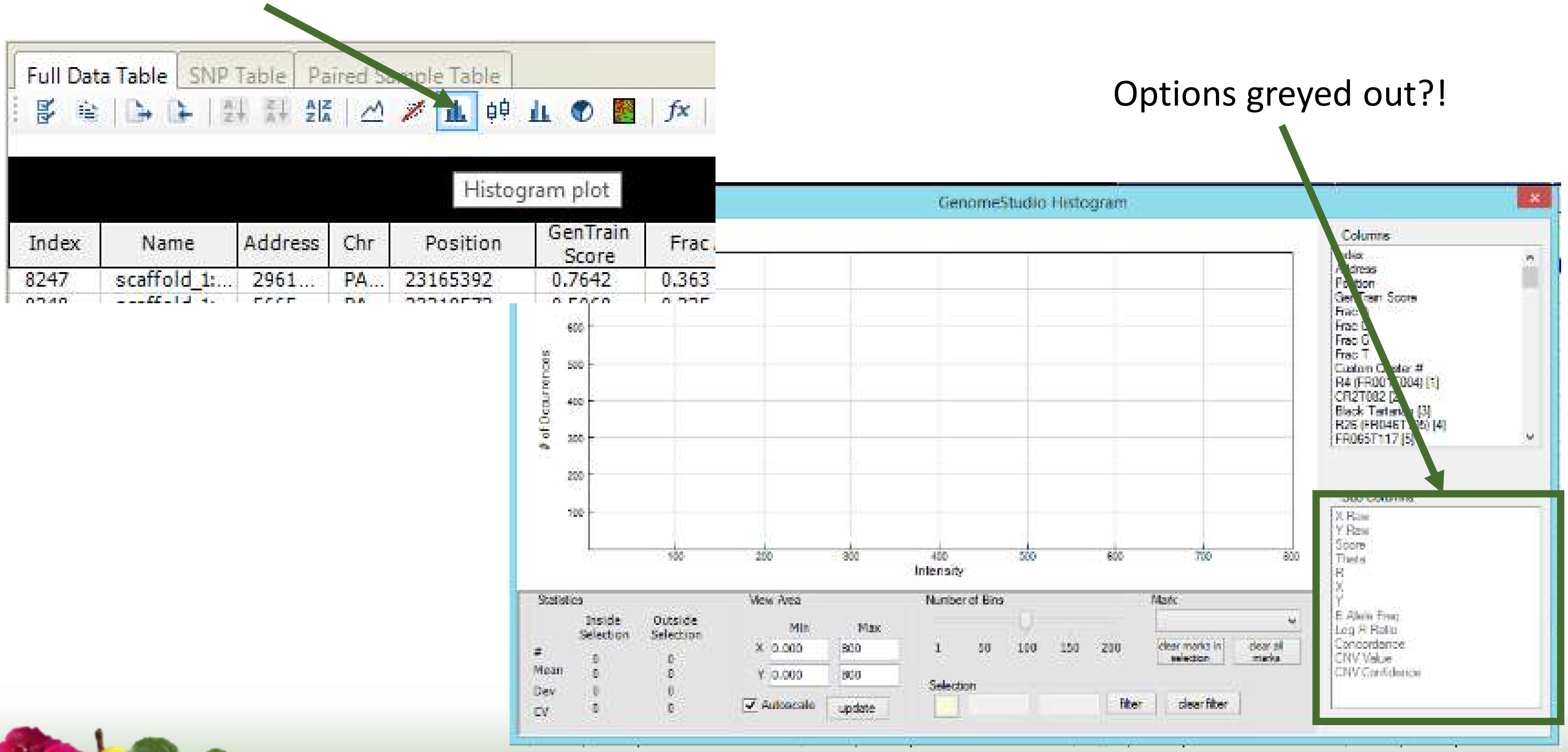

Checking Sample Quality

2. Select first individual

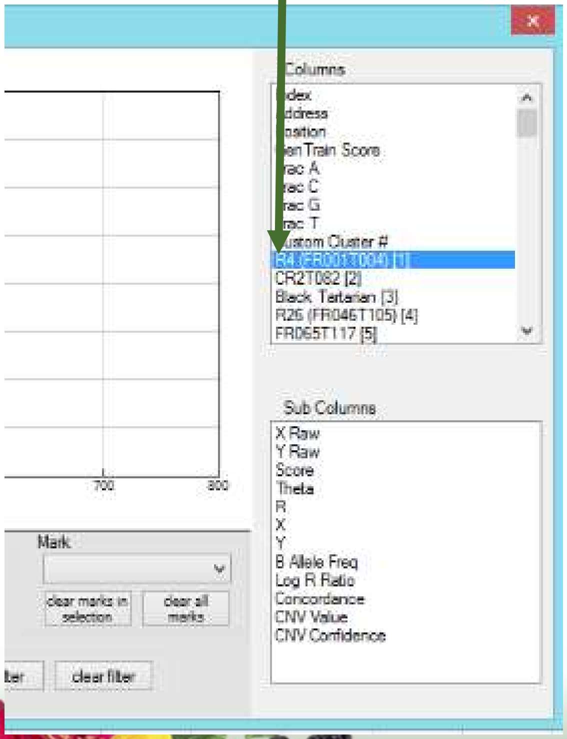

3. Choose “B Allele Freq”

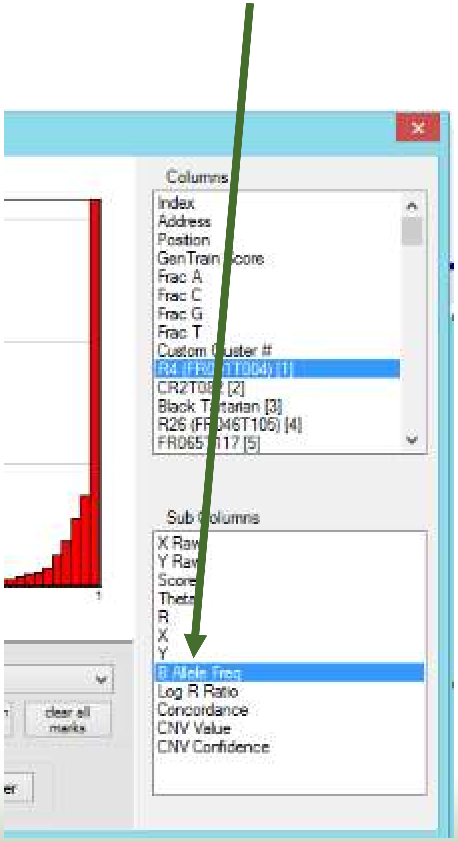

4. Select individual again
*Allows for scrolling through ind.
*Use ‘up’ and ‘down’ keys to scroll through ind.

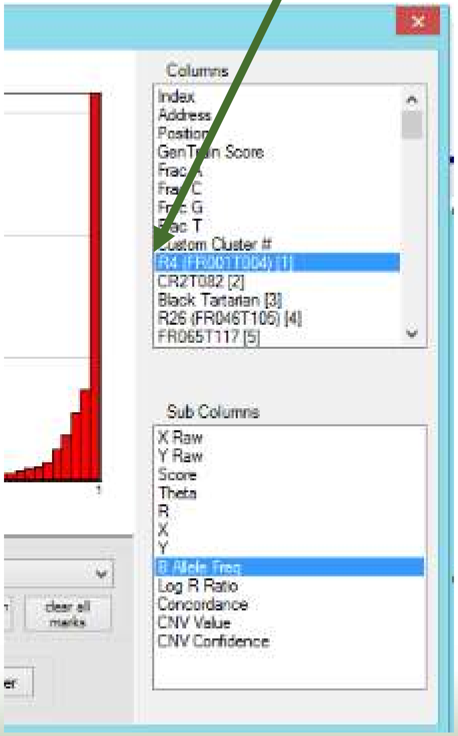

Checking Sample Quality

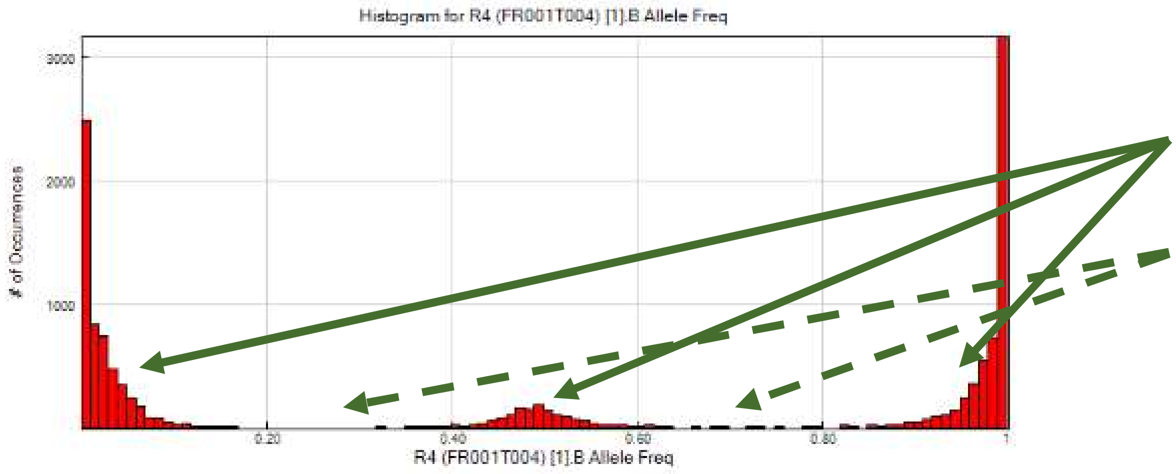

Sample with good quality

-Three (clear) peaks

-Barely any occurrences between three peaks

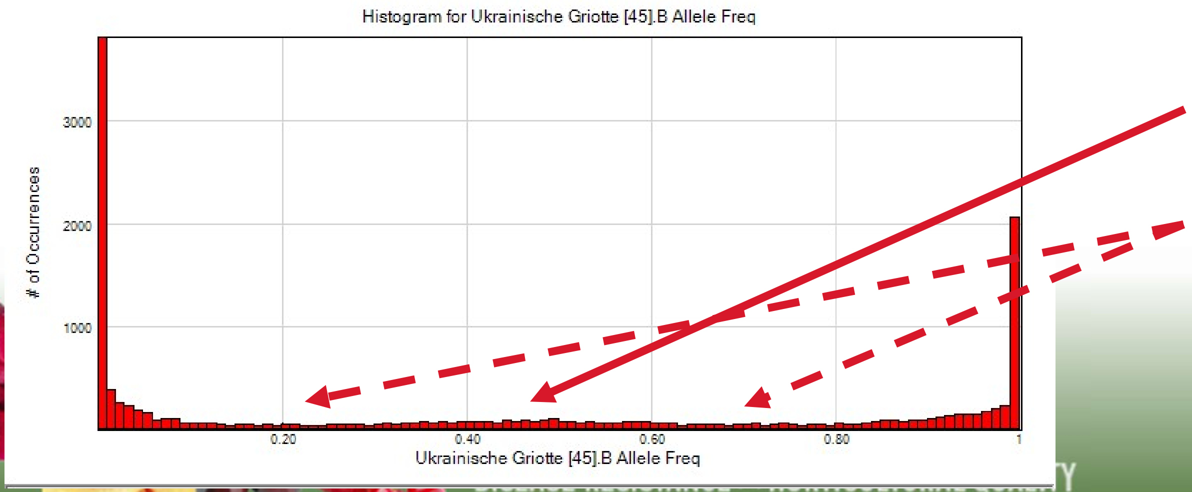

Sample with poor quality

-No clear heterozygous peak

-Many occurrences between three peaks
(middle section looks ~ flat)

Checking Sample Ploidy

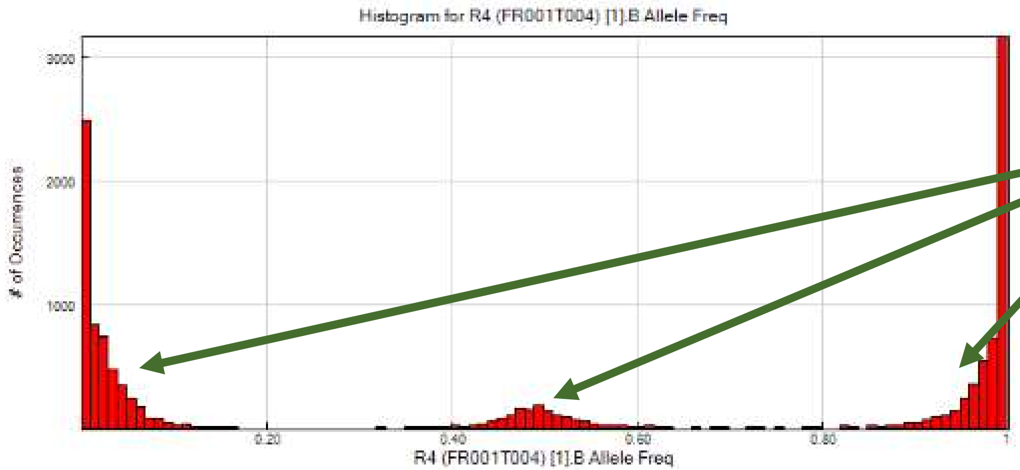

Diploid sample

-Three (clear) peaks
-- AA, AB, and BB

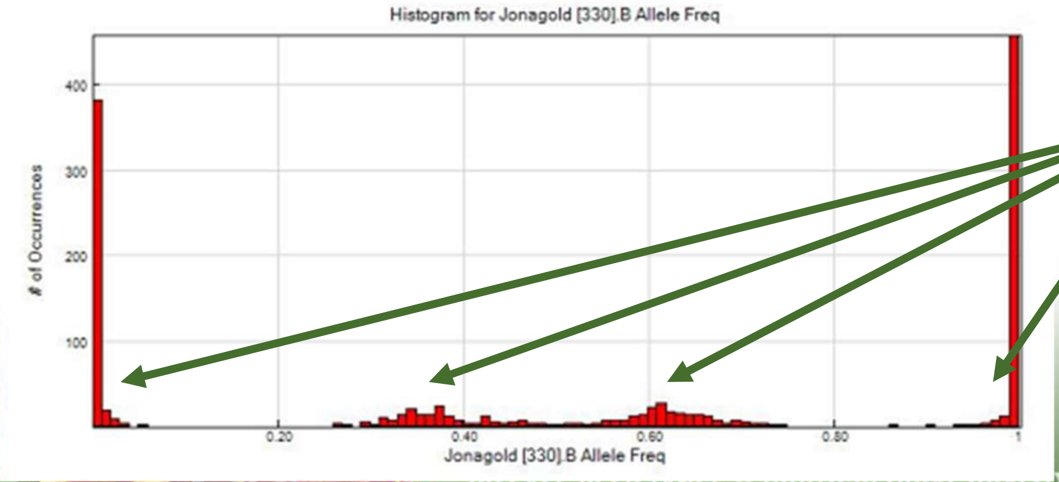

Triploid sample

-Four peaks
--AAA, AAB, ABB, and BBB

Checking Sample Ploidy

- Other ploidy

Tetraploid sample

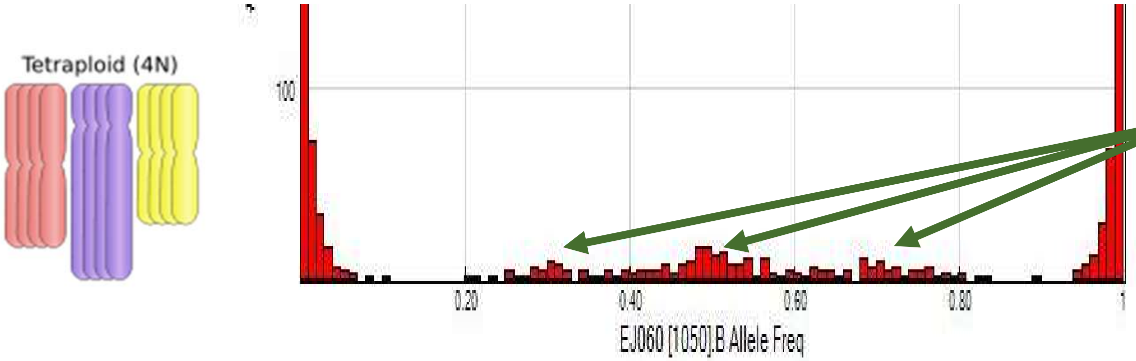

-Five peaks?

-Different from poor quality?

-Could be a mix of 2 samples

Aneuploid sample

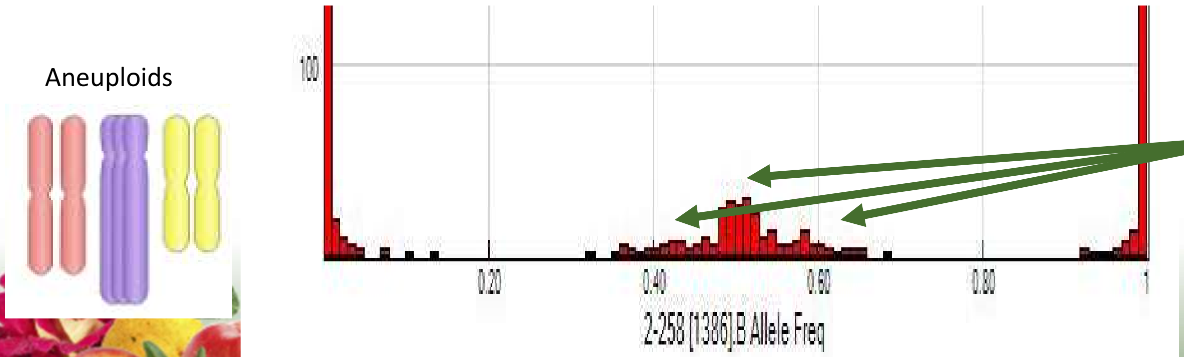

-3 peaks + 2 small peaks?

-Not easy to see

DISEASE RESISTANCE × HORTICULTURAL QUALITY

Chagné et al. (2015) method

1. Choose ‘Filter rows’ for SNP Table in GenomeStudio®

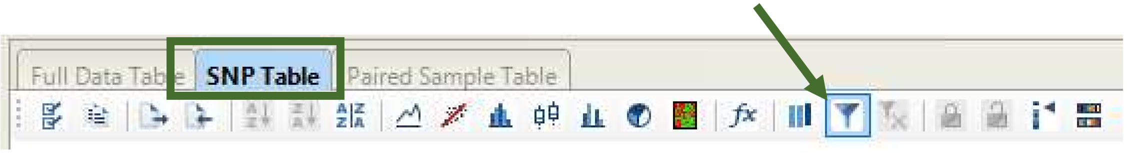

2. Apply the following filter parameters (continued on next slide):

Call Freq	≥ 0.90	Only SNP that have good call rate
Minor Freq	>0.01	Only polymorphic SNPs
50% GC	≥ 0.40	Only SNPs for which most individuals were close to main cluster
GenTrain	≥ 0.65	Only SNPs with good overall clustering
ClusterSep	≥ 0.40	Only SNPs with well separated clusters
AA T Mean	≤ 0.125	Only SNPs with AA cluster in expected position
AA T Dev	≤ 0.028	Only SNPs with 'narrow' AA cluster (no multiple clusters)
AB T Mean	≥ 0.375 ≤ 0.625	Only SNPs with AB cluster in expected position
AB T Dev	≤ 0.056	Only SNPs with 'narrow' AB cluster (no multiple clusters)
BB T Mean	≥ 0.875	Only SNPs with BB cluster in expected position
BB T Dev	≤ 0.028	Only SNPs with 'narrow' BB cluster (no multiple clusters)

Chagné et al. (2015) method

2. Apply the following filter parameters (continued):

2a. Choose parameter

2b. Choose operation

2c. Choose value

2d. Add to filter criteria

2e. Add remaining criteria
"AND" clause is added automatically

2f. Click 'OK' when <u>all</u> criteria are added

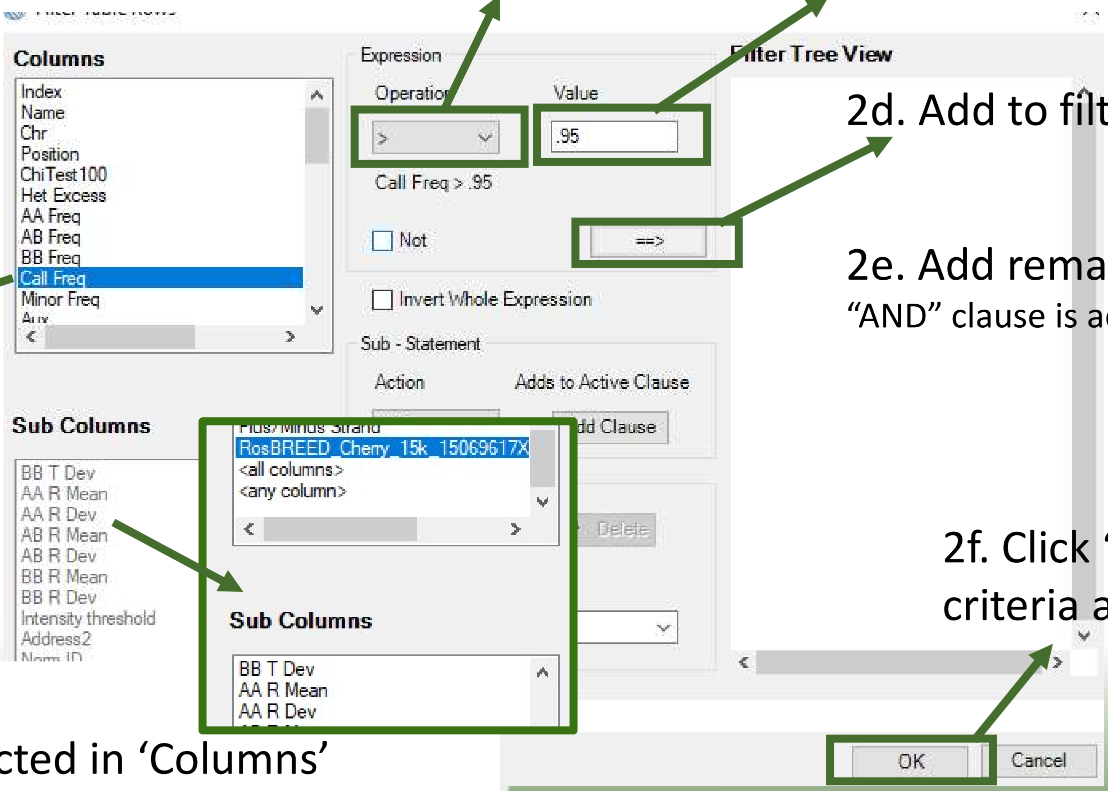

<u>Note:</u>
Manifest must be selected in 'Columns' section before subcolumns become available

Chagné et al. (2015) method

3. Go to Full Data Table and select ‘Column Chooser’
*Filtering of SNP Table remains for Full Data Table

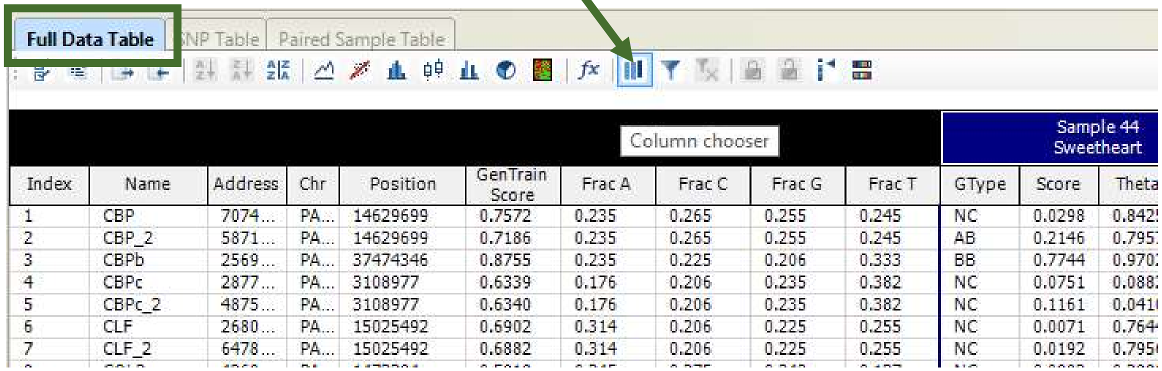

4. Hide any column except Index, Chr, Position, and sample columns

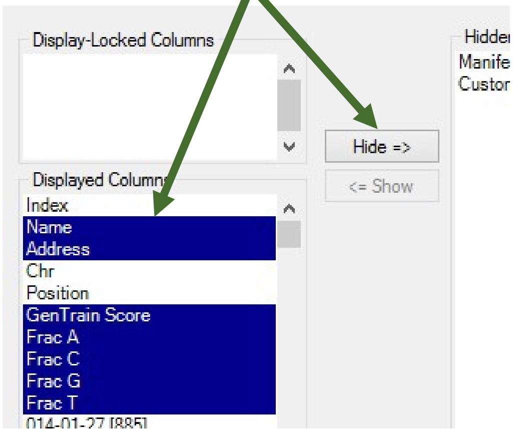

6. Show B Allele Freq subcolumn

5. Hide standard subcolumns

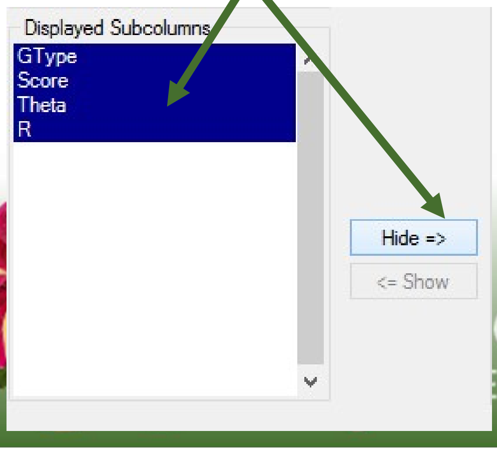

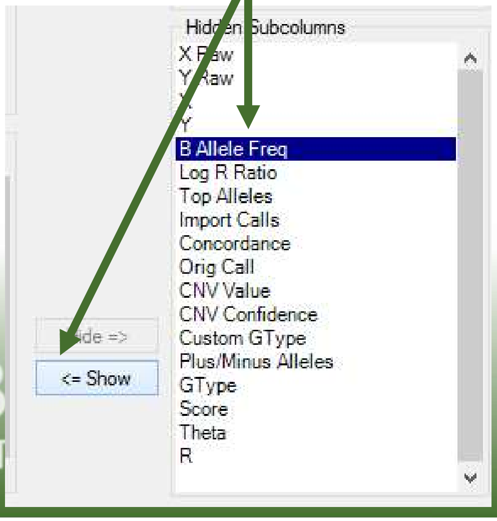

7. Press ‘OK’

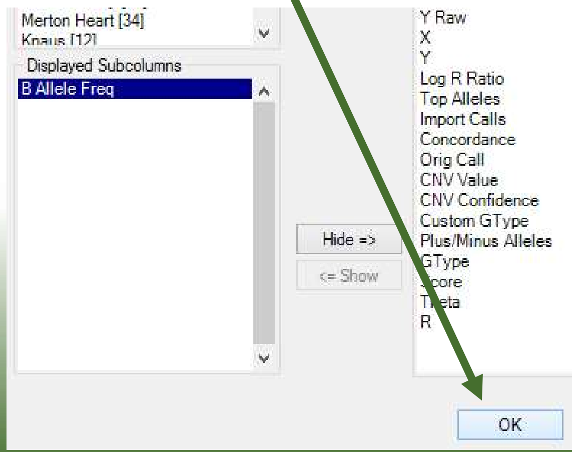

Chagné et al. (2015) method

8. Export table

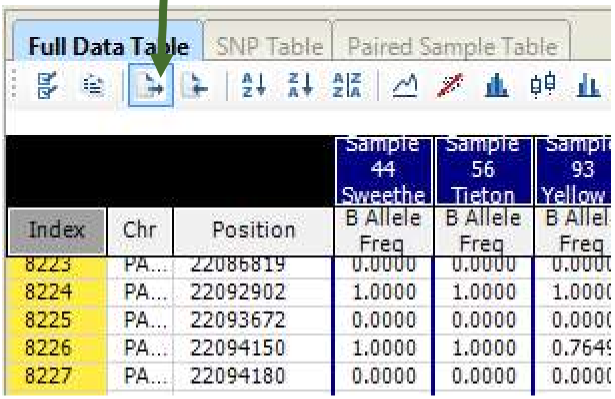

9. Give a meaningful name, choose save location, and save as '.csv' file

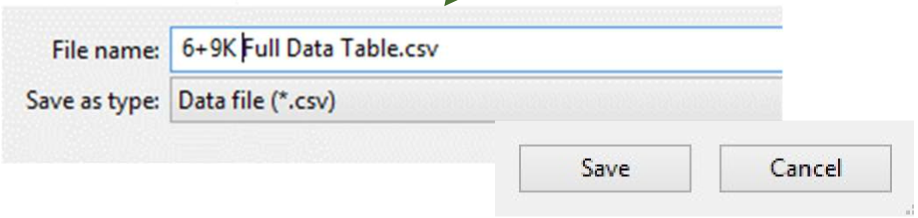

10. If asked, choose 'Yes' to export entire table

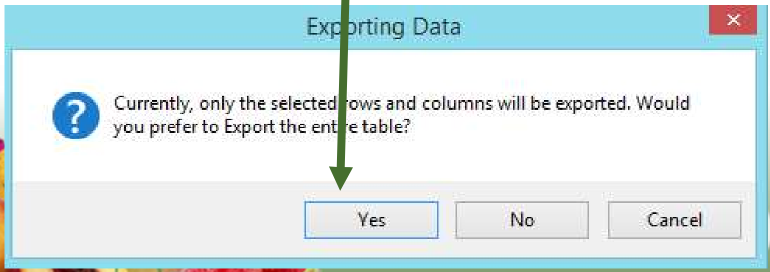

11. Choose 'Yes' to view file in Excel

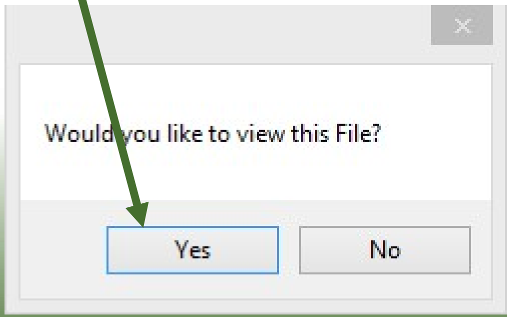

DISEASE RESISTANCE × HORTICULTURAL QUALITY

Chagné et al. (2015) method

12. Convert Chromosome numbers to numeric system where applicable

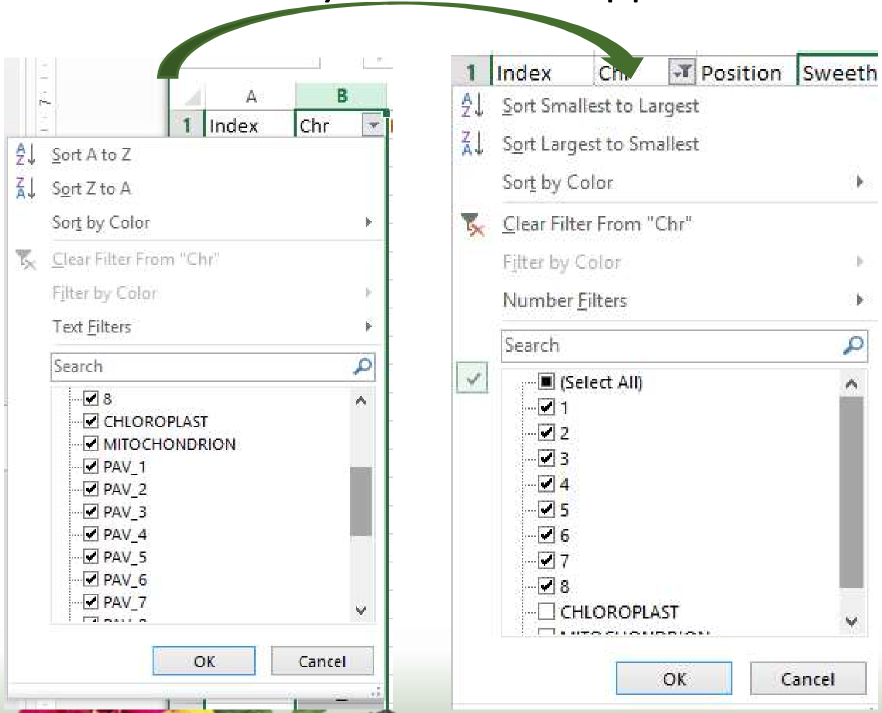

13. Insert a new column in front of sample columns

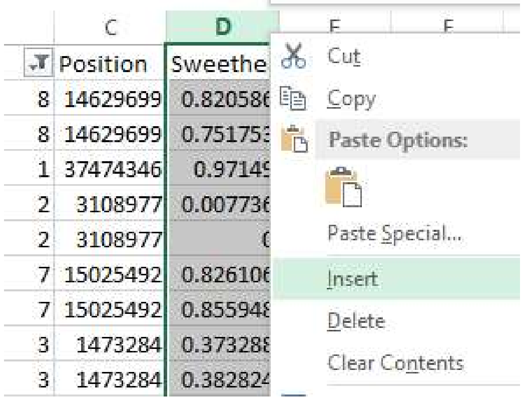

RosBREED
DISEASE RESISTANCE × HORTICULTURAL QUALITY

Chagné et al. (2015) method

14. Apply the following formula for the new column:

Cell value = 1,000,000,000 * ‘Chromosome number’ + ‘position on chromosome’

*Choose a power of 10 that is larger than any position on chromosome

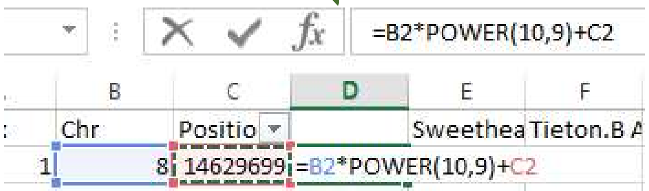

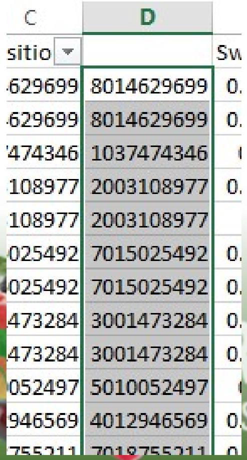

15. Replace formula by fixed values

*Copy column and paste as values

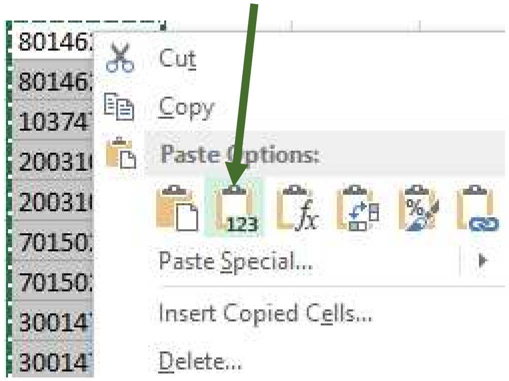

16. Delete original chromosome and position column

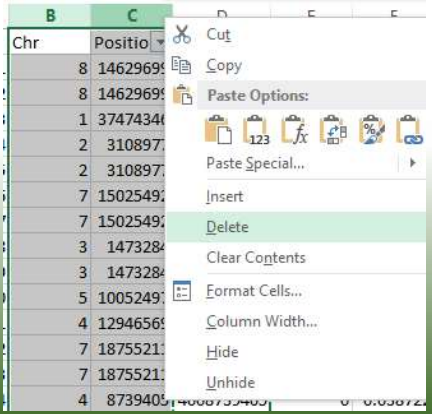

RosBREED
DISEASE RESISTANCE × HORTICULTURAL QUALITY

Chagné et al. (2015) method

File should look like this:
- 1st column: SNP Identifier (e.g. SNP Index)
- 2nd column: SNP position (including chromosome)
- 3rd column – end: B-allele Freq of each individual

A	B	C	D	E	F	
Index	Position	Sweethea	Tieton.B A	Yellow Sp	Rainier.B A	Ukr
1	8014629699	0.820586	0.658852	0.935553	0.926576	0.[illegible]
2	8014629699	0.751753	0.752404	0.970744	0.808932	0.[illegible]
3	1037474346	0.97149	0.817936	0.918174	0.964891	0.[illegible]
4	2003108977	0.007736	0.63033	1	0.637792	
5	2003108977	0	0.699511	1	0.723935	

17. Save as new '.csv' file:

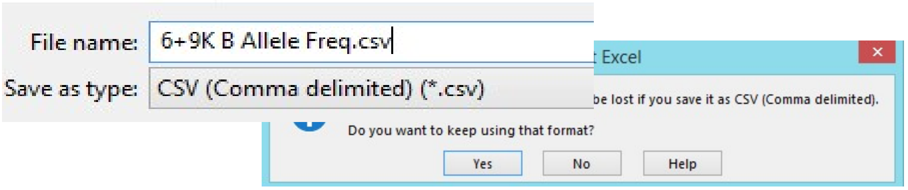

18. Copy R-script 'bAllele Analysis.R' (Suppl. Document 2) in same folder as newly created .csv file

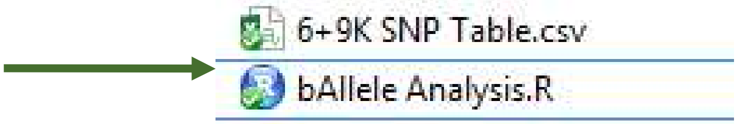

19. Open R-script 'bAllele Analysis.R' in RStudio

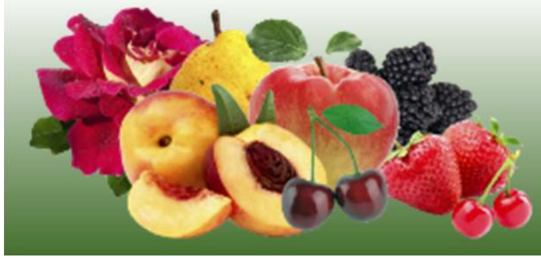

Chagné et al. (2015) method

20. Change file-name on line 1 into correct name of newly created .csv file

```
bAllele<- read.csv(file="SNP data_Triploids and deviations.csv", sep=",", stringsAsFactors=F)
```

```
bAllele<- read.csv(file="6+9K B Allele Freq.csv", sep=",", stringsAsFactors=F)
```

21. Change name of pdf-output on line 2 into desired name

```
pdf("bAllele.pdf")
```

```
pdf("bAllele 6+9K.pdf")
```

22. Change working directory into location that has .csv file and R script

*Under 'Session', choose 'Set Working Directory', then choose 'To Source File Location'

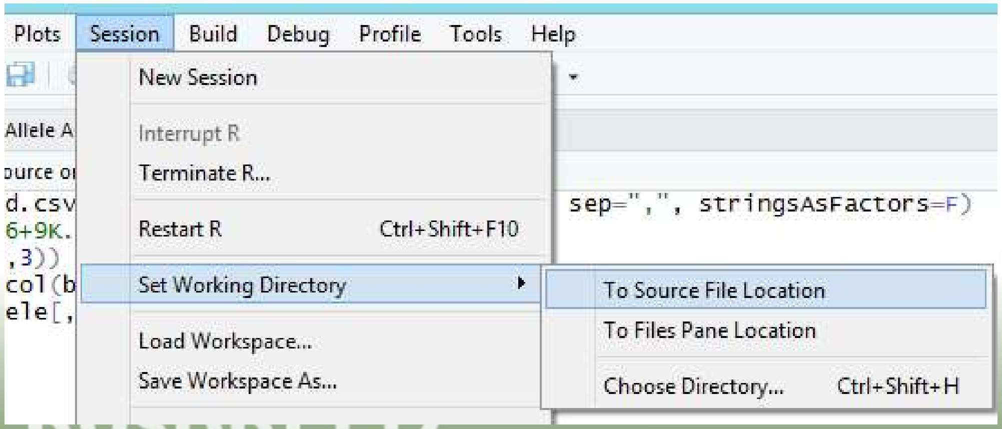

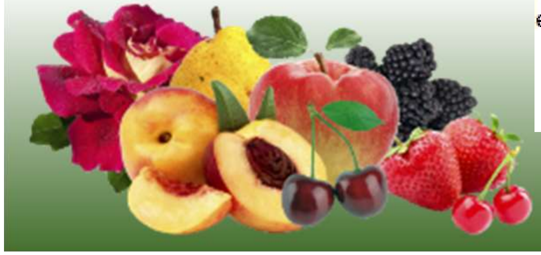

Chagné et al. (2015) method

23. Run entire script
*Select all lines and press run

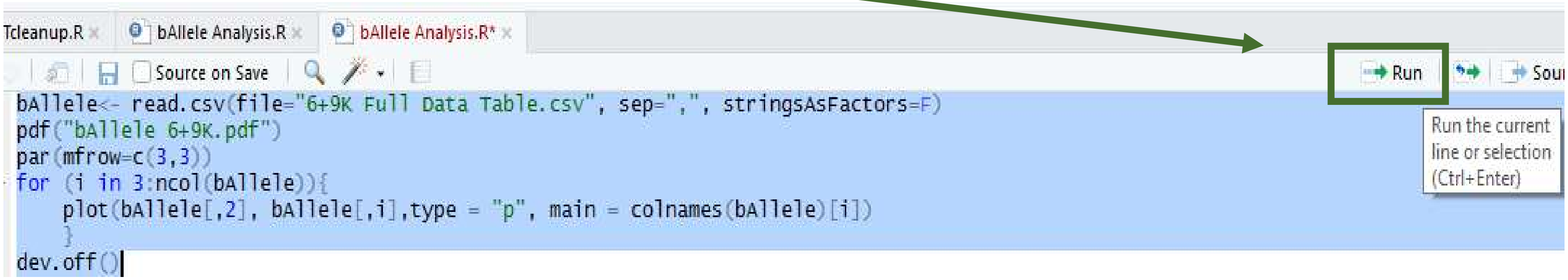

24. Open created pdf file
*It's in the same folder as the R-script and the .csv file with the B Allele Frequencies

<u>Poor quality</u>

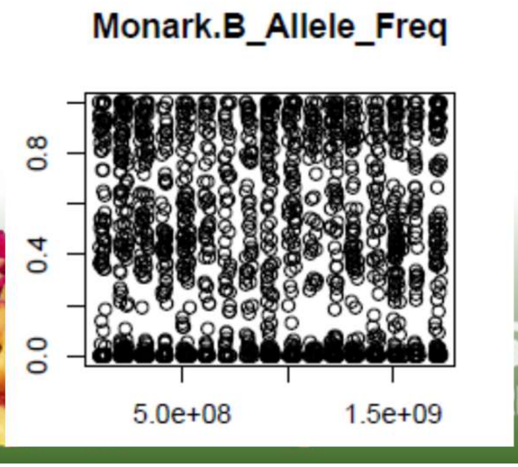

<u>Tetraploid?</u>

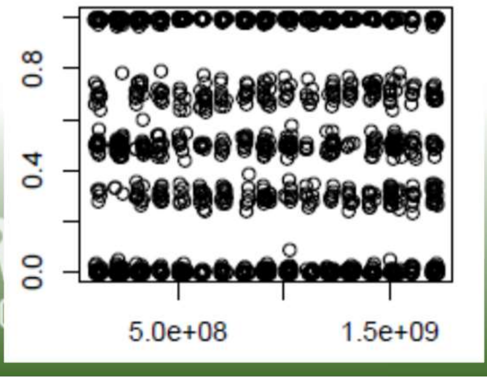

<u>Aneuploid</u>

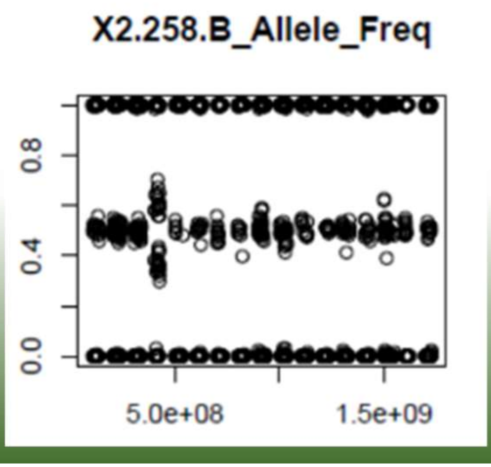

Chagné et al. (2015) method

Without initial filtering in SNP Table

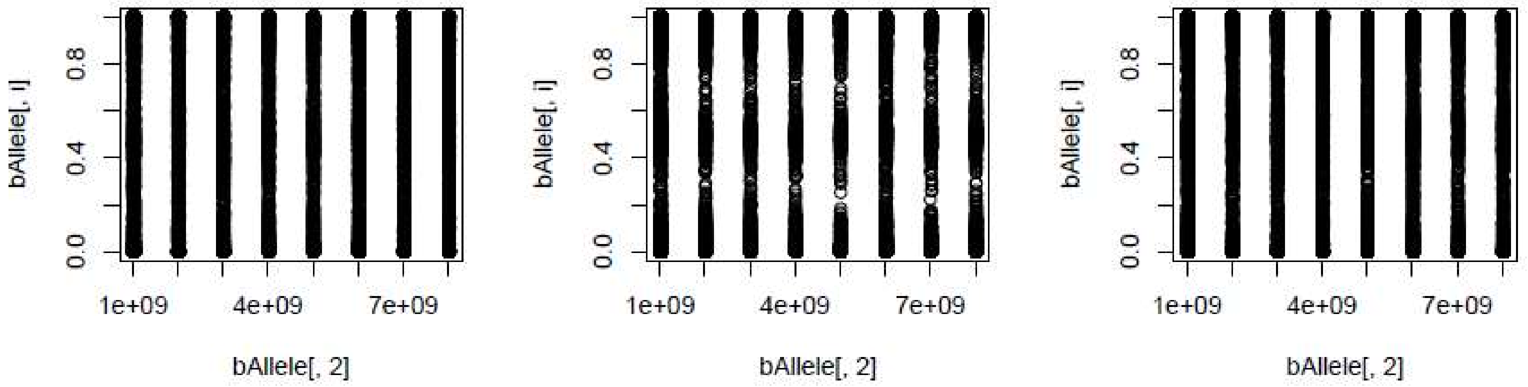

With initial filtering in SNP Table

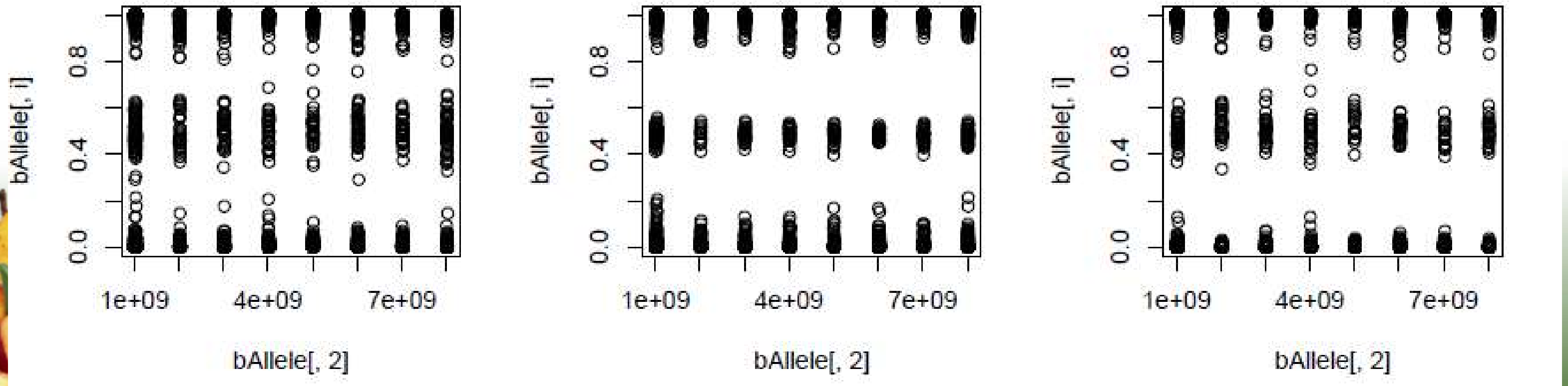

Segmental aneuploids

- Lack a large segment of a chromosome
  - Large extra segment?

- Cannot be identified with methods described above

- Will lead to many errors further on for one chromosomal segment

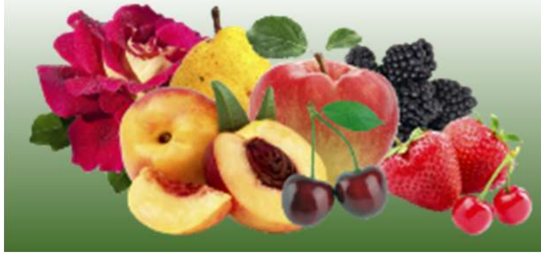

RosBREED
DISEASE RESISTANCE × HORTICULTURAL QUALITY

United States Department of Agriculture

National Institute of Food and Agriculture

SNP subset

RosBREED

DISEASE RESISTANCE × HORTICULTURAL QUALITY → SUPERIOR CULTIVARS

Creating input files ASSIsT

- Manual can be found under '…\ASSIsT_Win_v1.01\docs'

Creating 1st input file (Final report)

1. Open Report Wizard in GenomeStudio

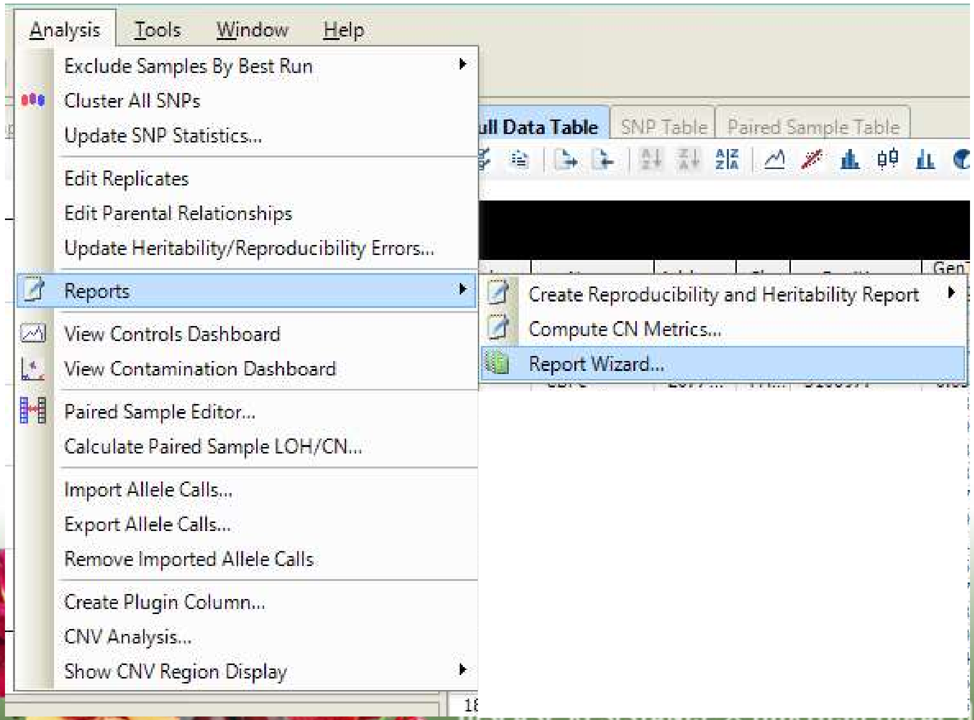

2. Choose Final Report

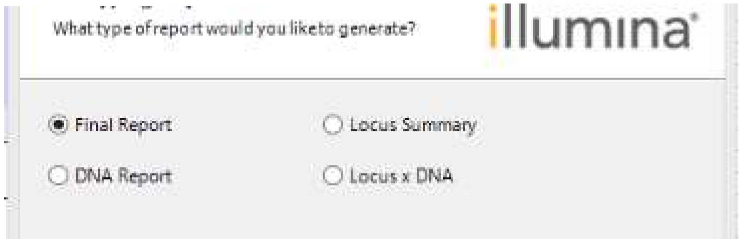

3. Choose 'redo with the best' and '10th Percentile GC score'

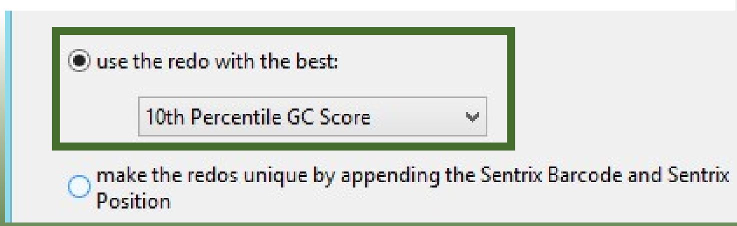

Creating input files ASSIsT

4. Select all samples/arrays

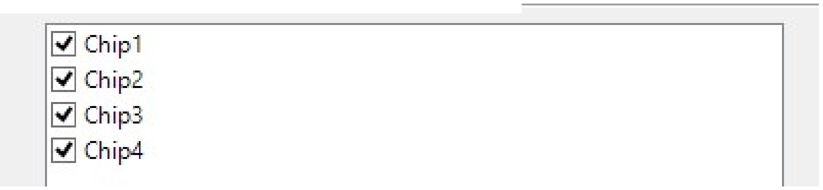

5.-Set format to Standard

-Include the following columns:

GT Score, Theta, R

*Order should be the same as figure

-Select 'Group by SNP'

-Select 'Tab' as delimiter

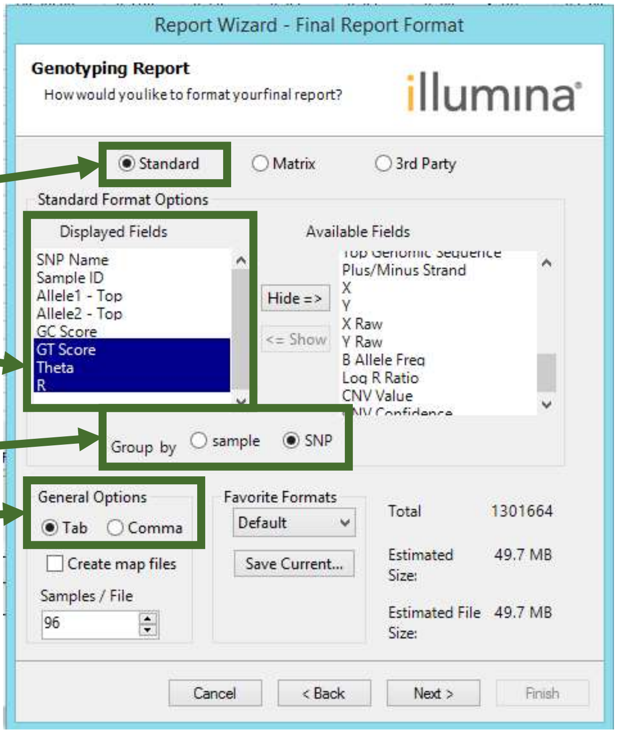

6. Save Report

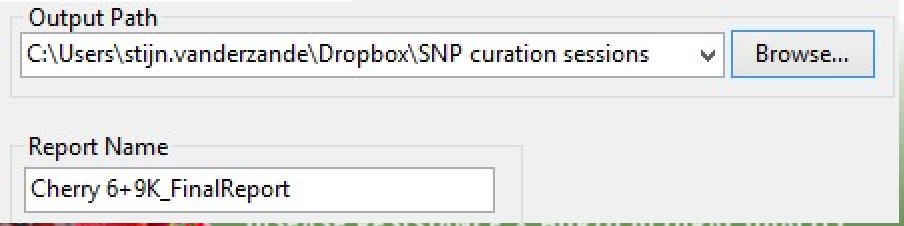

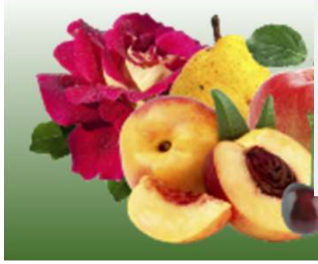

Creating input files ASSIsT

Creating 2nd input file (DNA report)

7. Open Report Wizard in GenomeStudio and choose DNA Report

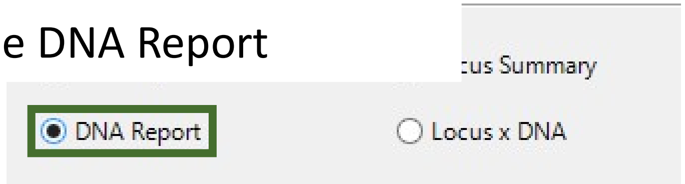

8. Choose ‘redo with the best’ and ‘10th Percentile GC score’

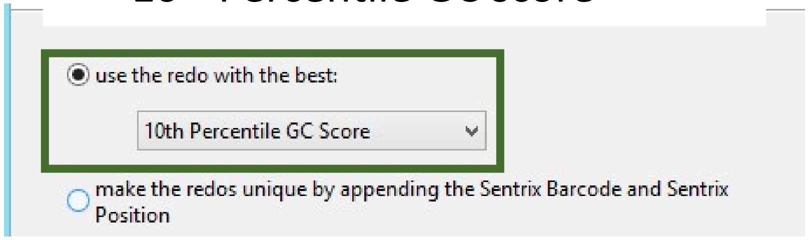

9. Select all samples/arrays

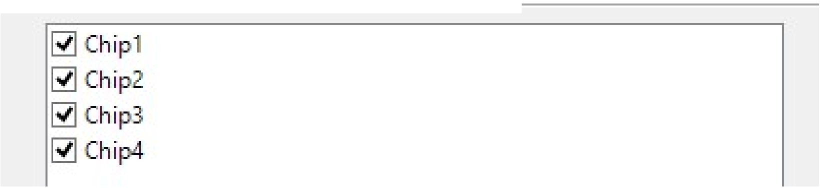

10. Choose Sample ID

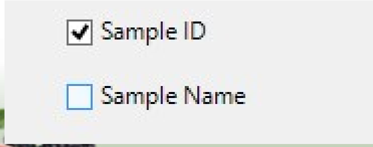

11. Save Report

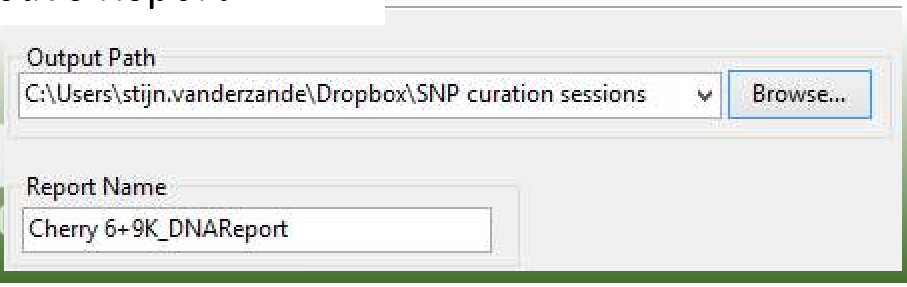

Input files ASSIsT

Creating 3rd input file (Pedigree file)

12. Create a three-column file:

- -1st column has the individual's name
- -2nd column has the female parent
- -3rd column has the male parent
- -Header row should be "//SampleID [tab] Mother [tab] Father"

*Copy SampleID and parent columns from sample sheet into Notepad++ and add the header line

*Parents do not have to be included in sample list

	A	B	C
1	//SampleID	Mother	Father
2	Abundance	Napoleon	
3	Corum		
4	Cuvelier		
5	Persian		
6	Benton	Stella	Moreau
7	Bing	BlackRepublican	Napoleon
8	BlackRepublican	Napoleon	BlackTartarian
9	BlackTartarian		
10	Lambert	Napoleon	Blackheart

```
//SampleID  Mother   Father
Abundance   Napoleon
Corum
Cuvelier
Persian
Benton  Stella  Moreau
Bing    BlackRepublican Napoleon
BlackRepublican Napoleon    BlackTartarian
BlackTartarian
Lambert Napoleon    Blackheart
```

DISEASE RESISTANCE × HORTICULTURAL QUALITY

Input files ASSIsT

Optional - Creating 4th input file (Map file)

13. Create a three-column file:

- -1st column has the SNP's name/SNP's ID
- -2nd column has the chromosome (numerical)
- -3rd column has the position (physical (bp, Mbp) or genetic (cM))
- -Header row should be "//SNPid [tab] Chromosome [tab] Position"

*Copy Name, Chr and Position from exported Full Data Table and convert chromosome to numeric if needed

*Use genetic position if available

*Give fictional chromosome number to chloroplast/mitochondrial/… SNPs

	A	B	C	D	E
1	//SNPid	Chromosome	Position		
2	CBP	8	14629699		
3	CBP_2	8	14629699		
4	CBPb	1	37474346		
5	CBPc	2	3108977		
6	CBPc_2	2	3108977		
7	[illegible]	[illegible]	[illegible]		

1	//SNPid	Chromosome	Position
2	CBP	8	14629699
3	CBP_2	8	14629699
4	CBPb	1	37474346
5	CBPc	2	3108977
6	CBPc_2	2	3108977
7	[illegible]	[illegible]	[illegible]

Running ASSIsT

1. Open ASSIsT

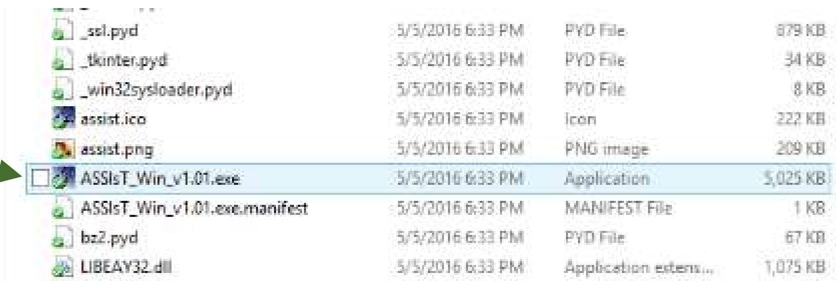

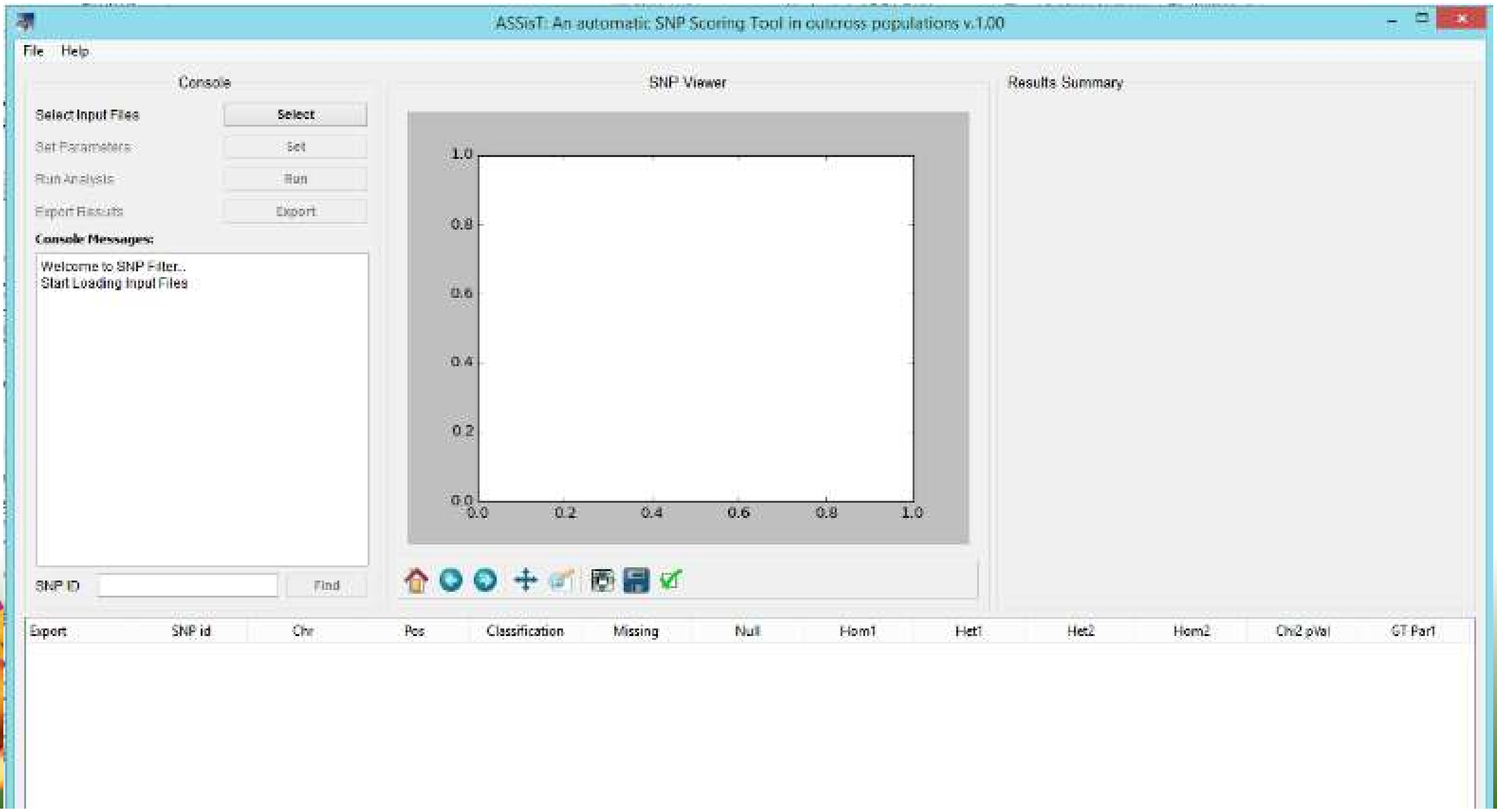

Running ASSIsT

2. In console section, click on 'Select' next to 'Select Input Files'

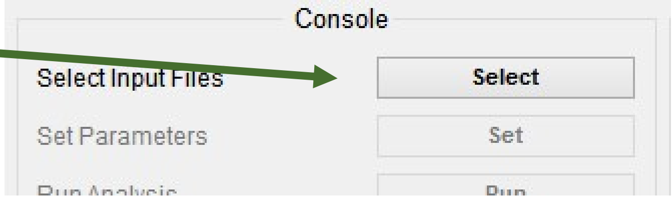

3. Click on '…' boxes to choose each file created earlier. Once each file is chosen, click 'OK'.

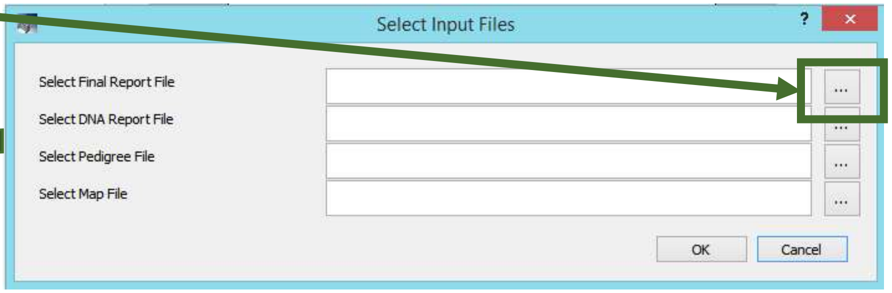

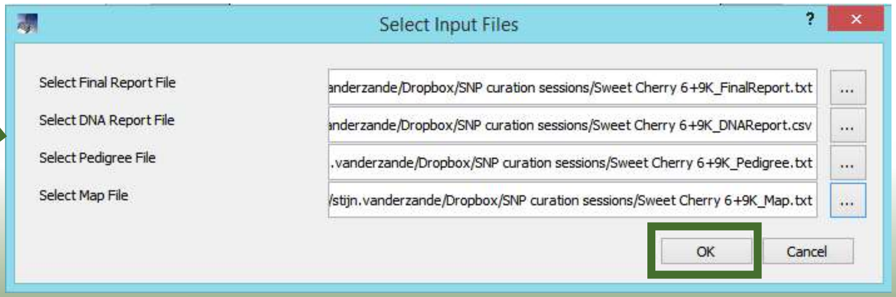

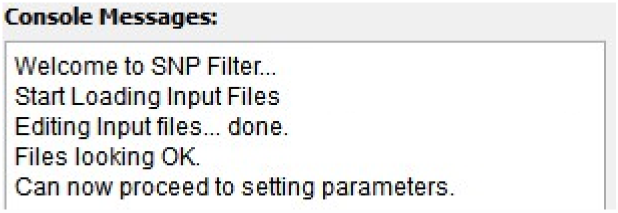

Running ASSIsT

4. In console section, click on 'Set' next to 'Set Parameters'

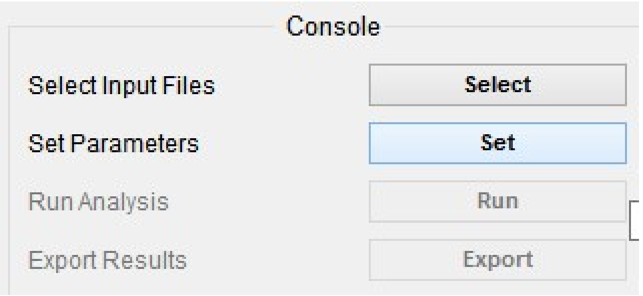

5. Set Parameters as follows:

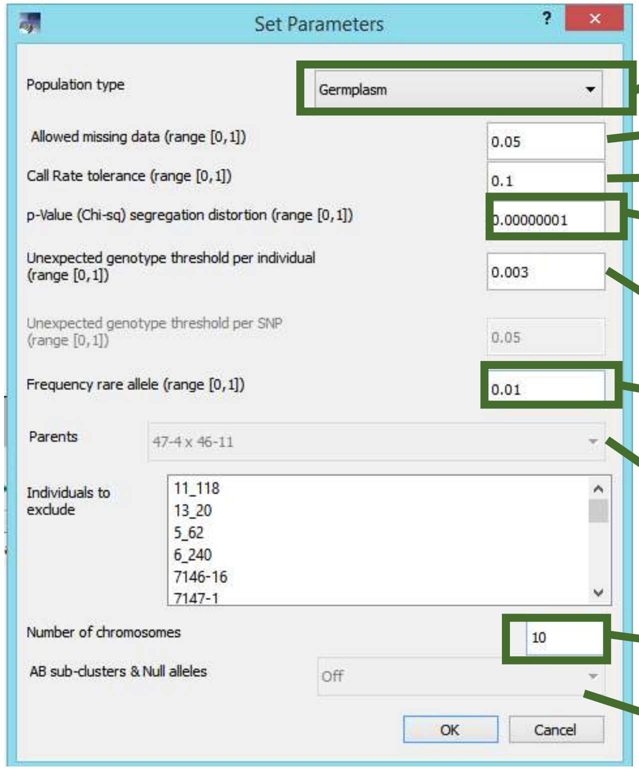

-CP (F1) for (large) F1 population
-Germplasm in most other cases for us

Proportion of allowed missing data

How much can an individual's call rate differ from population mean

How much segregation distortion is allowed?
*lower means more distortion is allowed

Proportion of allowed inconsistencies between child and parents per SNP

Maximum frequency to define an allele as rare
*Only when Germplasm is chosen

Parents (CP (F1), BCx) or grandparents (F2) of the analyzed experimental population.

Chromosome number
*Take "extra" chromosomes into account

Check for Null alleles and AB sub-clusters
*Only for CP (F1) and BC

Running ASSIsT

6. Run the analysis

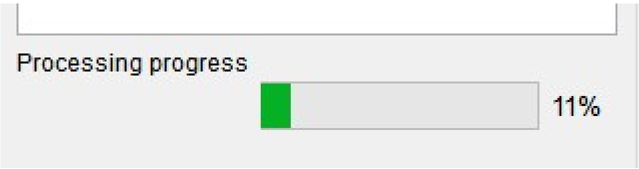

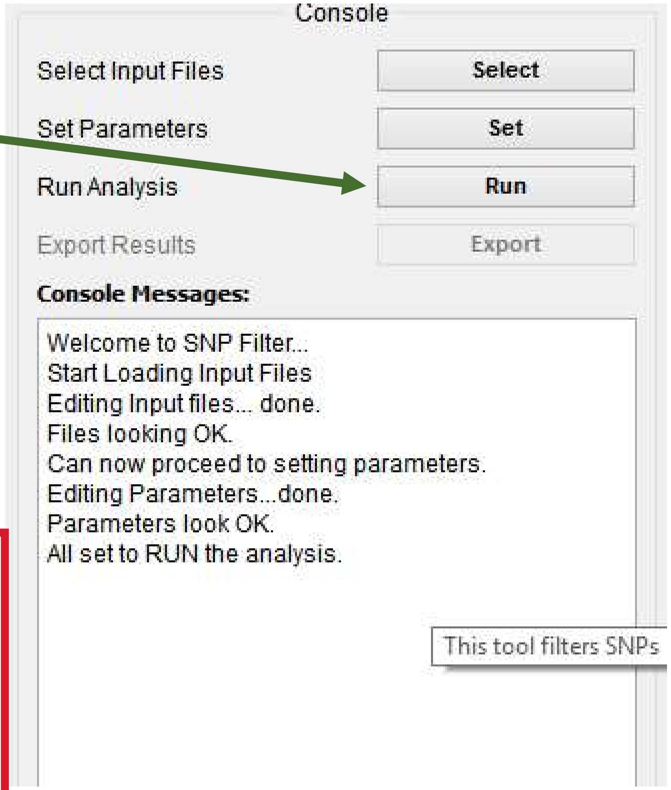

<u>Note:</u>
ASSIsT may stop responding when switching to other programs during the analysis. ASSIsT is still working and after a few minutes, it will show the results of the analysis

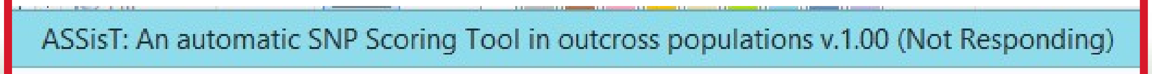

RosBREED
DISEASE RESISTANCE × HORTICULTURAL QUALITY

Results ASSIsT

SNP Graph similar to GenomeStudio
*non-interactive

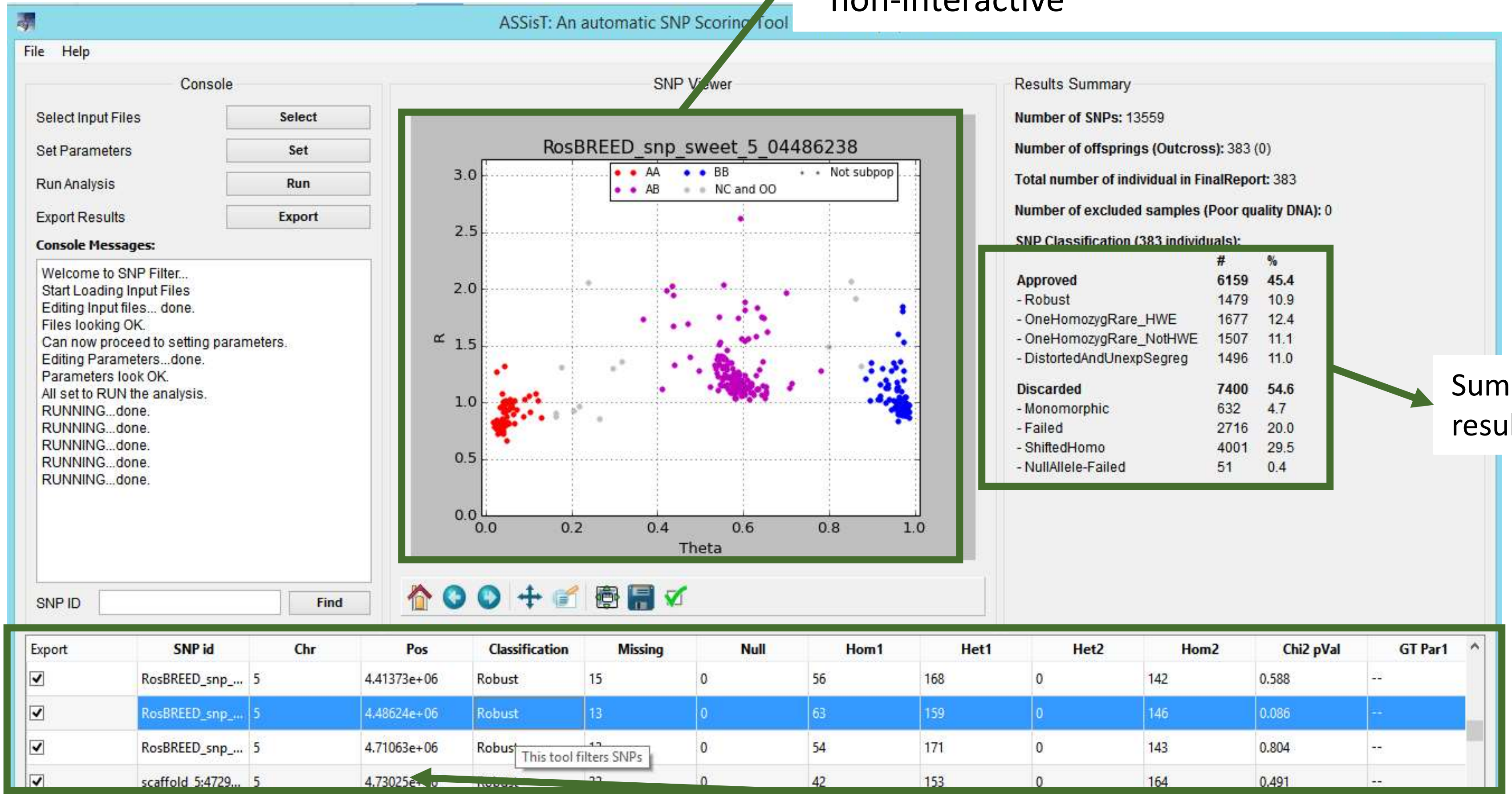

Summary of the results

Results for each SNP
Ordered by Classification, then by Chromosome and Position

Results ASSIsT

Results Summary

Number of SNPs: 13559

Number of offsprings (Outcross): 383 (0)

Total number of individual in FinalReport: 383

Number of excluded samples (Poor quality DNA): 0 → # of excluded samples

SNP Classification (383 individuals):

	#	%
**Approved**	**6159**	**45.4**
- Robust	1479	10.9
- OneHomozygRare_HWE	1677	12.4
- OneHomozygRare_NotHWE	1507	11.1
- DistortedAndUnexpSegreg	1496	11.0
**Discarded**	**7400**	**54.6**
- Monomorphic	632	4.7
- Failed	2716	20.0
- ShiftedHomo	4001	29.5
- NullAllele-Failed	51	0.4

Approved → SNPs used for further curation

Discarded → Discarded SNPs*

*Note:
Shifted-Homo SNPs were added back in but many required manual adjustment of clustering

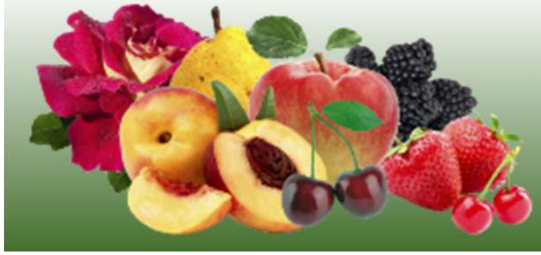

RosBREED
DISEASE RESISTANCE × HORTICULTURAL QUALITY

Export Results

1. Click on "Export" button in console section

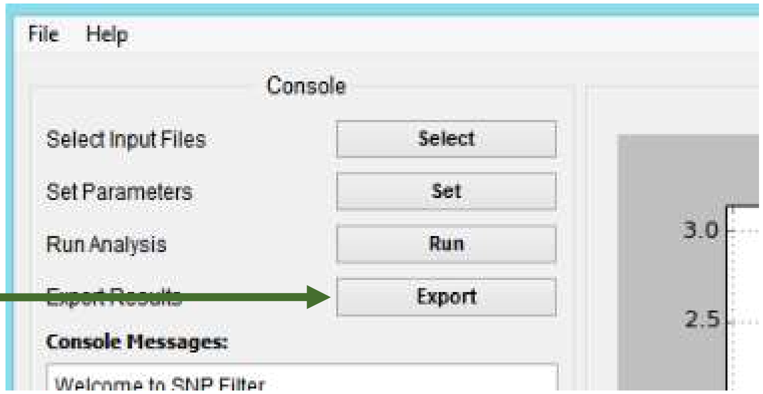

2.-Choose the files you want (next slide)
-Choose folder to save files in
-Choose name for files
*Avoid spaces in file names

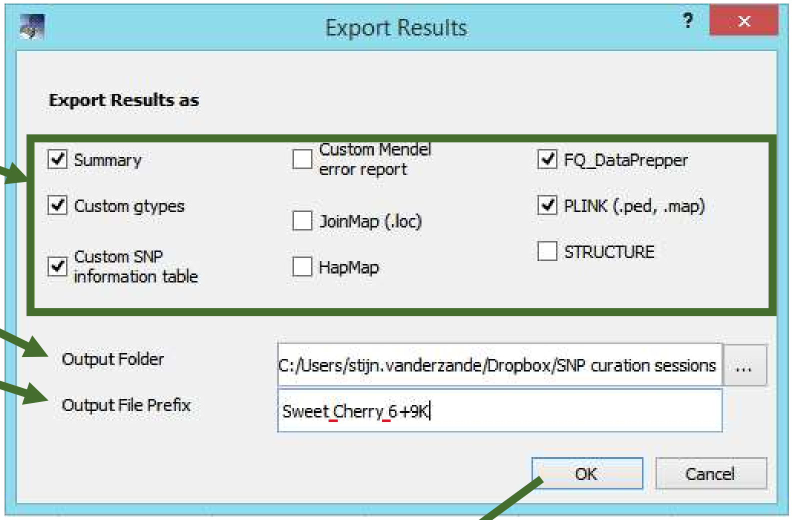

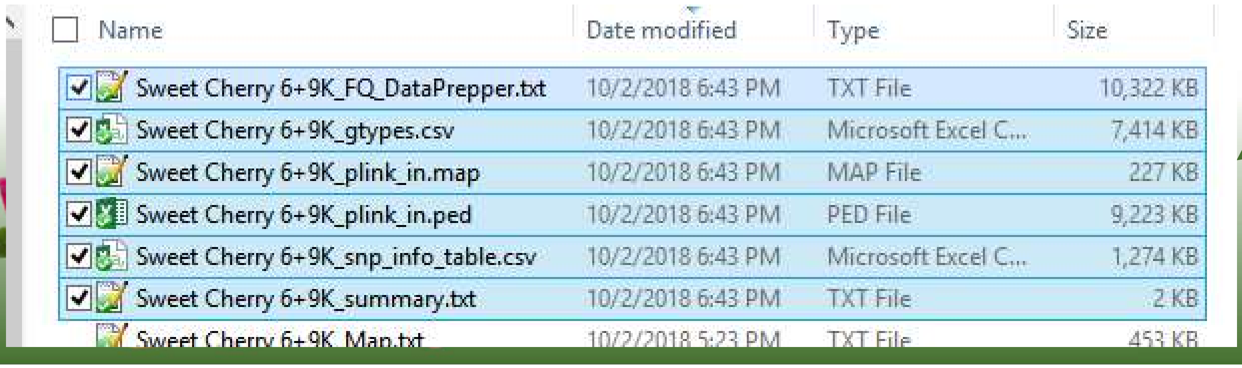

Export Results

Approved	6159	45.4
Robust	1479	10.9
OneHomozygRare_HWE	1677	12.4
OneHomozygRare_NotHWE	1507	11.1
DistortedAndUnexpSegreg	1496	11.0
Discarded	7400	54.6
Monomorphic	632	4.7
Failed	2716	20.0
ShiftedHomo	4001	29.5
NullAllele-Failed	51	0.4

☑ Summary

Summarizes Parameter settings and Results (as shown on the right in ASSIsT)

Parameter Set
Population type: Germplasm
Allowed missing data: 0.1
Call Rate tolerance: 0.1
p-Value (Chi-sq) segregation distortion: 1e-08
Unexpected genotype threshold: 0.009
Frequency rare allele: 0.01
Number of chromosomes: 10
AB sub-clusters & Null alleles: Off

☑ Custom gtypes

Gives genotype for each individual and each **approved** SNP

	A	B	C	D	E	F	G	H	I	J	K	L	M	N	O	
1	SNP id	Chr	Pos	Classificat	Missing	Null	Hom1	Het1	Het2	Hom2	Chi-Squar	1_118	13_20	5_62	6_240	714
2	RosBREE	1	43011	Distorted	25	0	14	265	0	77	0			AG	AG	AG
3	scaffold_	1	43481	OneHomc	15	0	2	288	0	76	0			AC	AC	AC
4	RosBREE	1	51332	OneHomc	13	0	357	9	0	2	0			AA	AA	AA
5	S1_5444	1	54446	OneHomc	4	0	358	18	0	1	0			AA	AA	AA
6	scaffold_	1	56261	OneHomc	12	0	1	8	0	360	0			GG	GG	GG
7	RosBREE	1	94685	OneHomc	11	0	356	12	0	2	0			AA	AA	AA

SNPs are ordered according to their position

Genotype score and individuals missaligned?!

Genotypes calls

☑ Custom SNP information table

Gives results of analysis for each SNP (as shown on the bottom in ASSIsT

☑ PLINK (.ped, .map)

Two files (.ped and .map) needed to run PLINK and identify unknown duplicates (see further)

☑ FQ_DataPrepper

Helps create FlexQTL files needed for further data curation (see next sessions)

United States Department of Agriculture

National Institute of Food and Agriculture

duplicates

RosBREED

DISEASE RESISTANCE × HORTICULTURAL QUALITY → SUPERIOR CULTIVARS

Known duplicates

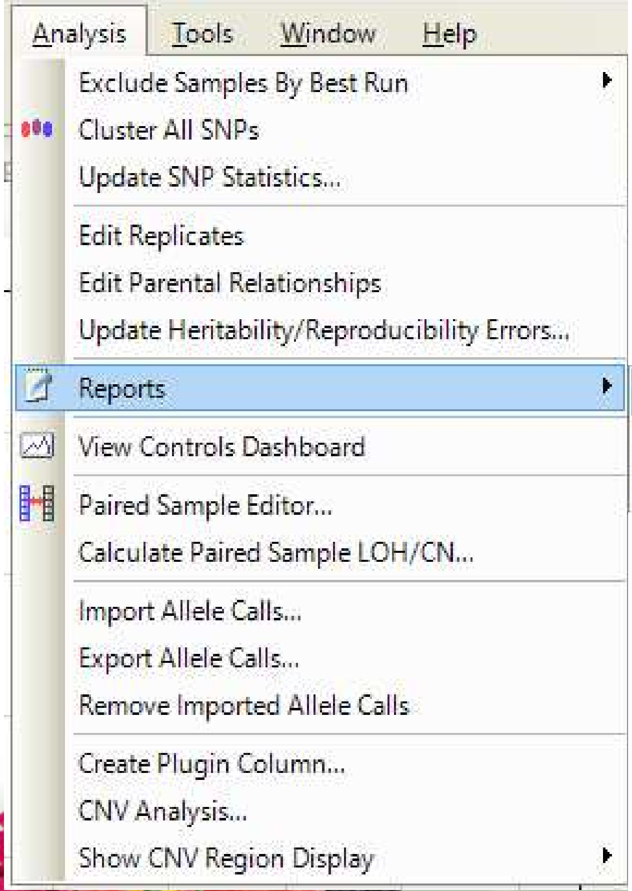

1. Create Reproducibility and Heritability report in GenomeStudio

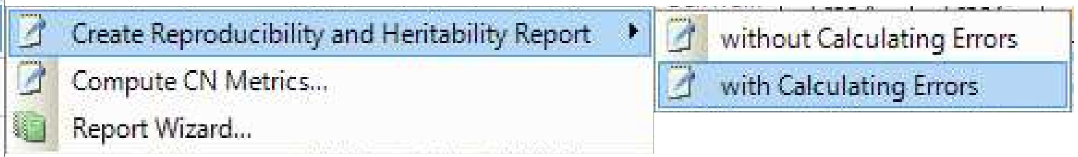

2. Choose name and save file

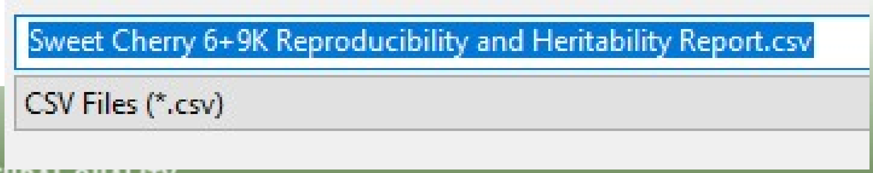

RosBREED
DISEASE RESISTANCE × HORTICULTURAL QUALITY

Known duplicates

1. Open Reproducibility and Heritability report
   Duplicate comparison found under 'Duplicate Reproducibility'

Duplicate Reproducibility

Rep1_DNA_Name	Rep2_DNA_Name	# Correct	# Errors	Total	Repro_Freq
Bing	Bing_rep01	8829	647	9476	0.965258
Bing_rep01	Bing	8829	647	9476	0.965258
FR070T105	FR070T105_rep01	11139	2	11141	0.99991
FR070T105_rep01	FR070T105	11139	2	11141	0.99991
FR072T074	FR072T074_rep01	11150	0	11150	1
FR072T074_rep01	FR072T074	11150	0	11150	1

Name Individual

Name Duplicate

Number consistent genotype calls

Number inconsistent genotype calls

Proportion inconsistent genotype calls

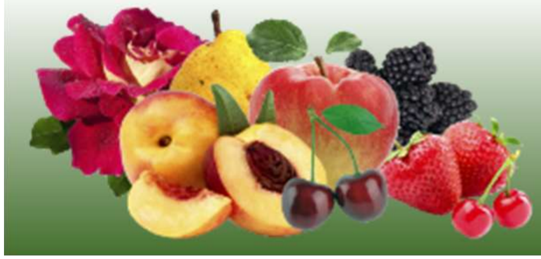

Finding unknown duplicates

1. Copy .ped and .map file into PLINK folder
   ASSIsT output

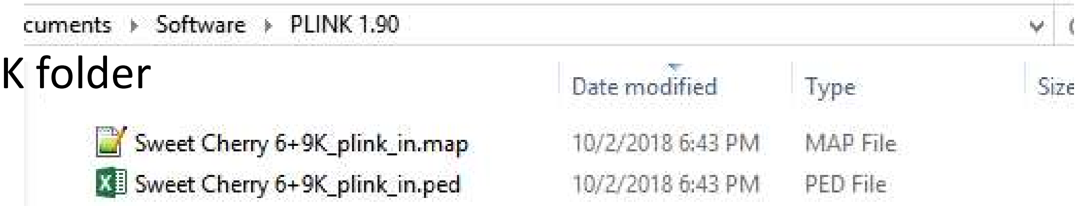

2. Hold ‘shift’ and right click in the PLINK folder to get
   a. the “Open command window here” option
   b. the “Open PowerShell window here” option

a

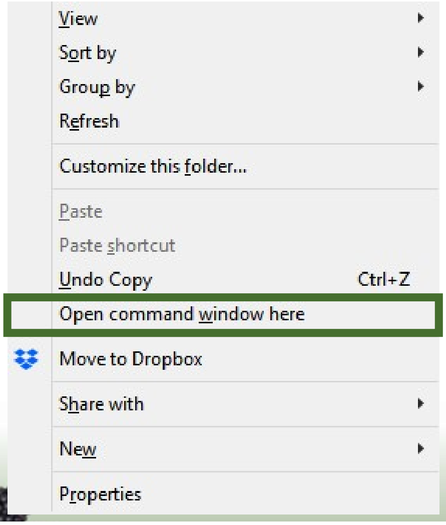

b

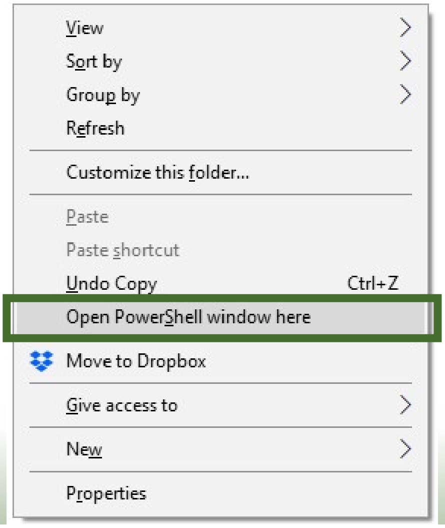

3. (left) click on “Open command window here”/”Open PowerShell window here” to open the respective window

Using Plink – a. command window

4. Start with “plink.exe” then add additional commands with space between each. Press enter when all commands are given

*change “filename” to name of .ped and .map file

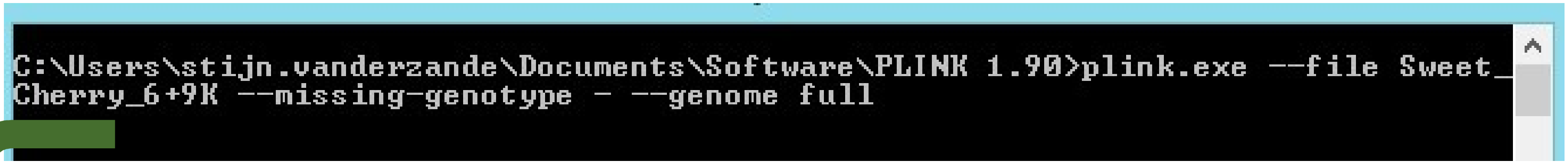

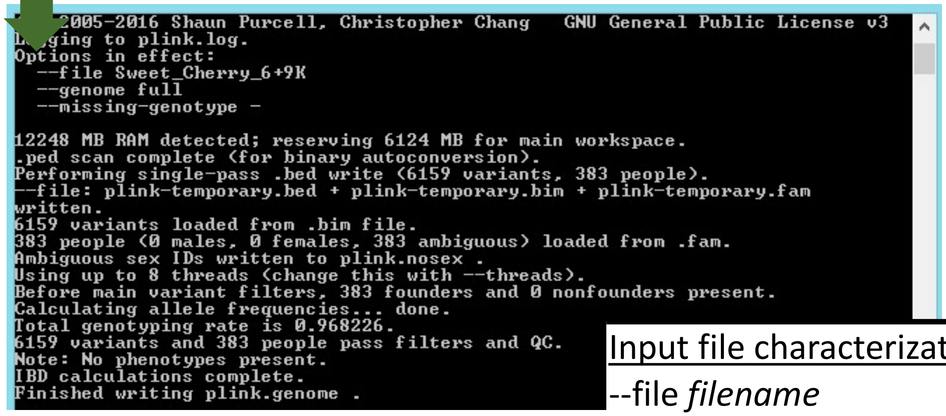

Expects same name for .ped and .map file

*e.g. Sweet_Cherry_6+9K.ped and Sweet_Cherry_6+9K.map

*NO blank spaces in name

Input file characterization	
--file *filename*	Define files, change *filename* to name of ped and map file
--no-fid	when FID column is missing
--no-sex	when sex column is missing
--allow-no-sex	when sex column is missing, needed for some analyses
--no-pheno	when phenotype column is missing
--missing-genotype *N*	Define missing genotype (*N*) when different from 0
Analysis	
--genome full	calculate IBD (IBS) for all individuals

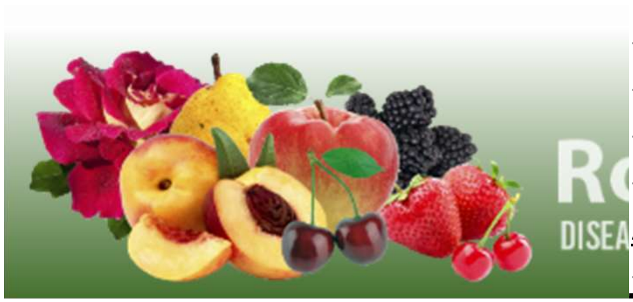

Using Plink – b. PowerShell window

4. Start with ".\plink.exe" then add additional commands with space between each. Press enter when all commands are given

*change "filename" to name of .ped and .map file

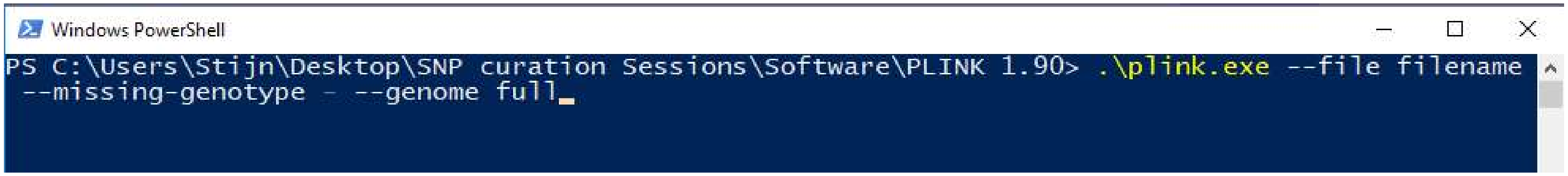

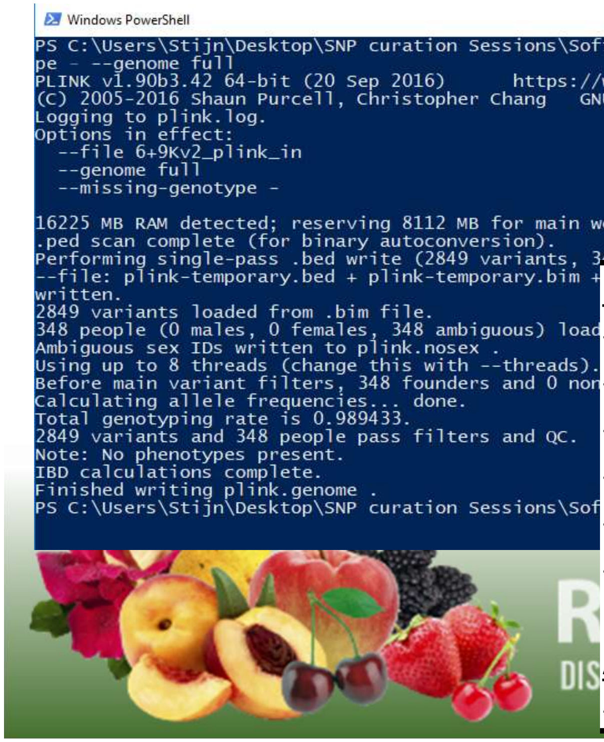

Expects same name for .ped and .map file

*e.g. Sweet_Cherry_6+9K.ped and Sweet_Cherry_6+9K.map

*NO blank spaces in name

Input file characterization	
--file *filename*	Define files, change *filename* to name of ped and map file
--no-fid	when FID column is missing
--no-sex	when sex column is missing
--allow-no-sex	when sex column is missing, needed for some analyses
--no-pheno	when phenotype column is missing
--missing-genotype *N*	Define missing genotype (*N*) when different from 0
Analysis	
--genome full	calculate IBD (IBS) for all individuals

Using Plink

5. Open plink.genome with Excel

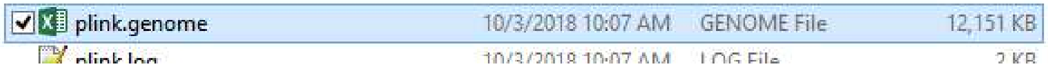

6. Select first column, use “Text to Columns” to create separate columns

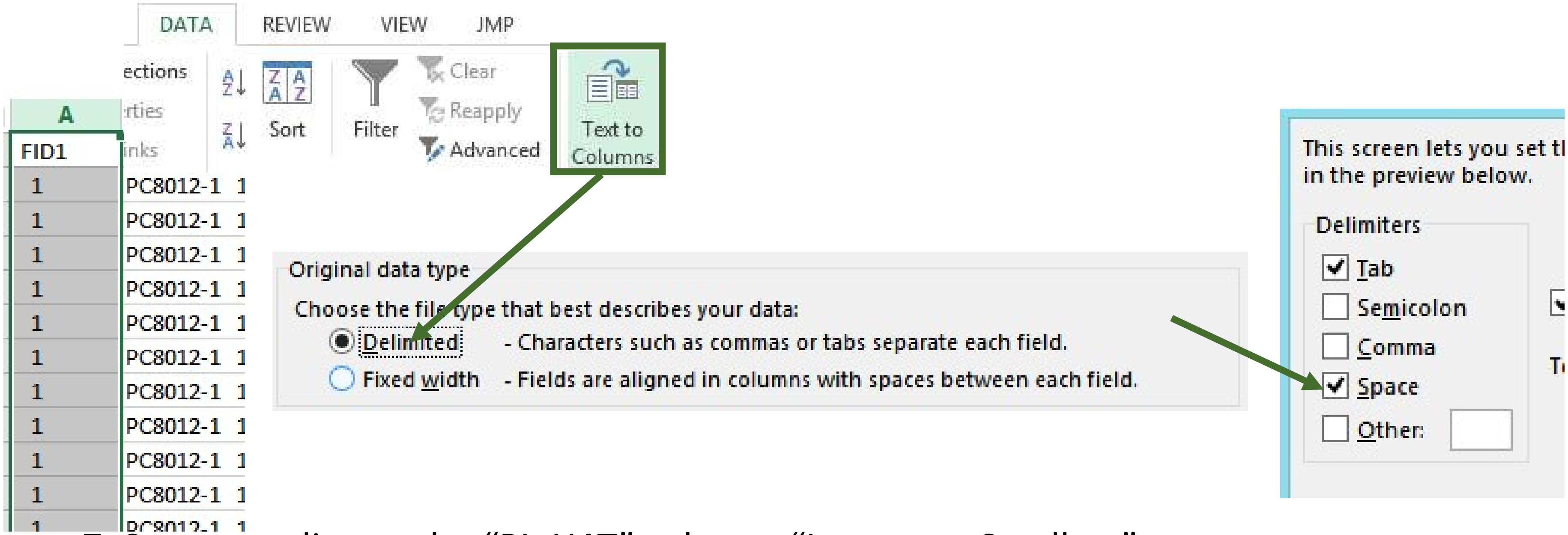

7. Sort according to the “PI_HAT” column, “Largest to Smallest”

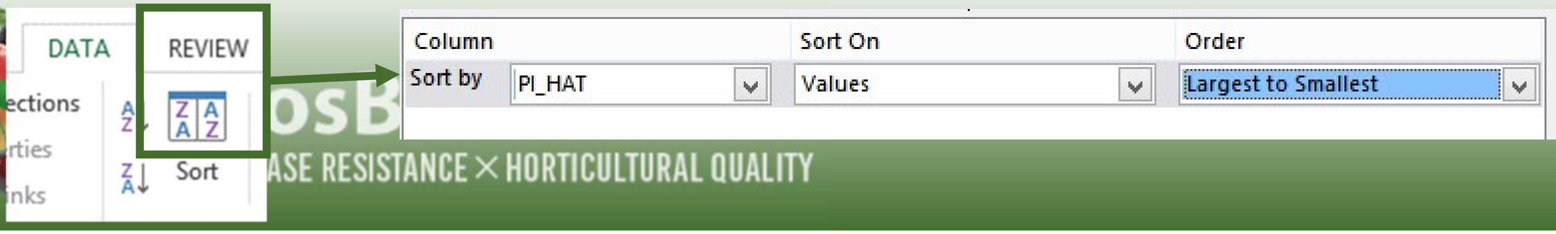

Plink Results

Individual 1 — Individual 2 — IBS

FID1	IID1	FID2	IID2	PI_HAT	HE	DST	PPC	RATIO	IBS0	IBS1	IBS2	HOMHO	HETHET
	MBigarreau_dup02		MBigarreau_dup01	1	-1	1	1	NA	0	0	2805	0	232
	Lambert		KootenayLambert	1	-1	1	1	NA	0	0	2849	0	218
	99F/132R4		99Fl131_RJ	1	-1	1	1	NA	0	0	2849	0	260
	MertonBigarreau		MBigarreau_dup02	0.9995	-1	0.999824	1	NA	0	1	2842	0	234
	MertonBigarreau		MBigarreau_dup01	0.9995	-1	0.999822	1	NA	0	1	2806	0	232
	Skeena		Santina_dup01	0.9985	-1	0.999473	1	NA	0	3	2842	0	197
	Santina_dup01		Santina	0.9985	-1	0.999473	1	NA	0	3	2842	0	197
	MertonHeart		MertonHeart_dup01	0.9984	-1	0.999422	1	NA	0	3	2590	0	249
	Hedelfingen		Hedelfingendup01	0.998	-1	0.999295	1	NA	0	4	2833	0	261
	StarBlush		StarBlush_dup01	0.997	-1	0.99894	1	NA	0	6	2824	0	231
	SandraRose_dup01		SandraRose	0.9892	-1	0.99618	1	NA	0	21	2728	0	222
	Vega		Cv1	0.976	-1	0.991929	1	NA	1	40	2561	0	213
	FR063T023		FR055T064	0.8279	-1	0.939648	1	97	1	300	2201	1	97

0.97 as cutoff for duplicates

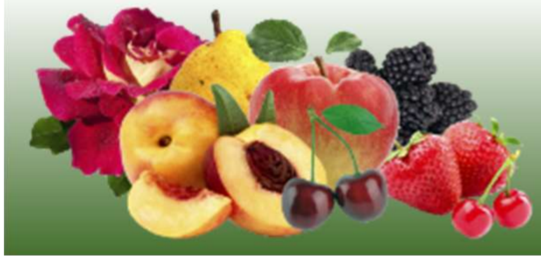

RosBREED
DISEASE RESISTANCE × HORTICULTURAL QUALITY

Duplicate triploids

- Calls should be the same between duplicates
  - Are AB calls identical?
    - Could be AAB or ABB
    - B allele frequency
      - Should both be 0.33 or 0.66
      - Correlation between identically called individuals

	IID1	FI	IID2	PI_HAT
1	"PommeHervi"	1	"StreepingAlken"	1
1	"ZoeteAagt"	1	"BelledeFumes"	1

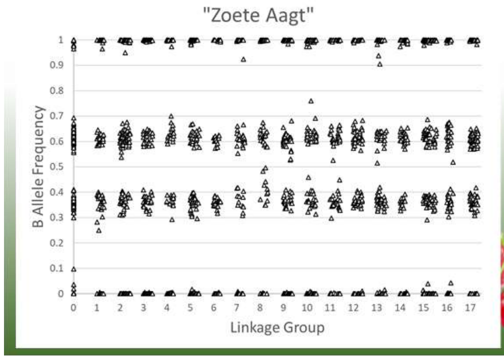

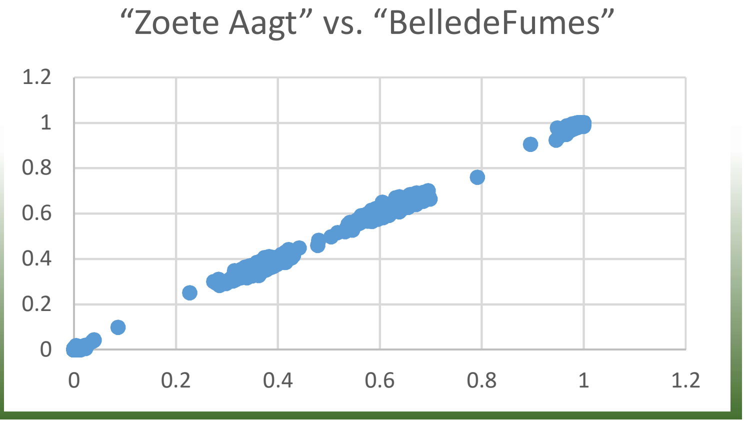

United States Department of Agriculture

National Institute of Food and Agriculture

Pedigree check

RosBREED

DISEASE RESISTANCE × HORTICULTURAL QUALITY → SUPERIOR CULTIVARS

Checking parent-child relationships

1. Open ‘Reproducibility and Heritability’ report created earlier for known duplicates
   A. Parent-child errors found under ‘P-C Heritability’

Only when parent in dataset

P-C Heritability					
Child_DNA_Name	Parent_DNA_Name	# Correct	# Errors	Total	P-C Heritability Freq
Abundance	Napoleon	0	0	0	N/A
Burbank	EarlyPGuigne	0	0	0	N/A
Coe	OxHeart	0	0	0	N/A
Summit	Van	0	0	0	N/A
Kordia	Schneiders	11593	2	11595	0.9998275
Sunburst	Summit	11601	8	11609	0.9993109

Name child

Name Parent

Number genotype calls without PC error

Number genotype calls with PC error

Proportion genotype calls with PC error

RosBREED
DISEASE RESISTANCE × HORTICULTURAL QUALITY

Checking parent-parent-child relationships

1. Open ‘Reproducibility and Heritability’ report created earlier for known duplicates

   B. Parent-child errors found under ‘P-P-C Heritability’

P-P-C Heritability

Child_DNA_Name	Parent1_DNA_Name	Parent2_DNA_Name	# Correct	# Errors	Total	P-P-C Heritability Freq
Chelan	Stella	Moreau	11554	12	11566	0.999
Index	Stella	Bing	11551	12	11563	0.999
Benton	Stella	Moreau	11541	15	11556	0.9987
Vic	Bing	Schmidt	11542	20	11562	0.9983
Venus	Hedelfingen	Windsor	11475	31	11506	0.9973
Santina	Stella	Summit	11205	339	11544	0.9706
Santina_dup01	Stella	Summit	11102	350	11452	0.9694

Name child

Name Parent 1

Name Parent 2

Number genotype calls without PC error

Number genotype calls with PPC error

Proportion genotype calls with PPC error

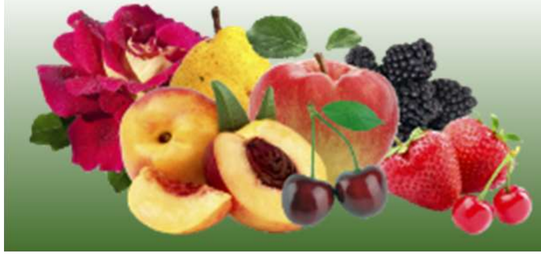

Finding new P(P)C relationships

1. Open ASSIsT's ".gtypes" output in Excel

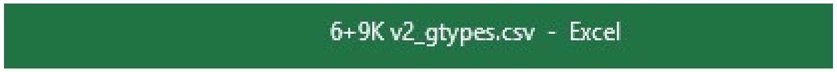

2. Ensure genotypic data aligns with individuals' ID

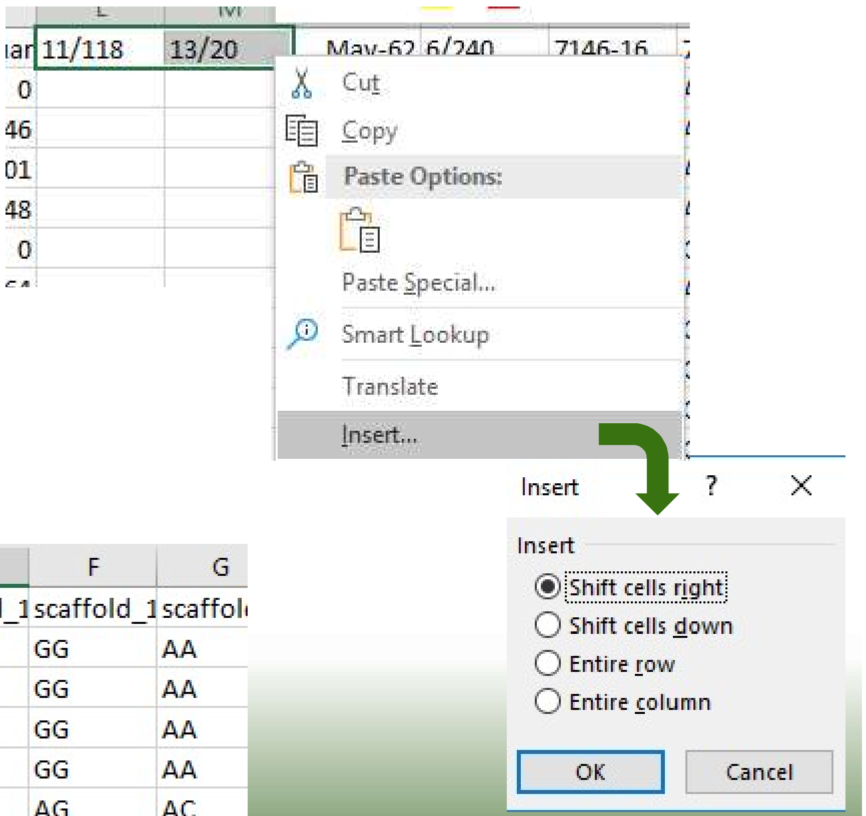

3. Copy and transpose data so that individuals are in rows and markers in columns

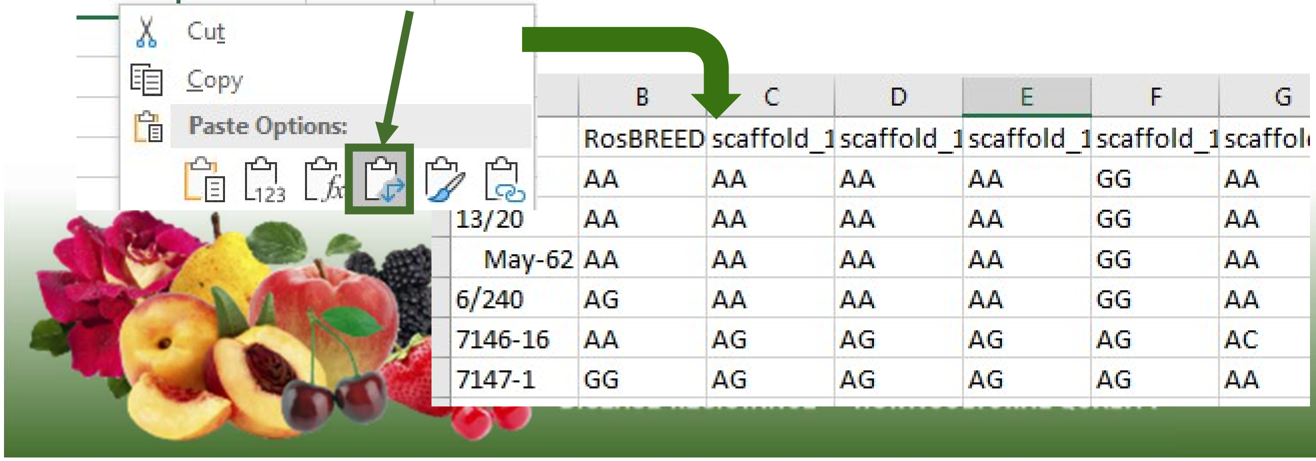

Finding new P(P)C relationships

4. Bring in pedigree information for all individuals (parents do not have to be included in data set)

5. Set missing parents to “-”

6. Use “Ind”, “Parent1” and “Parent2” as column headers

Ind	Parent1	Parent2	RosB[illegible]
11/118			AA
13/20			AA

7. Save file as “.csv” in same folder as “Parent Check” R script (Suppl. Document 2)

Finding new P(P)C relationships

8. Load libraries in R

```
library(stringr)
library(svMisc)
```

9. Define functions in R by running them

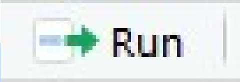


```
##Functions
AdjustMV <- function(GTData){…}

CheckParErr <- function(GenotypeIndPar){…}

CheckParParErr <- function(GenotypeIndParPar){…}

CheckPar <- function(GenotypesIndParPar){…}

FindParGT <- function(IndName, Genotypes){…}

CheckParAll <- function(IndToCheck, ChrReport=NULL){…}

FindPosPar <- function(IndName, GTData, treshold){…}

FindPosParComb <- function(GTData, tresholdPE=0, tresholdPPE=0){…}
```

10. Set working directory to source file location

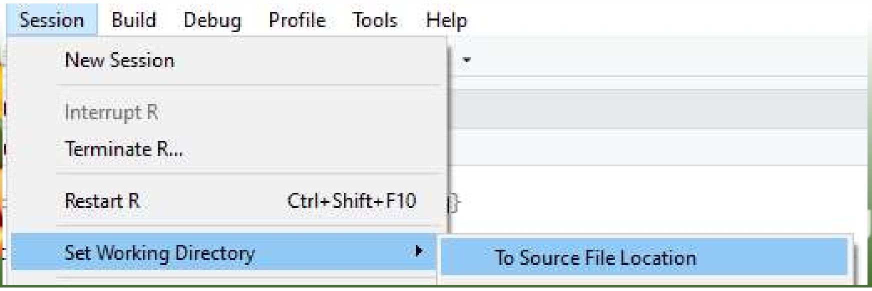

Finding new P(P)C relationships

11. Define:

- Possible Alleles under AlleleList
  - $ or $$ does not work for null alleles
- Characters used for missing genotypes under “MissGT”
- Characters used for missing alleleles under “MissAllele”

```
AlleleList <- c("A","B","C","null", "G", "T")
MissGT <- c("NC","00", "--")
MissAllele <-c("N","0","-")
```

12. Provide filename and load into R

```
## Load data files
GenotypeData <- as.data.frame(read.csv(file="Sweet_Cherry_6+9K.csv", head=T, sep=",", stringsAsFactors=F))
```

13. Run “FindPosParComb”

Loaded data file

Threshold to accept PC relationship

Threshold to accept PPC relationship

```
> FindPosParComb(GenotypeData, tresholdPE = 70, tresholdPPE = 100)
[1] "Starting step 1 of 3"
[1] "Starting step 2 of 3"
Progress:  1 on 40
```

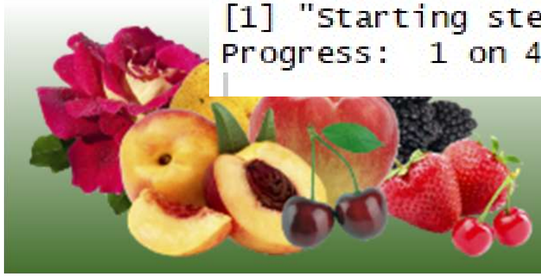

3 steps:
-Identify possible mothers for ind. with missing mothers
-Identify possible fathers for ind. with missing fathers
-Identify parents for ind. with both parents missing

Checking and finding grandparent-grandchild relationships

- Excel - Suppl file 1 from van de Weg et al. (2018)
  - If offspring is ‘AB’ and
    - 1 known parent is ‘AA’ OR
    - Two known grandparents through single parent are both ‘AA’
  - Then: one unknown grandparent cannot be ‘AA’
    - Minimize ‘AA’ calls gives indication of possible grandparent
    - For any ‘AA’ in putative grandparent, second unknown grandparent cannot be ‘AA’
  - Also true for ‘AB’ in individual and ‘BB’ in known parents/grandparents

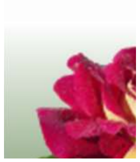

van de Weg E, Di Guardo M, Jansch M, Socquet-Juglard D, Costa F, Baumgartner I, Broggini GAL, Kellerhals M, Troggio M, Laurems F, Durel CE, Pattochi A. (2012) Epistatic fire blight resistance QTL alleles in the apple ‘Enterprise’ and selection X-6398 discovered and characterized through pedigree-informed analysis. Molecular Breeding 38:5

van de Weg et al. (2018) method

1. Filter individual for AB
2. Filter parent 1 for AA
3. Parent 2 should have 0 AA

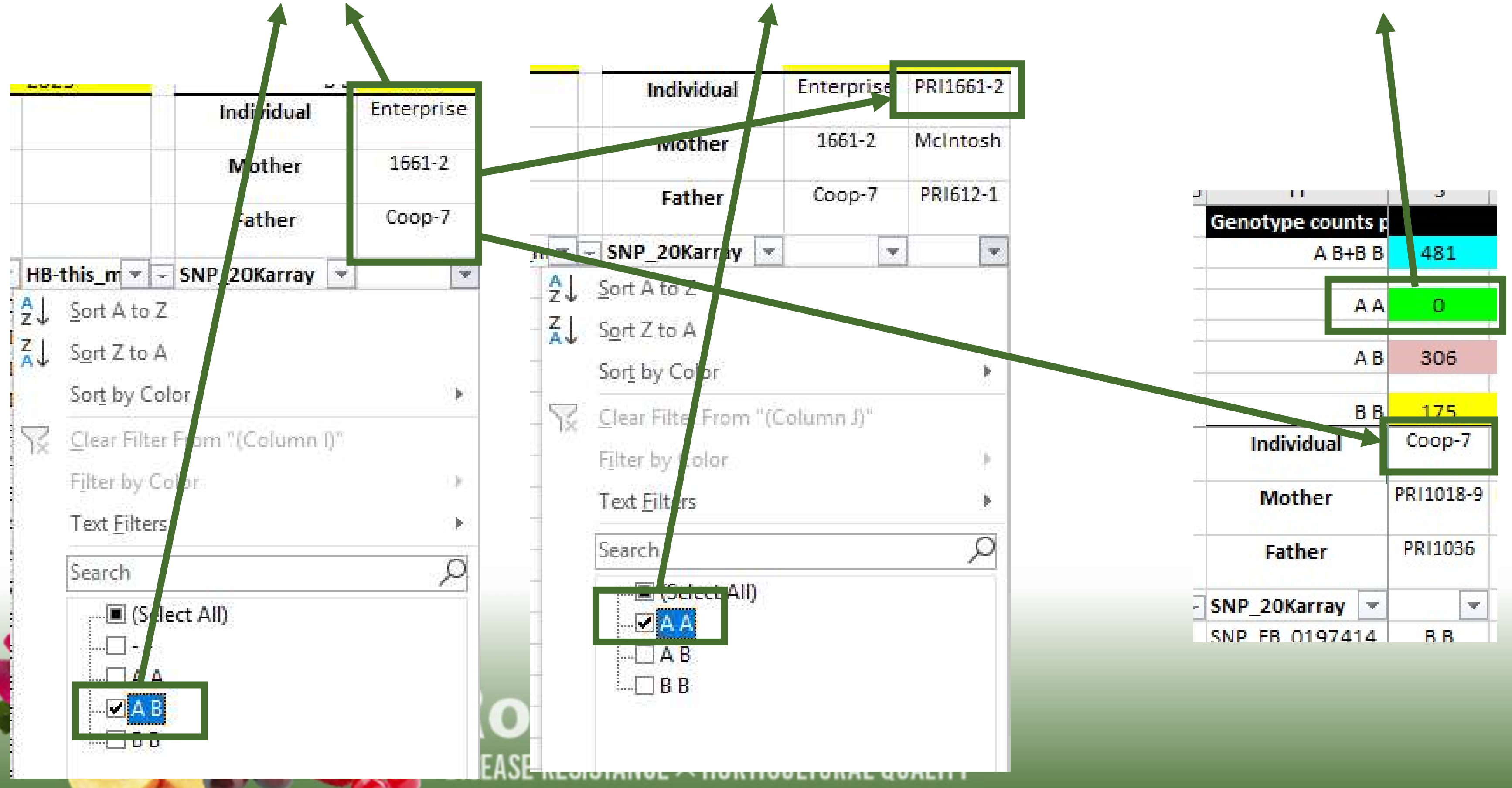

United States Department of Agriculture

National Institute of Food and Agriculture

Curating remaining Mendelian-inconsistent errors

RosBREED

DISEASE RESISTANCE × HORTICULTURAL QUALITY → SUPERIOR CULTIVARS

USDA

United States Department of Agriculture

National Institute of Food and Agriculture

FlexQTL Data Prepper

RosBREED

DISEASE RESISTANCE × HORTICULTURAL QUALITY → SUPERIOR CULTIVARS

FlexQTL Data Prepper

- FlexQTL Data Prepper
  - Requires 3 input files
    - Marker file
      - Adjusted ASSIsT output file
    - Pedigree file
      - Adjusted ASSIsT input file
    - Data file
      - Generated by ASSIsT

RosBREED
DISEASE RESISTANCE × HORTICULTURAL QUALITY

FlexQTL Data Prepper

Creating 1st input file (Marker file)

1. Create a three-column file:
   - -1st column has the SNP's name/SNP's ID
   - -2nd column has the chromosome (numerical)
   - -3rd column has the genetic position (cM))
   - -Header row should be "MarkerId", "Group", "Position"
   - -Save as ".csv" file

*Start from "…_plink_in.map" ASSIsT output file

- *Change header row
- *Change physical position to genetic position if needed

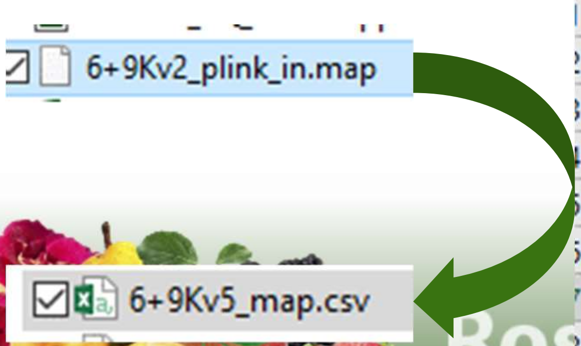

A	B	C
MarkerId	Group	Position
RosBREED_snp_tart_1_0021	1	0
scaffold_1:425480	1	3.62
scaffold_1:427005	1	3.64
scaffold_1:427260	1	3.65
scaffold_1:429080	1	3.68
scaffold_1:433354	1	3.75
RosCOS1201-071_snp_swee	1	4.04
scaffold_1:459954	1	4.2

FlexQTL Data Prepper

Creating 2nd input file (Pedigree file)

2. Create a three-column file:
    - -1st column has the individual's name
    - -2nd column has the female parent
    - -3rd column has the male parent
    - -Header row should be "Name", "Parent1", "Parent2"
    - -Save as ".csv" file

*Start from ASSIsT pedigree input file

*Change header row

Name	Parent1	Parent2
Abundance	Napoleon	
BlackRepublican	Napoleon	BlackTartarian
EmperorFrancis		
Gil-Peck	Napoleon	Giant
Hedelfingen		
Kordia	Schneiders	
Lambert	Napoleon	Blackheart
Summit	Van	
Sunburst	Summit	
Van	EmpressEugenie	BlackRepublican

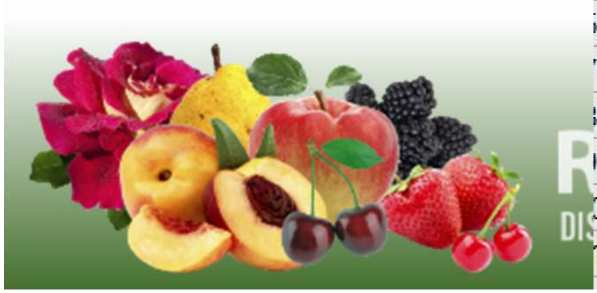

FlexQTL Data Prepper

<u>Creating 3rd input file (Data file)</u>

3. Adjust "…_FQ_DataPrepper.txt" file format from ".txt" to ".csv"

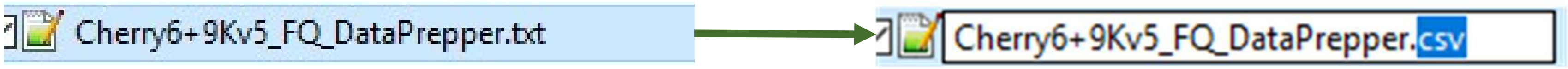

<u>Note:</u>

If file format extension is not visible, enable it under the "View Tab" in the file's folder

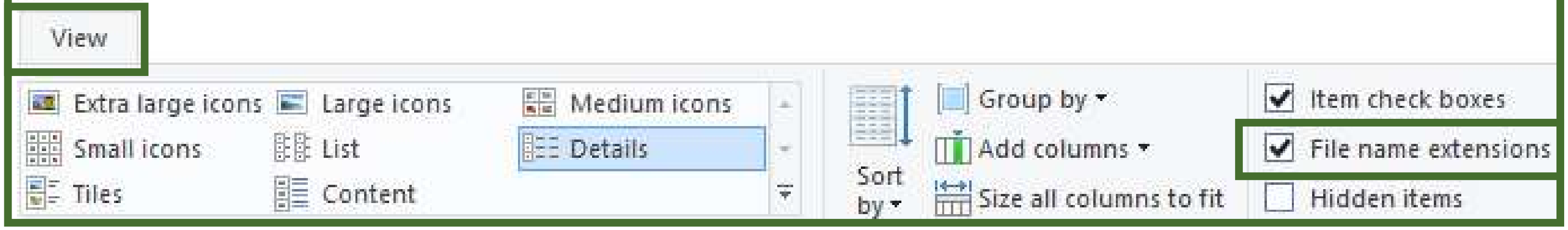

4. Open "…_FQ_DataPrepper.txt" file in Excel

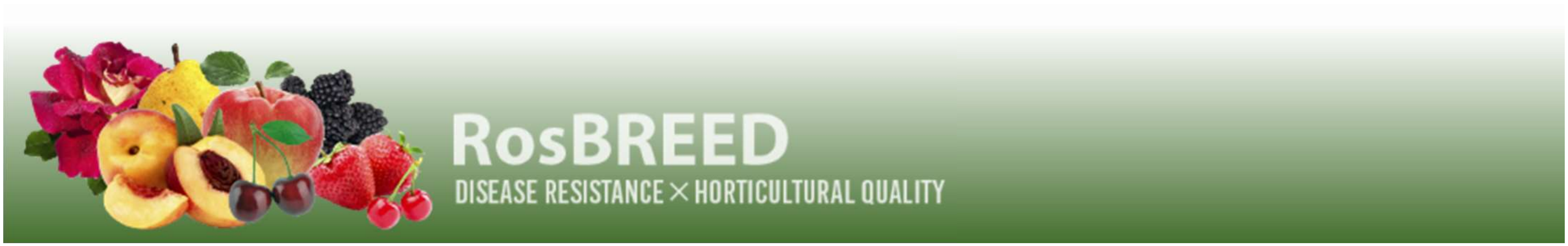

FlexQTL Data Prepper

5. Adjust header row so it matches genotypic data

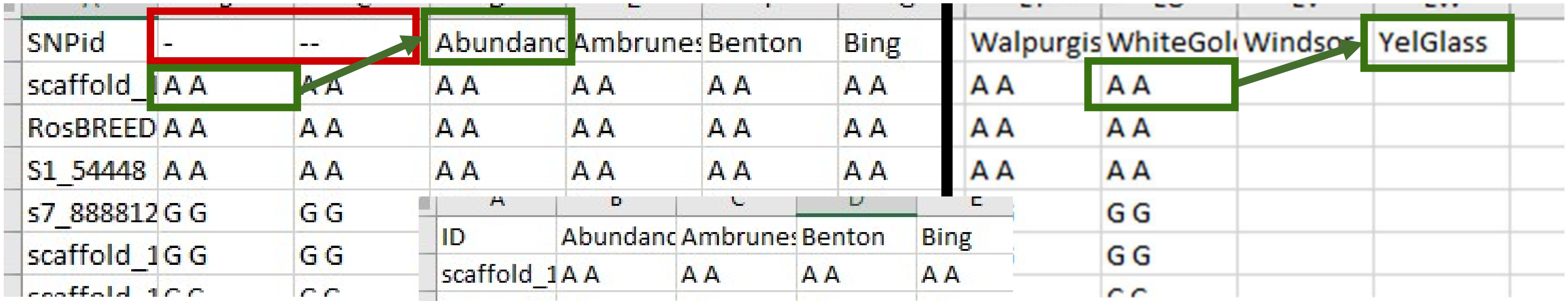

A	B	C	D	E	F	G	…	EW	EX	EY	EZ
SNPid	-	--	Abundanc	Ambrunes	Benton	Bing		Walpurgis	WhiteGol	Windsor	YelGlass
scaffold_1	A A	A A	A A	A A	A A	A A		A A	A A		
RosBREED	A A	A A	A A	A A	A A	A A		A A	A A		
S1_54448	A A	A A	A A	A A	A A	A A		A A	A A		
s7_888812	G G	G G							G G		
scaffold_1	G G	G G							G G		

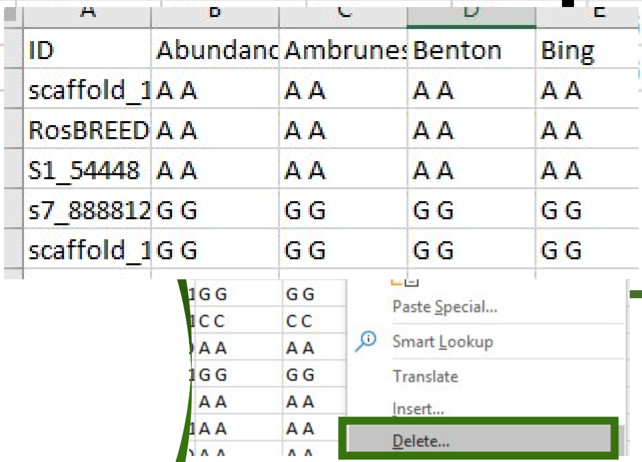

A	B	C	D	E
ID	Abundanc	Ambrunes	Benton	Bing
scaffold_1	A A	A A	A A	A A
RosBREED	A A	A A	A A	A A
S1_54448	A A	A A	A A	A A
s7_888812	G G	G G	G G	G G
scaffold_1	G G	G G	G G	G G

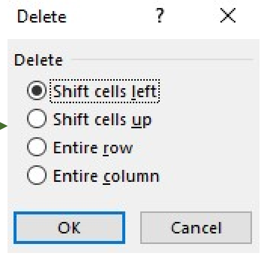

6. Change cell A1 to "ID"

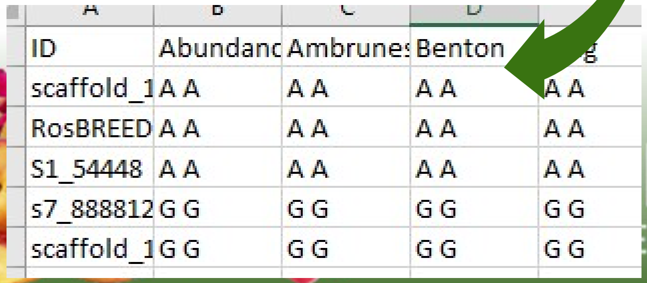

A	B	C	D	E
ID	Abundanc	Ambrunes	Benton	[illegible]
scaffold_1	A A	A A	A A	A A
RosBREED	A A	A A	A A	A A
S1_54448	A A	A A	A A	A A
s7_888812	G G	G G	G G	G G
scaffold_1	G G	G G	G G	G G

7. Save file

FlexQTL Data Prepper

8. Open FlexQTLDataPrepper.exe

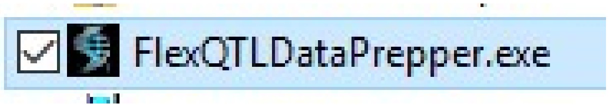

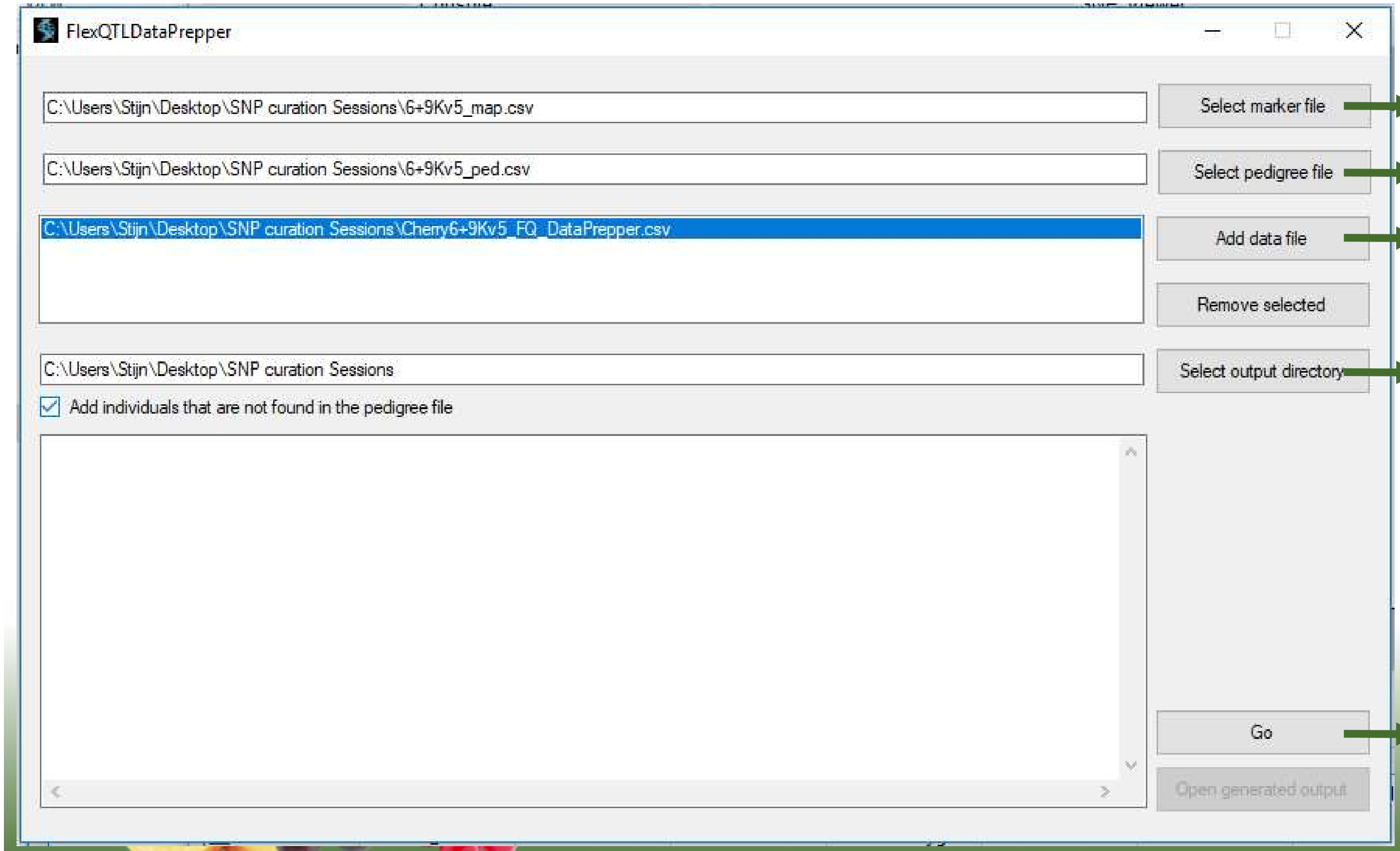

9. Load marker file

10. Load pedigree file

11. Load data file

12. Choose output directory

13. Select this option

14. Press 'Go'

United States
Department of
Agriculture

National Institute
of Food and
Agriculture

FlexQTL™ Input files

RosBREED

DISEASE RESISTANCE × HORTICULTURAL QUALITY → SUPERIOR CULTIVARS

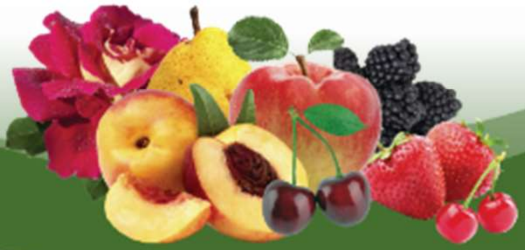

FlexQTL™ input files

- Three files
  - Data file (.dat)
  - Map file (.map)
  - Parameter file (.par)
  - Text-based files
  - Everything after “;” will be ignored
    - Easy excluding of rows in file

FlexQTL™ input files

- Data file (.dat file)
  - No header needed (if added, start with ";")
  - Column 1: Population
  - Column 2: Individual
  - Column 3: Parent 1
  - Column 4: Parent 2
    - Both parents needed (or none)
    - Use dummy individuals when only one parent is available
  - Column 5 – X: nuisance variables
  - Columns X+1 – Y: Phenotypic variables
  - Columns Y+1 – End: Genotypic data
    - Two columns per marker
    - One column per marker with space(s) between alleles

FlexQTL^{TM} input files

- Map file (.map)
  - Column 1: marker name
  - Column 2: genetic position within LG
  - Each LG starts with "group X"
    - X is LG number
  - Identical number of markers needed as in data file

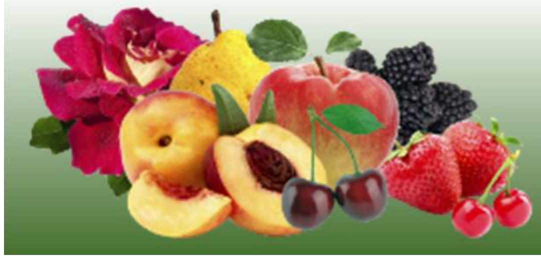

RosBREED
DISEASE RESISTANCE × HORTICULTURAL QUALITY

FlexQTL™ input files

- Parameter file (.par)
  - Always called “flexqtl.par”
  - Defines parameters for FlexQTL
    - Some are adjusted automatically through Visual FlexQTL (see further)
    - Some can be adjusted within FlexQTL
    - Need to be “correct” when running FlexQTL independently from Visual FlexQTL
      - E.g. on Linux

FlexQTL™ input files

- FlexQTL Data Prepper output needs additional adjustments
  - Parents without genotype data need to be added
    - Every individual in pedigree needs a data row for FlexQTL
  - Individuals with only one known parent need a second dummy parent
    - FlexQTL only accepts no or 2 parents known

RosBREED
DISEASE RESISTANCE × HORTICULTURAL QUALITY

FlexQTL^{TM} input files

Adjusting FlexQTLDataPrepper file to create 1st FlexQTL input file (data file)

1. Open “.csv” file generated by FlexQTLDataprepper

flexQTL.csv

2. Find individuals with only one parent known/given and add a second ‘dummy’ parent

    -Use “M_[Individual’s name]” for unknown mother

    -Use “F_[Individual’s name]” for unknown father

    -Use “UP_[Individual’s name]” if unclear whether known parent is mother or father

	B	C	D	
ohc	Individual	Parent1	Parent2	Ro
1	Abundance	Napoleon	F_Abundance	A (
1	Summit	Van	F_Summit	A /
1	Sunburst	Summit	F_Sunburst	A /

RosBREED
DISEASE RESISTANCE × HORTICULTURAL QUALITY

FlexQTL™ input files

3. Open ".csv" file generated by FlexQTLDataprepper

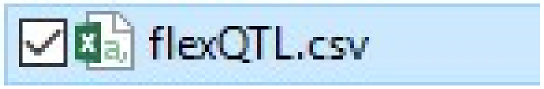

4. Copy both parental columns (columns C and D) to a new sheet

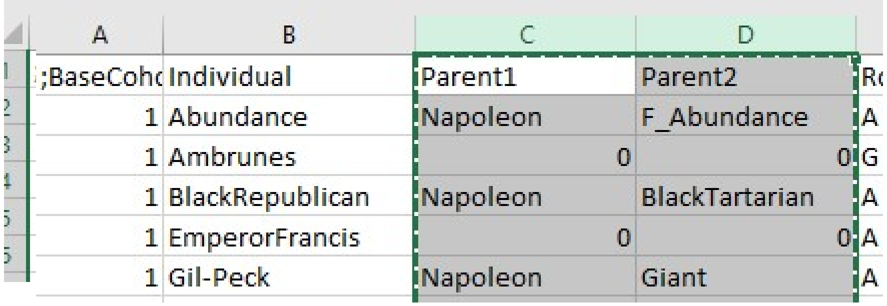

5. Copy "Parent 2" column below "Parent 1" column

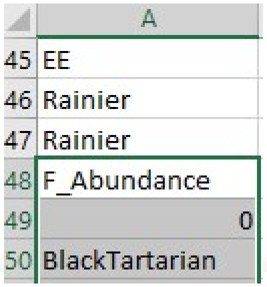

6. Remove duplicates* from combined column
*found under "data" tab in Excel

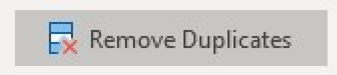

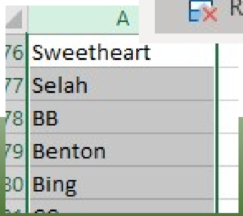

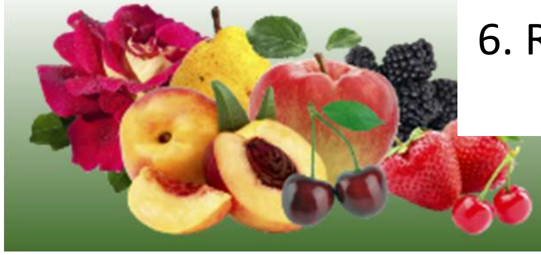

FlexQTL™ input files

7. Count how often each parent is in datafile:

use "COUNTIF" function

Range is "Individual" column (column B) of original sheet

Criteria is parent to be checked (from created column in new sheet)

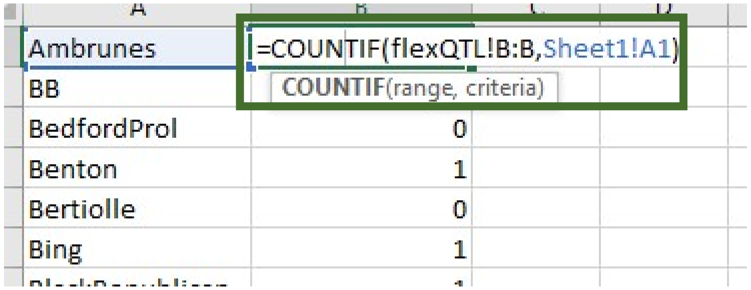

8. Filter for individuals not in the data file (count = 0)

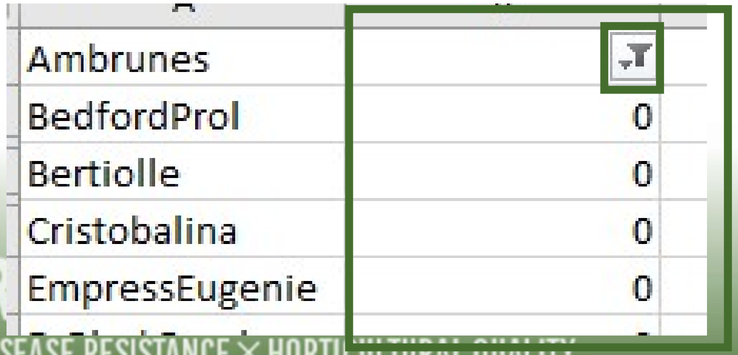

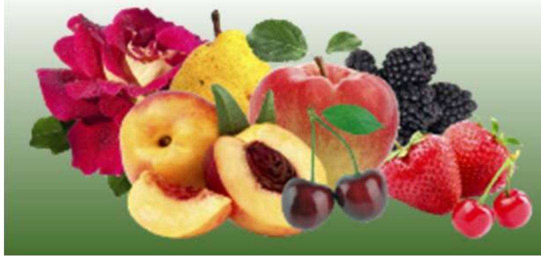

FlexQTL™ input files

9. Copy parents not in data file at the bottom of the data file

A	B
Ambrunes	
BedfordProl	0
Bertiolle	0
Cristobalina	0
EmpressEugenie	0

A	B	C	D	E	
1	Ulster	Napoleon	Gil-Peck	A A	A G
	BedfordProl				
	Bertiolle				
	Cristobalina				
	EmpressEugenie				

10. Fill in "Population" column (Column A), "Pedigree info" (Column C and D), and Genotypic data columns (Column E onwards) of added parents

- "0" for unknown pedigree
- Make sure newly-added pedigree info is also in data set
- "- -" for genotypic data

A	B	C	D	E	F	G	H	I
1	Ulster	Napoleon	Gil-Peck	A A	A G	A G	A G	A G
1	BedfordProl	0	0	- -	- -	- -	- -	- -
1	Bertiolle	0	0	- -	- -	- -	- -	- -
1	Cristobalina	0	0	- -	- -	- -	- -	- -
1	EmpressEugenie	0	0	- -	- -	- -	- -	- -

ROSBREED
DISEASE RESISTANCE × HORTICULTURAL QUALITY

FlexQTL^{TM} input files

11. Add 2 columns between parentage information (Column C+D) and genotypic data (Column E)

12. Fill 1st new column (Column E) with “1”s

*Used as dummy nuisance column

13. Fill 2nd new column (Column F) with random numeric values

*Used as dummy phenotype column

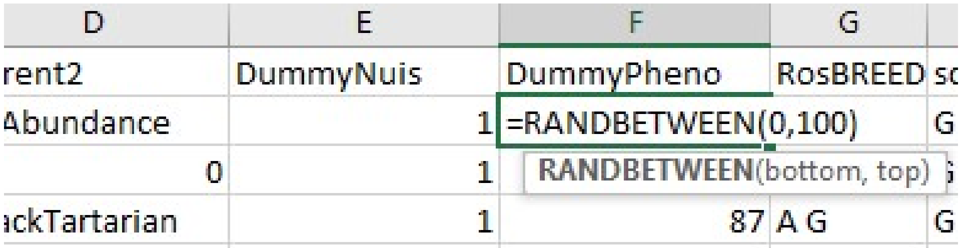

D	E	F	G	
rent2	DummyNuis	DummyPheno	RosBREED	sc
Abundance	1	=RANDBETWEEN(0,100)		G
0	1	RANDBETWEEN(bottom, top)		
ackTartarian	1	87	A G	G

14. Copy full data sheet into text editor (e.g. Notepad++)

15. Save file in text format

16. Change new file’s format extension from “.txt” to “.dat”

FlexQTL™ input files

2nd FlexQTL input file (map file)

17. Use flexQTL.map file generated by FlexQTL Data Prepper

3rd FlexQTL input file (parameter file)

18. Save Suppl. Table 4c as text file

    * “datafile”, “mapfile”, “indiC” and “nmrkrC” parameter settings will be updated automatically when loading files into Visual FlexQTL

19. Change file extension from “.txt” to “.par”

20. Change file name to “flexqtl”

    FlexQTL does not accept any other name!

United States Department of Agriculture

National Institute of Food and Agriculture

Installing (Visual) FlexQTL™

RosBREED

DISEASE RESISTANCE × HORTICULTURAL QUALITY → SUPERIOR CULTIVARS

Installation

- Install Visual FlexQTL
  - Will also install FlexQTL
- Install following packages in R
  - Data.tables
  - Plyr
  - Lattice

Installation

- Tools > Settings
  - General Tab
    - Where is Rscript.exe on pc
    - Where is flexqtl_console.exe on pc
    - Where is postqtl_console.exe on pc
    - Where can license be founds
      - Needs updating every year

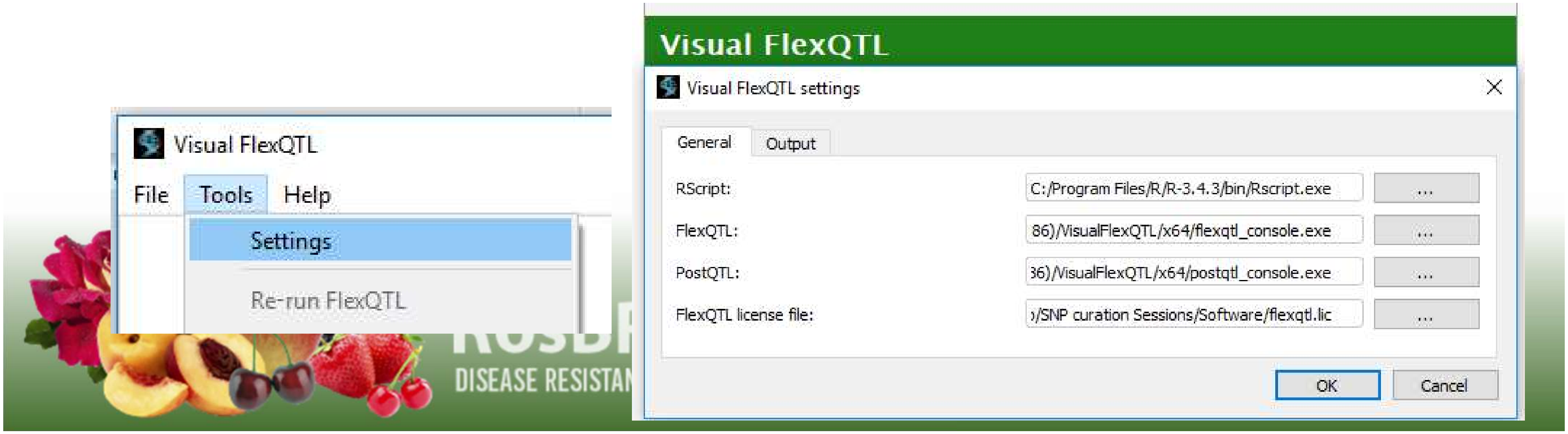

Installation – Win8 or later

- Install Visual C++ Redistributable for Visual Studio 2012
  - https://www.microsoft.com/en-us/download/details.aspx?id=30679

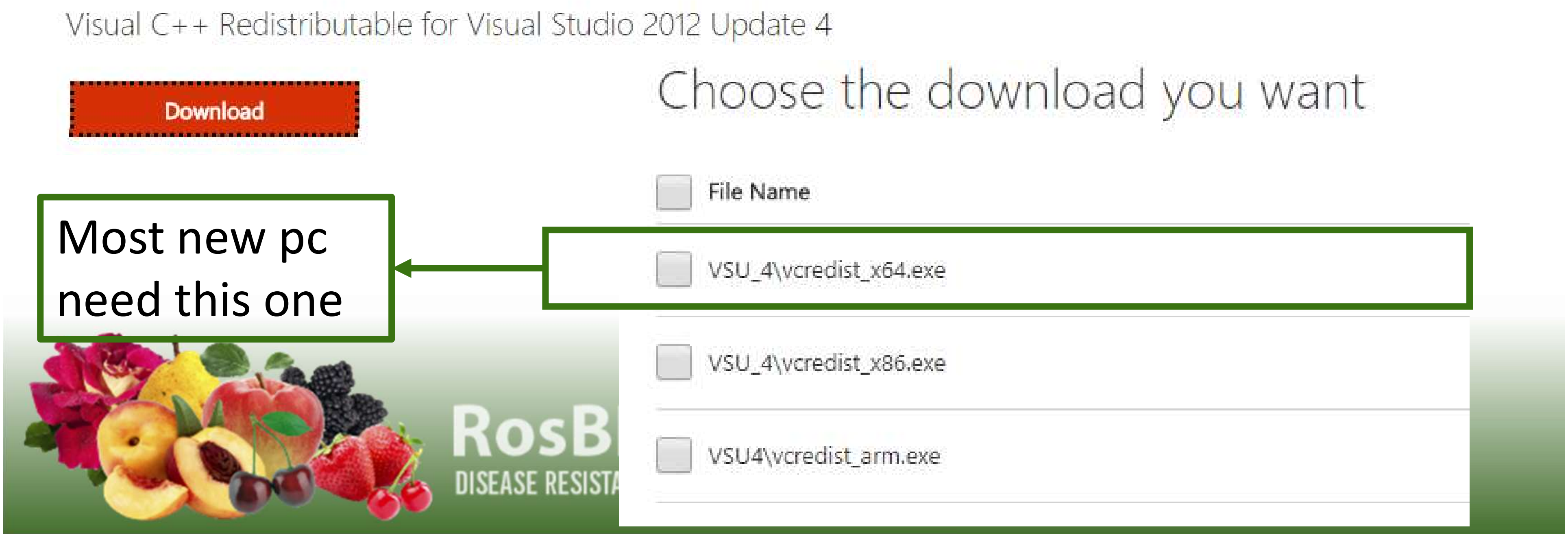

United States Department of Agriculture

National Institute of Food and Agriculture

Finding Mendelian-inconsistent errors

RosBREED

DISEASE RESISTANCE × HORTICULTURAL QUALITY → SUPERIOR CULTIVARS

Creating project in Visual FlexQTL™

1. Open Visual FlexQTL™

2. Under “File”, choose “New Project”

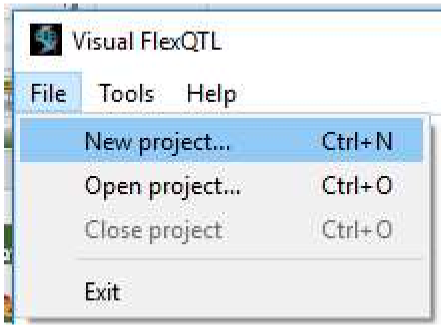

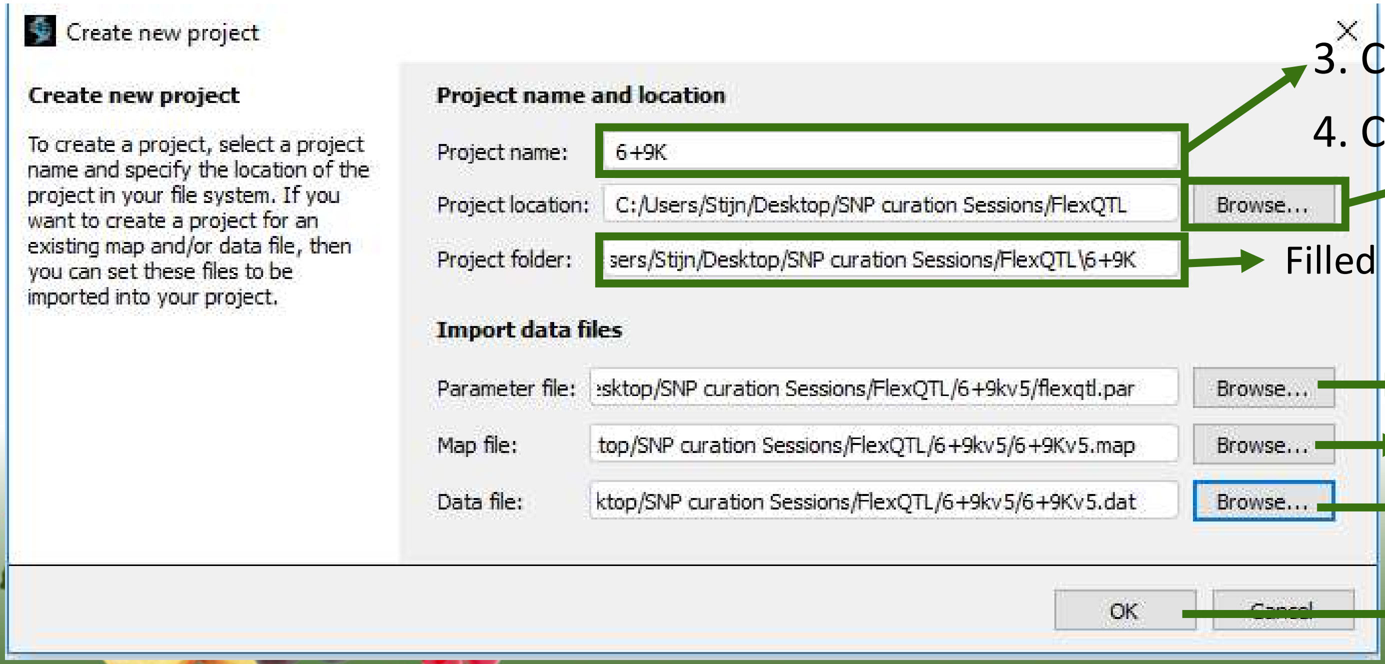

3. Choose name for project

4. Choose project directory

Filled in automatically

4. Load parameter file

5. Load map file

6. Load data file

6. Press “OK”

Creating project in Visual FlexQTL™

1. Open Visual FlexQTL™

2. Under “File”, choose “New Project”

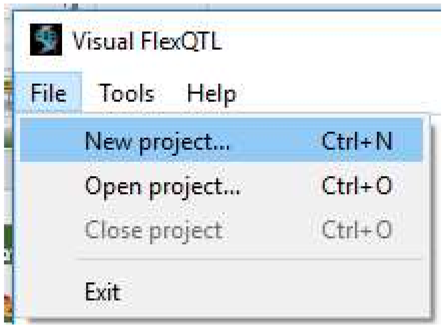

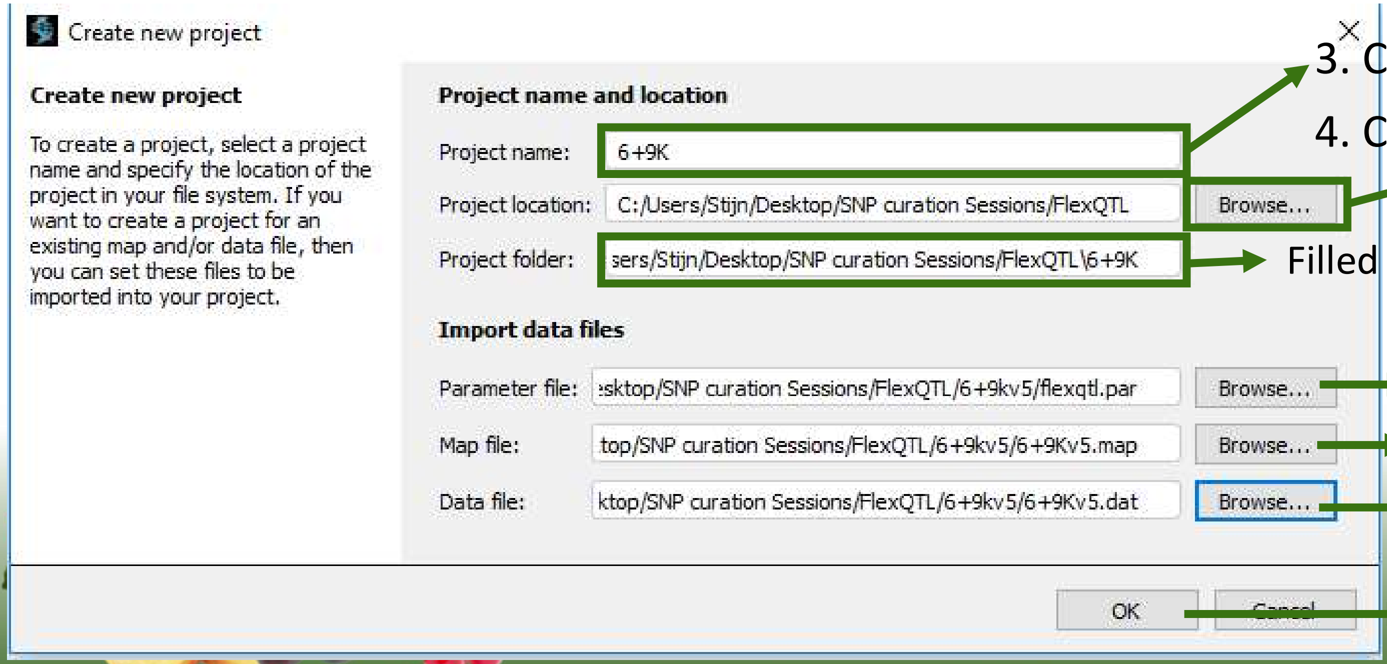

3. Choose name for project

4. Choose project directory

Filled in automatically

4. Load parameter file

5. Load map file

6. Load data file

6. Press “OK”

Checking parameter settings

7. Check parameter settings under the ‘Settings’ tab

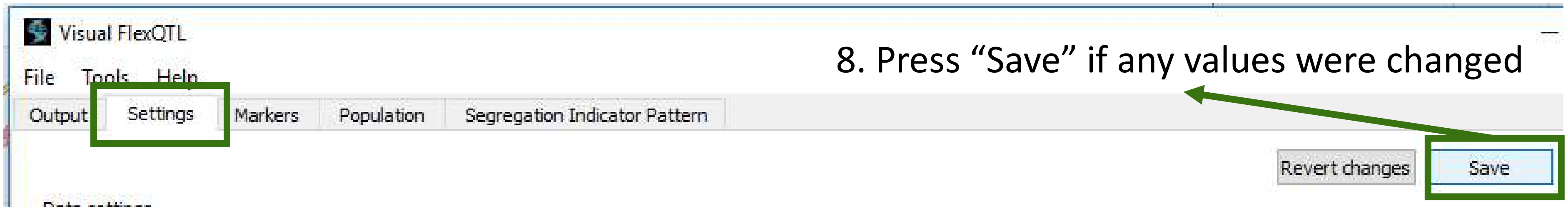

8. Press “Save” if any values were changed

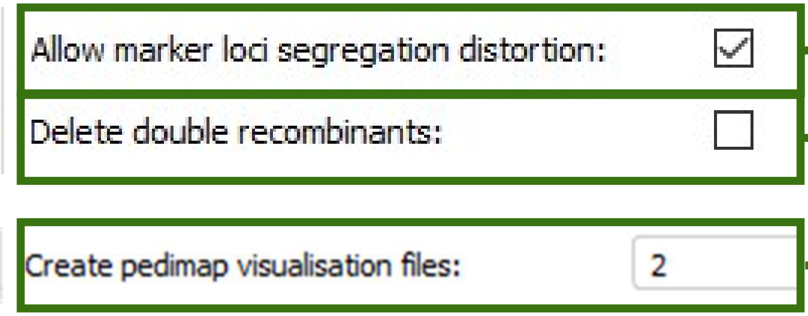

Checked to allow for segregation distortion

Unchecked to keep genotypic data for single marker double recombinations

“2” for an early stop; generates files for error checking but does not do full FlexQTL^{TM} analysis (can take days)

Running FlexQTL™

8. Run FlexQTL (Tools>Re-run FlexQTL)

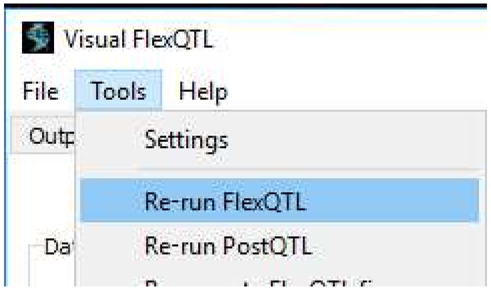

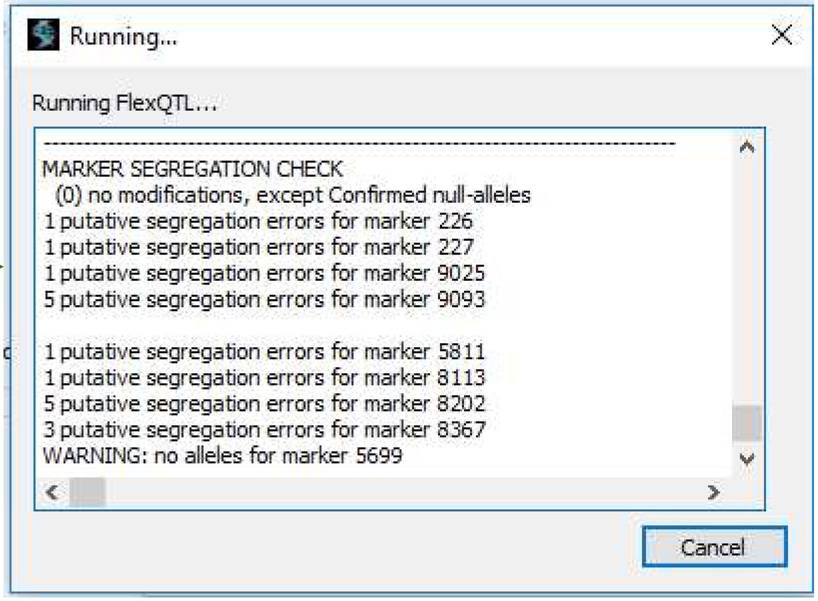

9. Once run, go to project directory and open "mconsistency.csv"

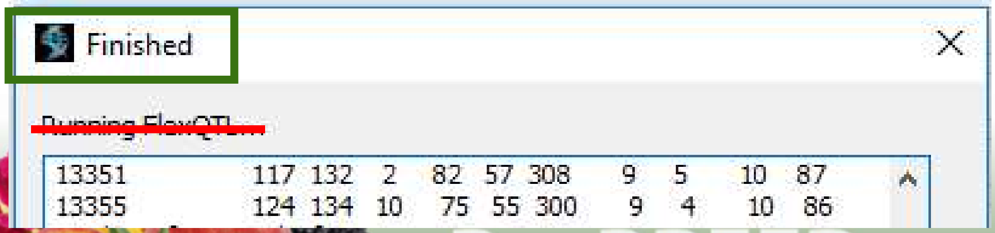

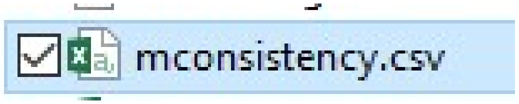

10. Save file in ".xls(x)" format

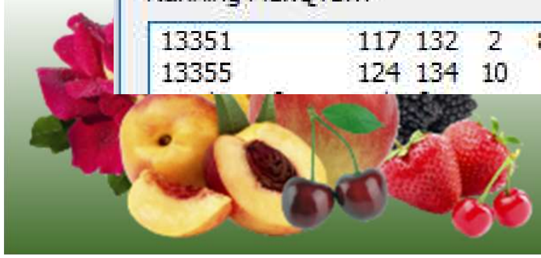

Mconsistency.csv

Information found in mconsistency file (1)

Code_Ped	Symbol	ShortNam	Description
0	.	OC	observed & consistent
1	&	MA	missing & augmented
2	}	OEA	observed & deleted error & augmented
3	]	pnuA	putative null-allele & augmented
4	~	pnuM	putative null-allele & missing
5	-	IM	incomplete & missing
6	/	OpE	observed & putative error
7	{	OE	observed & deleted error
8	[	pnuE	putative null-allele & error
9	#	ME	missing & error

Consistensies and errors reported by the mconsistency file

marker								
position								
	SUM({[#)	{	[	#	}	/	SUM(}/)	~
SUM({[#)	1180							
{		351						
[			0					
#				829				
}					484			
/						15		
SUM(}/)							499	
~								0

Summary of errors found in data set

Mconsistency.csv

Information found in mconsistency file (2)

Summary of errors found for each individual

	SUM({[#)	{	[	#	}	/	SUM(}/)	~
Napoleon	0	0	0	0	0	0	0	0
F_Abunda	0	0	0	0	0	0	0	0
Abundanc	0	0	0	0	0	0	0	0
Ambrunes	2	2	0	0	6	0	6	0

Summary of errors found for each marker

marker	4071	8853	8854
position	100	100.0362	100.0364
SUM({[#)	1	1	0
{	0	0	0
[	0	0	0
#	1	1	0
}	0	0	0
/	0	0	0
SUM(}/)	0	0	0
~	0	0	0

Observation (consistent genotype or erroneous genotype) for each individual and marker combination

marker	4071	8853	8854	8855
position	100	100.0362	100.0364	100.0365
Napoleon	-	&	&	&
F_Abunda	-	-	-	-
Abundanc	.	.	-	-
Ambrunes	.	.	.	-
Angela	.	.	.	.
Blackhear	-	-	-	-
Lambert	.	.	.	.
JI2420	-	-	-	-
Stella	.	.	.	.

Mconsistency.csv

11. Copy data from row 15 onward and all columns

13	;	9	#	ME	missing & error							
14												
15	marker									4071	8853	
16	position									100	100.0362	[illegible]
17		SUM({[#)	{	[	#	}	/	SUM(}/)	~			
18	SUM({[#)	1180								1	1	
19	{		351							0	0	
20	[			0						0	0	
21	#				829					1	1	
22	}					484				0	0	
23	/						15			0	0	
24	SUM(}/)							499		0	0	
25	~								0	0	0	
26	11_118	0	0	0	0	0	0	0	0	.	.	.
27	13_20	0	0	0	0	0	0	0	0	.	.	.
28	5_62	0	0	0	0	0	0	0	0	.	.	.
29	6_240	0	0	0	0	0	0	0	0	.	.	.
30	99F_131RJ	0	0	0	0	0	0	0	0	.	.	.

12. Transpose copied data in new sheet

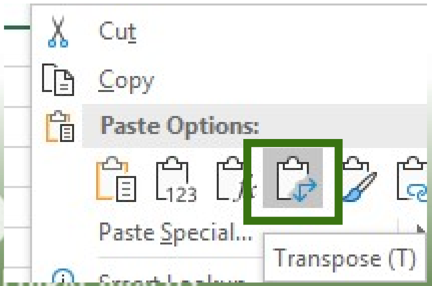

Mconsistency.csv

13. Sum all errors for each marker in column C

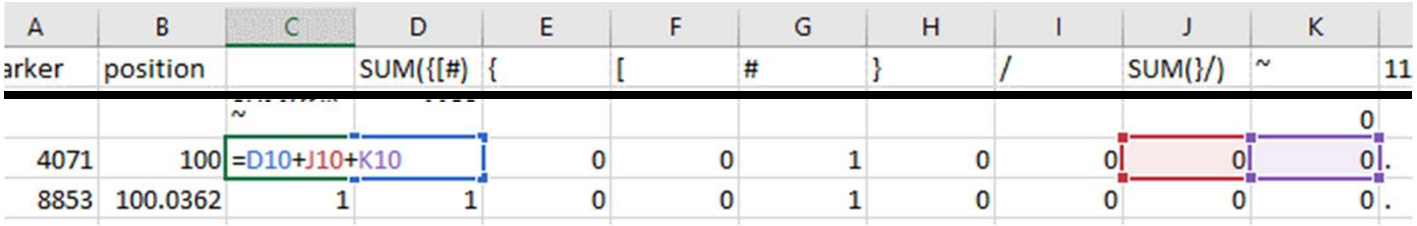

14. Add filter and filter for markers with at least 1 error

*optional: sort full data according to number of errors from high to low

15. Copy all data and transpose into new sheet

*original mconsistency format but only markers with errors are kept

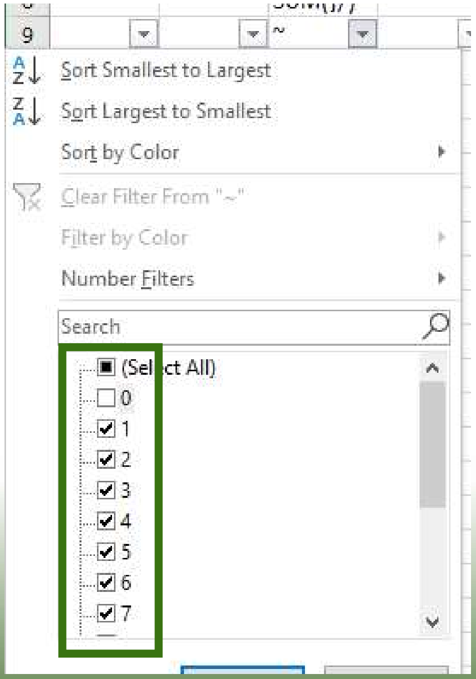

RosBREED
DISEASE RESISTANCE × HORTICULTURAL QUALITY

Mconsistency.csv

16. Freeze panes so that summary for each marker and individual is locked

17. Add filter to last summary row

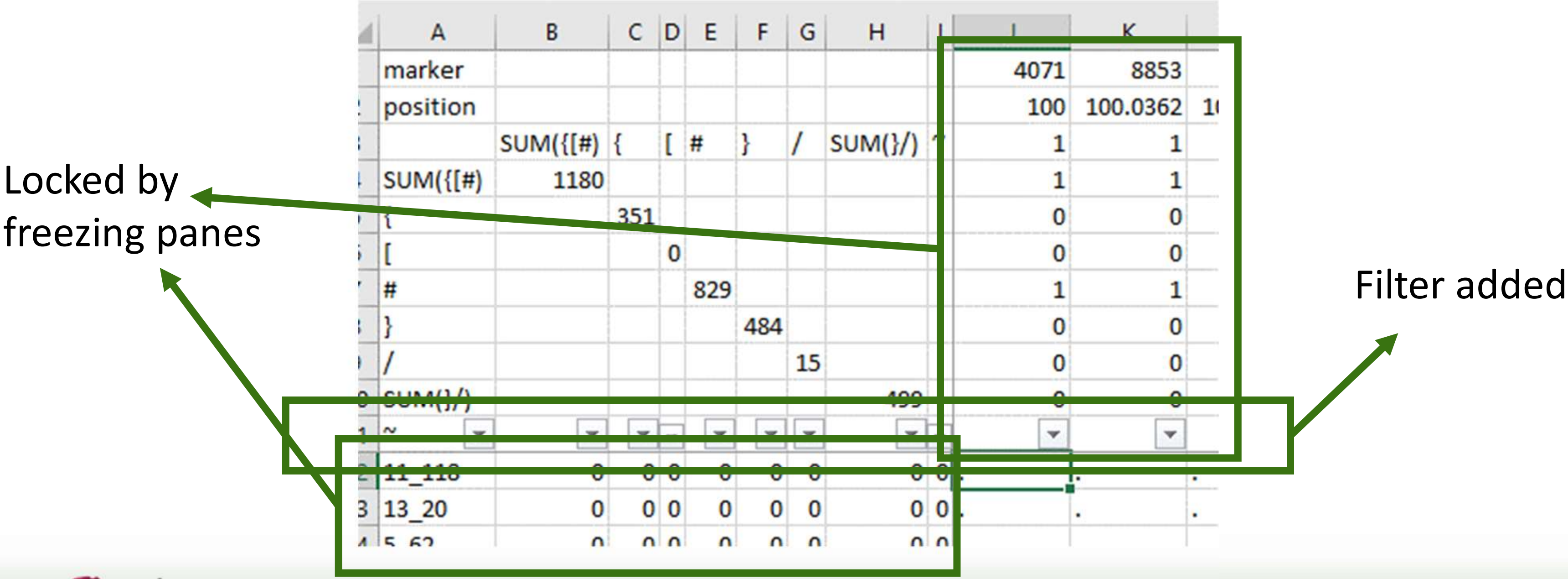

Mconsistency.csv

18. For each marker, filter for characters that are associated with an error to find which individuals are causing an error in column A

	A	B	C	D	E	F	G	H	I	AEJ
1	marker									2977
2	position									600.4256
3		SUM({[#)	{	[	#	}	/	SUM(}/)	~	11
4	SUM({[#)	1180								3
5	{		351							3
6	[			0						0
7	#				829					0
8	}					484				7
9	/						15			1
0	SUM(}/)							499		8
1	~									
5	BlackRepu	1	1	0	0	0	0	0	0	{
4	Lapins	3	3	0	0	7	0	7	0	}
3	Rainier	17	17	0	0	15	0	15	0	}
6	Regina	0	0	0	0	12	0	12	0	}
23	Selah	4	4	0	0	8	0	8	0	{
32	PMR-1	0	0	0	0	6	1	7	0	/
54	Cowiche	32	32	0	0	56	0	56	0	{
[illegible]	[illegible]	0	0	0	0	1	0	1	0	}

Error-associated characters

ec	Symbol	ShortNam	Description
2	}	OEA	observed & deleted error & augmented
6	/	OpE	observed & putative error
7	{	OE	observed & deleted error
8	[	pnuE	putative null-allele & error
9	#	ME	missing & error

Filtered for error-associated characters

Individuals who caused an error for this marker

*Could also be their parents (or ancestors if parents have missing data)

*Could also be their offspring (very often the case)

Resolving errors

19. Check genotypic data to find what is causing the issue

    *Note: sometimes imputed missing data is causing the issue

    e.g. parental genotypic data is missing but grandparents are both “AA” -> parent is imputed as “AA” however individual is “BB” -> error reported

    *Note: In case of multiple parents/ancestors with missing data that prevented earlier pedigree checking, pedigree could still be incorrect

20. Check GenomeStudio® to:

    -Ensure genotypic data matches

    -Genotype clustering is correct (e.g. additional clusters or null alleles observed)

    -Single individuals are assigned the correct genotype

Incorrect clustering

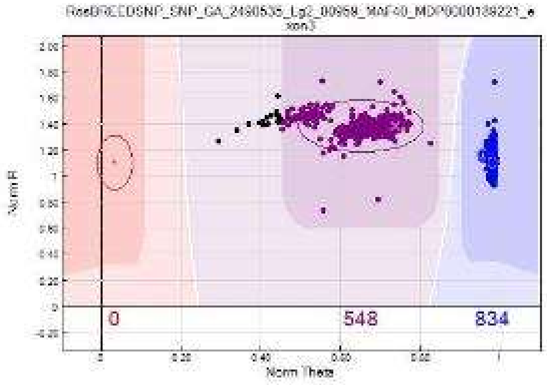

Incorrect clustering due to additional clusters

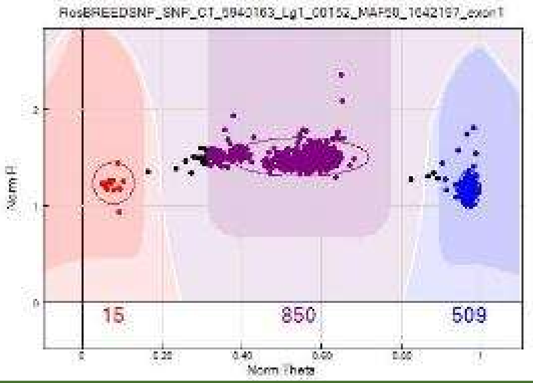

Incorrect clustering due to null alleles

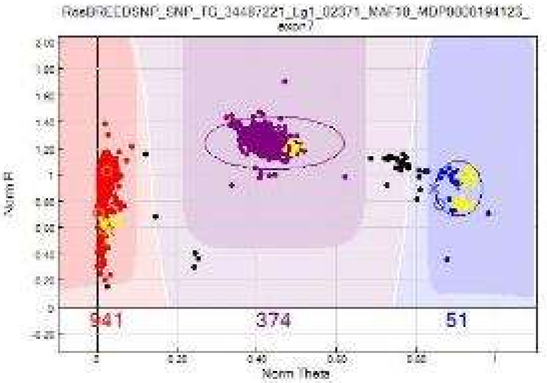

Resolving errors

21. Update data file (pedigree or genotypic data)

    *Update map file if markers were removed from data set

22. Re-run FlexQTL^{TM} and check mconsistency file to ensure errors are resolved

22. Repeat until no errors are reported

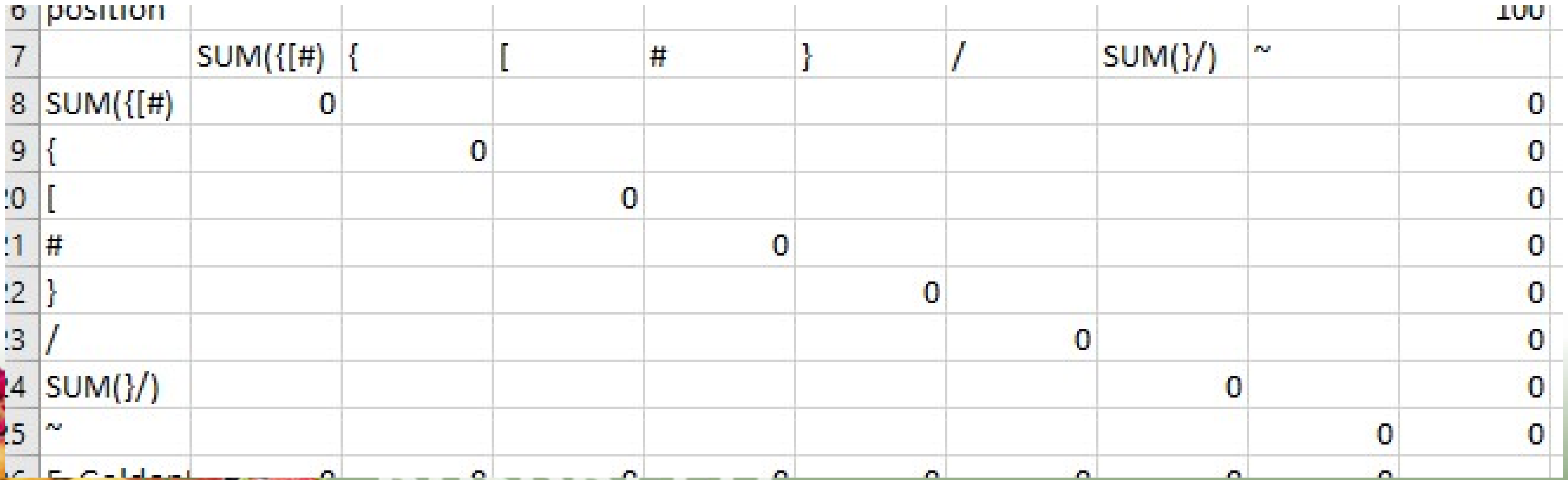

position									100
	SUM({[#)	{	[	#	}	/	SUM(}/)	~	
SUM({[#)	0								0
{		0							0
[			0						0
#				0					0
}					0				0
/						0			0
SUM(}/)							0		0
~								0	0

USDA

United States
Department of
Agriculture

National Institute
of Food and
Agriculture

Finding Mendelian-consistent errors

RosBREED

DISEASE RESISTANCE × HORTICULTURAL QUALITY → SUPERIOR CULTIVARS

Finding double recombinations

1. Run FlexQTL^{TM} with latest map and data file that did not lead to any reported errors
    *Parameter settings remain the same

2. Set interval for which double recombinations (DR) (Tools>Calculate>(Re-)Compute recombination sequences)
    *Default is all DR within 10 cM

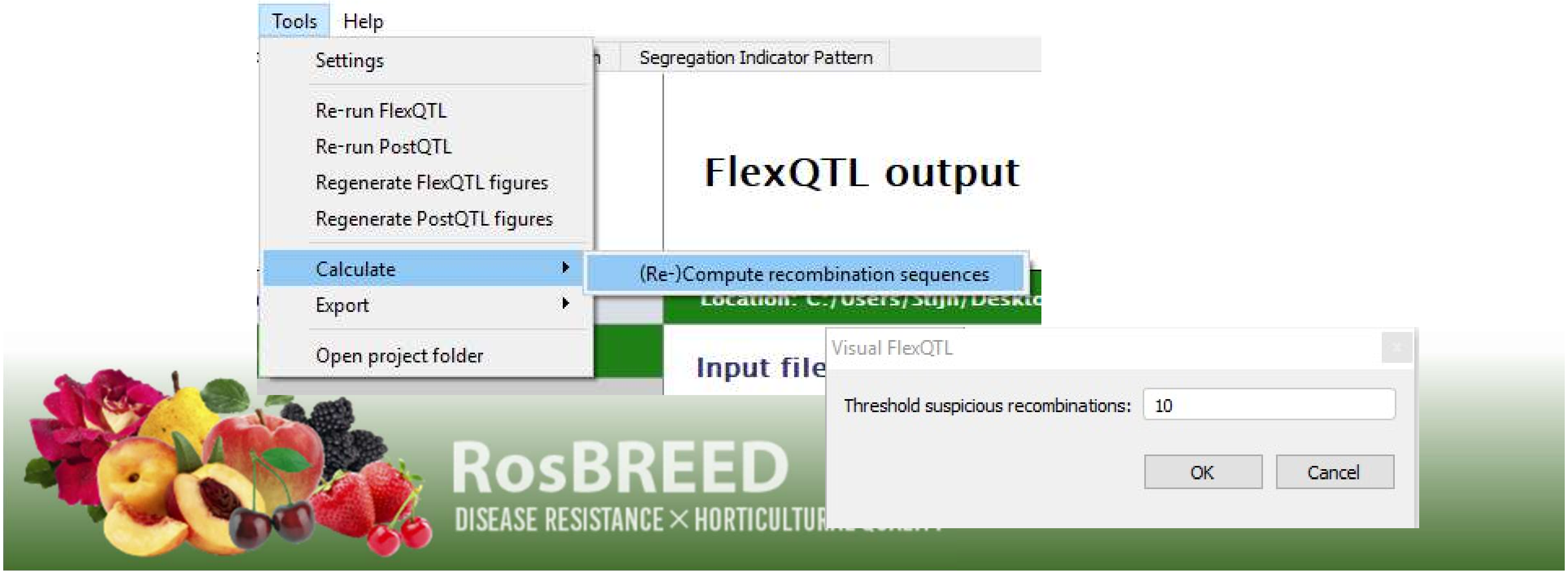

Finding DR

3. Export all double recombinations within defined interval (Tools>Export>Export recombination sequences file; then select directory to save file in)

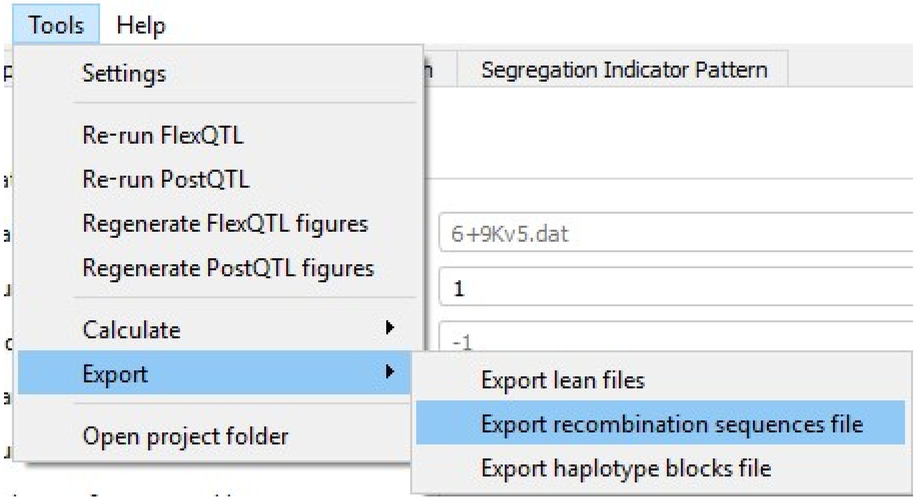

4. Open generated file called “DoubleRecombinations.csv”

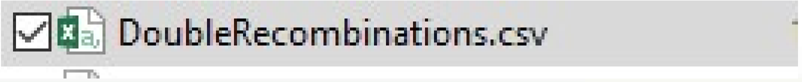

5. Save as “.xls(x)” format

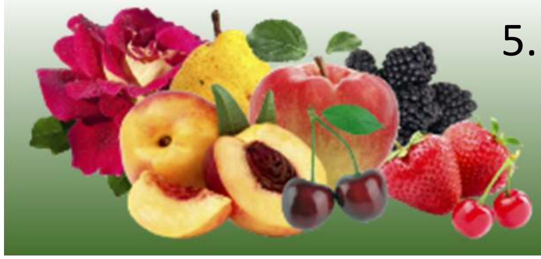

Finding DR

Information found in DoubleRecombinations file

Individual in which DR is observed

Chromosome on which DR is observed

How suspicious/unlikely is this DR

Length of DR interval

	A	B	C	D	E	F	G	H	I	J	K	L	M
1	Individual	Chromosc	Homolog	M1	M2	M3	M4	P1	P2	P3	P4	R	Suspicion
2	Abundanc	1	0	8112	8202	8202	453	47.82	53.41	53.41	56.17	8.35	0.073106
3	Abundanc	1	0	8202	453	453	8255	53.41	56.17	56.17	57.31	3.9	1
4	Abundanc	1	0	509	8343	8359	8367	76.07	78.47	80.13	80.55	4.48	0.999835

Homolog on which DR is observed

0: Homolog coming from mother

1: Homolog coming from father

-M/P1: Last marker name(M)/position(P) before 1st recombination

-M/P2: First marker name(M)/position(P) after 1st recombination

-M/P3:Last marker name(M)/position(P) before 2nd recombination

-M/P4: First marker name(M)/position(P) after 2nd recombination

Finding DR

5. Sort file according to chromosome and position of DR

6. Look first for regions with many DR

7. Use 'Segregation Indicator Pattern' tab of Visual FlexQTL™ to visualize DR
   Use drop-down menu on top-right to choose "Recombination sequences (based on FlexQTL SIP)

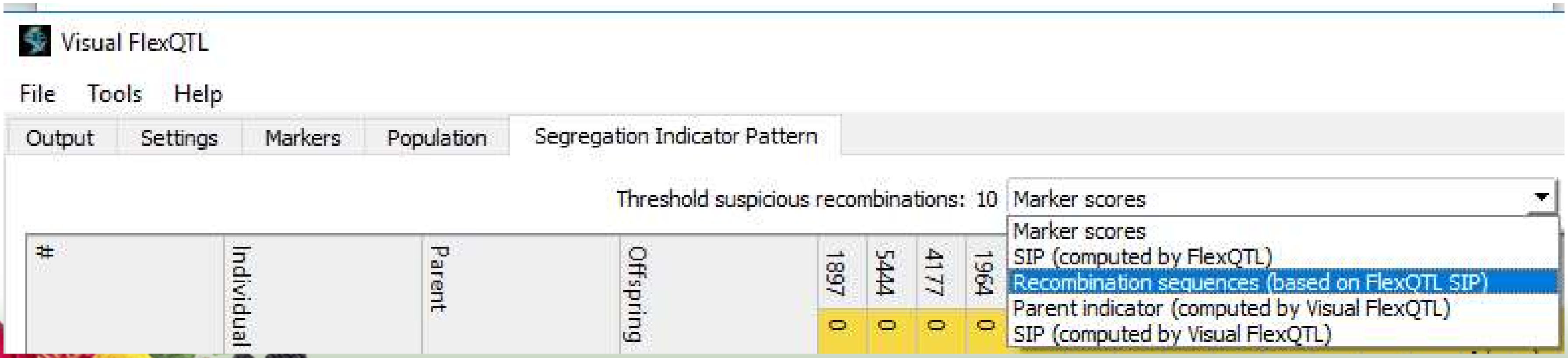

Investigating DR

Information found under "Segregation Indicator Pattern" tab

Indicator of grandparental origin:
-0: Allele originates from mother of parent (grandmother; yellow)
-1: Allele originates from father of parent (grandfather; blue)

Recombination region (0-to-1 or 1-to-0 switch; orange)

Parent's name

DR region (0-to1-to-0 or 1-to-0-to-1 switches; red)

Individual's name

2 lines for each individual, one for each parent's homolog

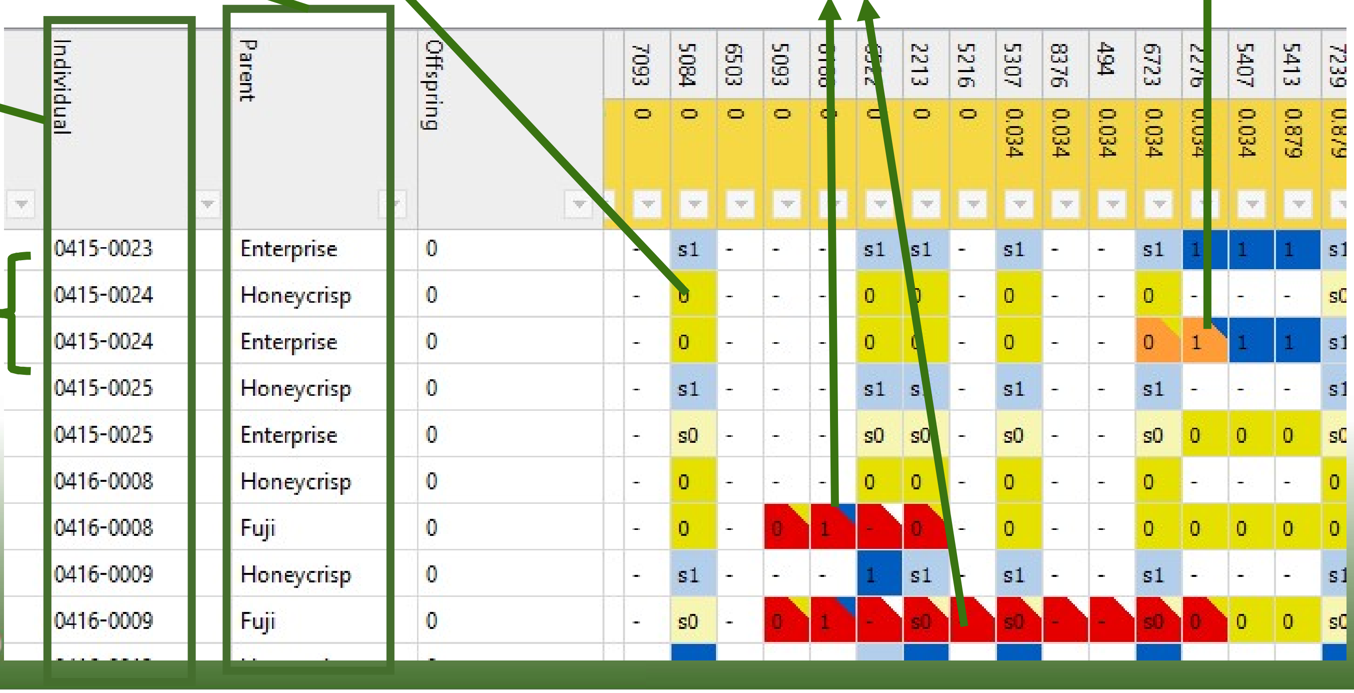

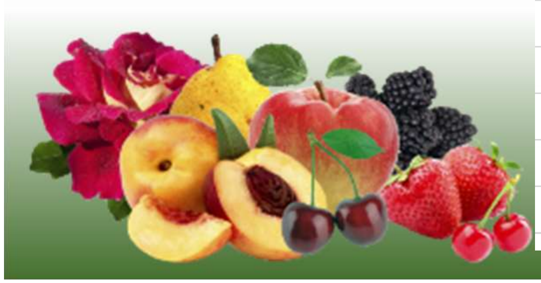

Resolving DR

8. Check genotype calls with GenomeStudio®

-e.g. incorrect clustering of “AA” as “AB” ->

“AB” (actually “AA”) x “AB” -> 100% “AB” (actually 50% “AA” and 50% “AB”)

*does not lead to PPC errors

*often characterized by many DR in a single or few families

*often characterized by a single marker involved in DR

-single genotyping error in individual

*does not lead to PPC errors

*often characterized by many DR in offspring of this individual

*often characterized by a single marker involved in DR

Incorrect clustering

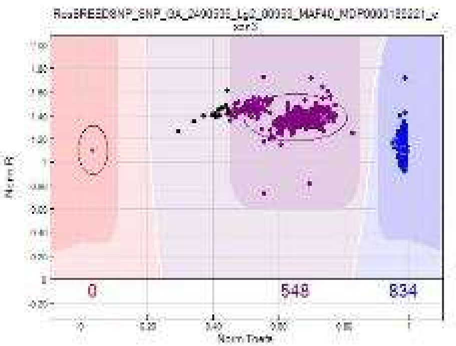

Resolving DR

9.a Check for errors in map order using the “Segregation Indicator Pattern” tab

Moving marker(s) a few positions resolves DR

*Characterized by DR in a few individuals who are not necessarily related

*Characterized by DR spanning one or multiple markers

*Often characterized by a third recombination nearby

moving markers to this recombination would solve DR

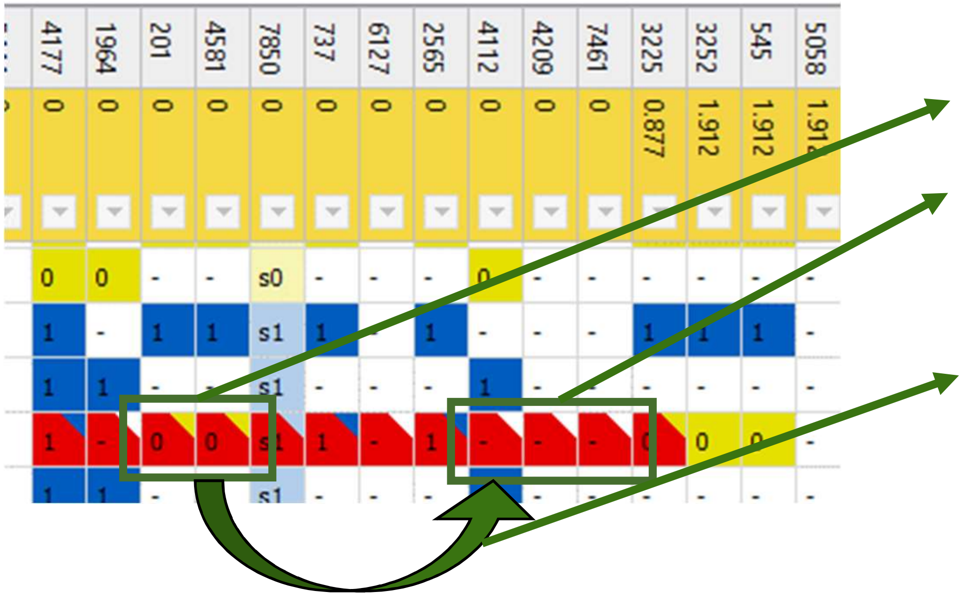

DR

3rd recombination

Moving markers “201” and “4581” behind marker “2565” would resolve these DR

*This cannot create new DR for other individuals (next slide)

Resolving DR

9.b Adjust marker position and check if no new DR are created

9.b.1 Open “SIP_Population_X.csv” in FlexQTL project folder

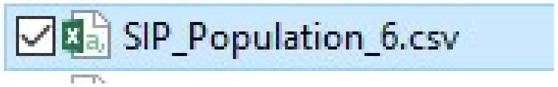

*“X” is highest number found in folder

*Interpretation the same as “Segregation Indicator Pattern” tab of Visual FlexQTL™

9.b.2 Use conditional formatting to color “0”/“s0” yellow and “1”/“s1” blue

*same coloring as “Segregation Indicator Pattern” tab of Visual FlexQTL™

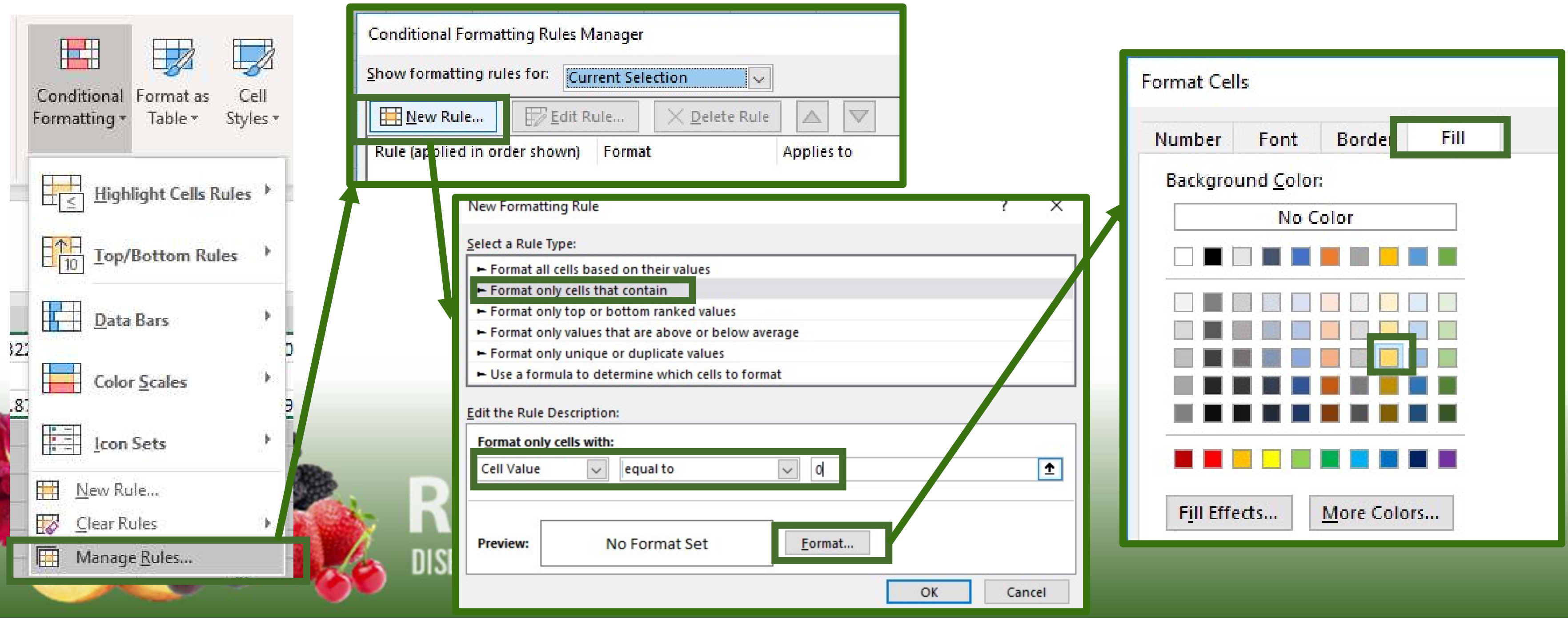

Resolving DR

9.b Adjust marker position and check if no new DR are created

9.b.1 Open “SIP_Population_X.csv” in FlexQTL project folder

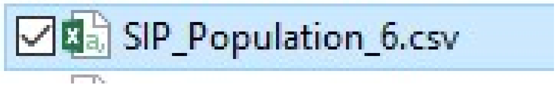

*“X” is highest number found in folder

*Interpretation the same as “Segregation Indicator Pattern” tab of Visual FlexQTL™

9.b.2 Use conditional formatting to color “0”/“s0” yellow and “1”/“s1” blue

*same coloring as “Segregation Indicator Pattern” tab of Visual FlexQTL™

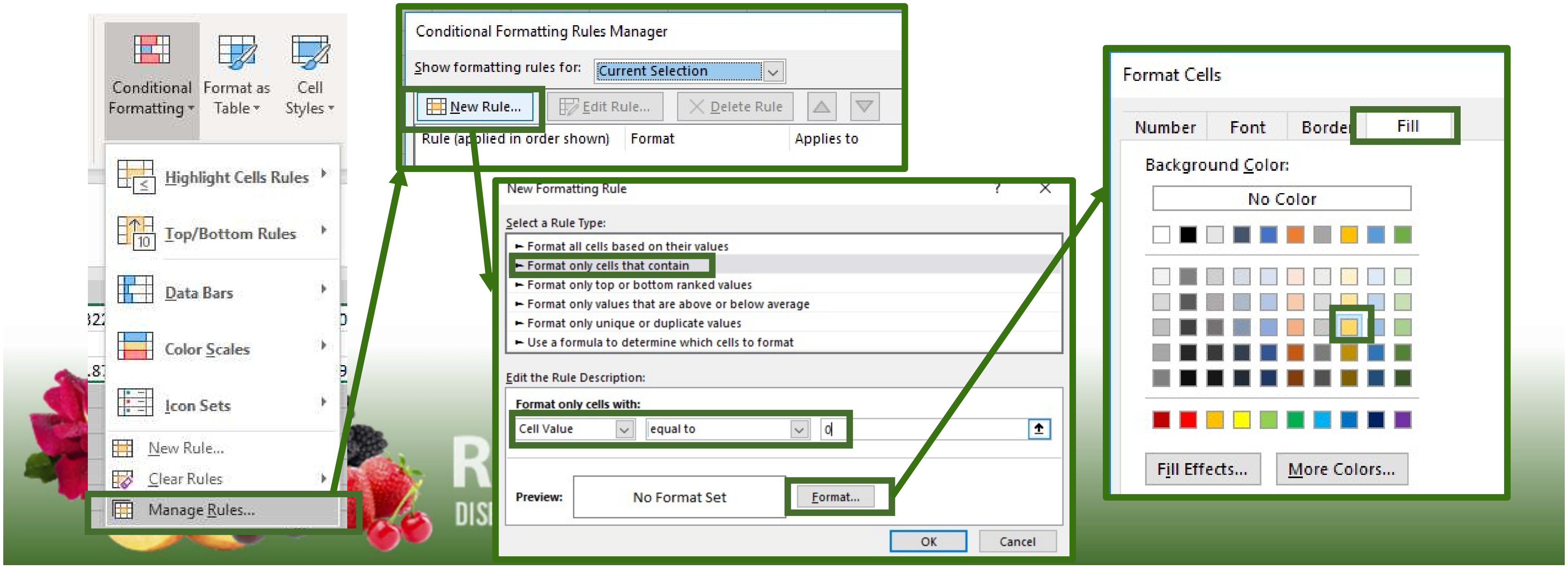

Resolving DR

9.b.3 Cut columns of markers to move and insert them into new position

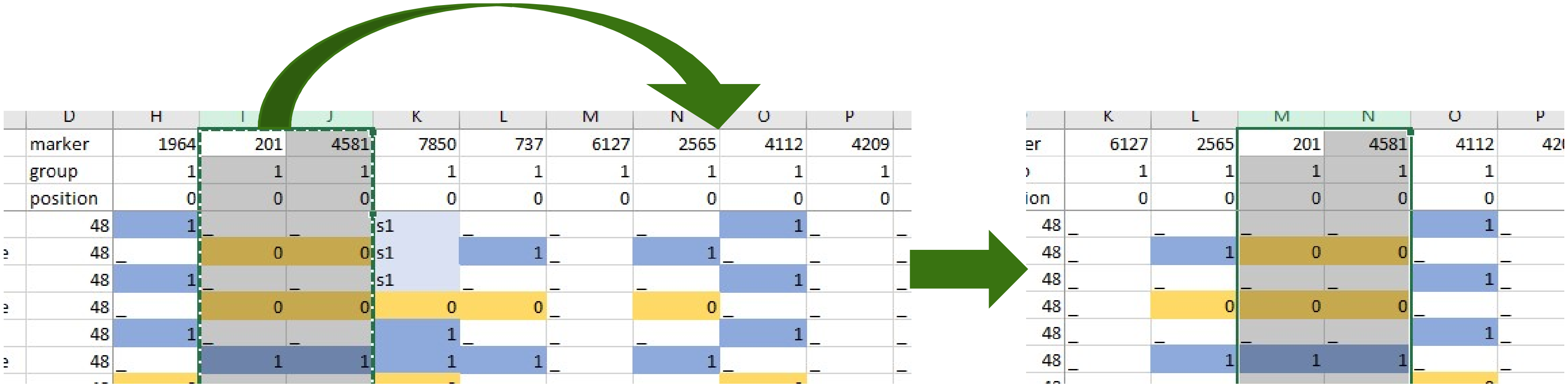

9.b.4 Check rest of the file (rows) to make sure this move did not create additional DR

9.b.5 Update ".dat" and ".map" file to reflect the change in marker positions

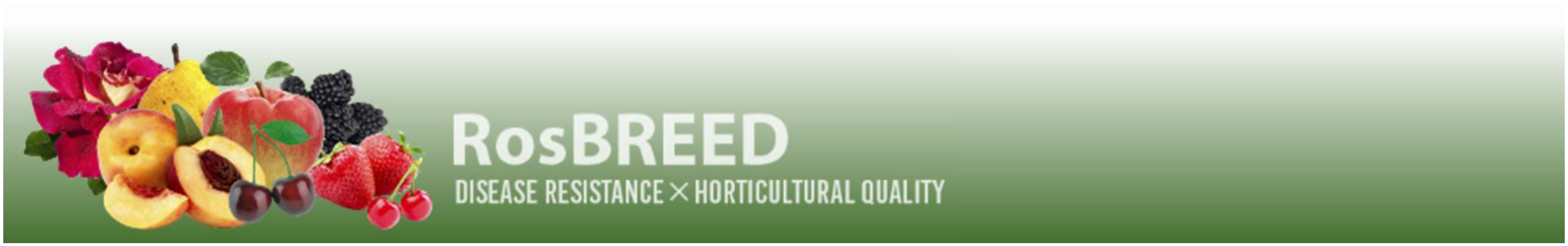

Resolving DR

10. Check for phasing issues

*Often characterized by DR in almost all offspring of a single individual

*Individuals is often a founder (parents not genotyped)

*One or more offspring have a single recombination for same marker(s)

*Incorrect phasing of this individual causes errors for sibs

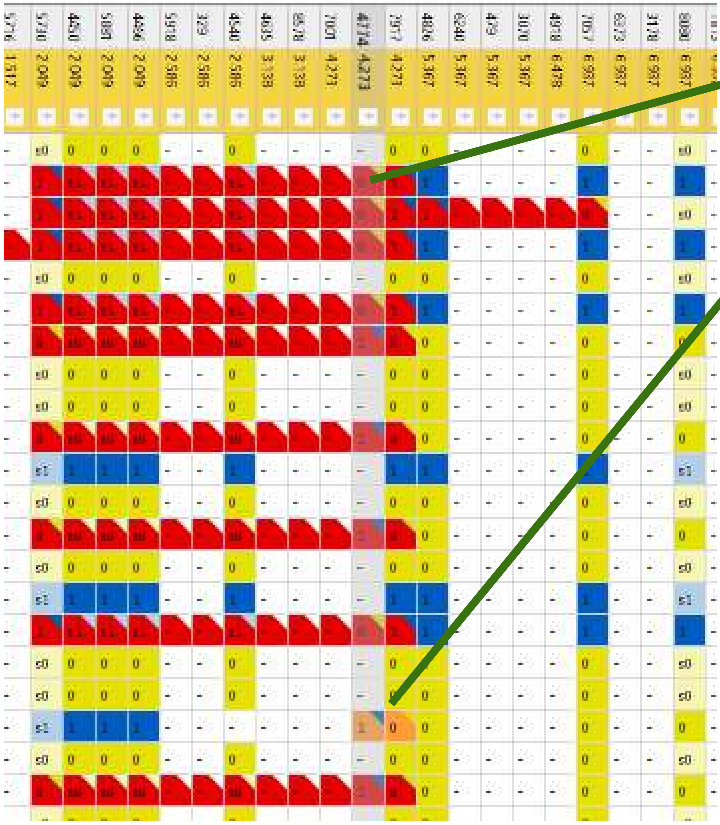

Marker always involved in DR

One individual with single recombination

*Make genotypic data of this individual missing (gets imputed again in later stages)

<u>Probable cause: minimizing recombination interval</u>

Incorrect phasing – short recombination interval

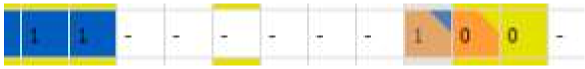

Correct phasing – long recombination interval

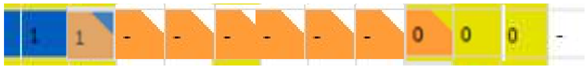

Resolving DR

11. Re-run FlexQTL™ and re-generate “DoubleRecombinations.csv” file with Visual FlexQTL™ after resolving major DR

    *Resolving DR may resolve other nearby DR

12. Repeat these steps until remaining DR are very likely true

    *Unlike Mendelian-inconsistent errors, some DR can remain

USDA

United States Department of Agriculture

National Institute of Food and Agriculture

Haploblocking and haplotyping

RosBREED

DISEASE RESISTANCE × HORTICULTURAL QUALITY → SUPERIOR CULTIVARS

USDA

United States
Department of
Agriculture

National Institute
of Food and
Agriculture

Generating haploblocks

RosBREED

DISEASE RESISTANCE × HORTICULTURAL QUALITY → SUPERIOR CULTIVARS

Adjusting input files

- Accurate phasing requires:
  - Removal of intermediate ungenotyped progenitors
    - Well-represented ancestors (>3 offspring) whose genotype can be imputed can remain
- Haploblock determination based on historical recombination requires:
  - All individuals that need to be taken into account to have offspring in data set

RosBREED
DISEASE RESISTANCE × HORTICULTURAL QUALITY

Adjusting input files

1. Remove any parentage information of ungenotyped intermediate progenitors from FlexQTL™ data input file
    - *Ungenotyped founders (no known parents) can stay in data set
    - *Ungenotyped progenitors with more than 3 genotyped offspring can stay in data set

2. Add dummy offspring to FlexQTL™ data input file for any individual whose recombination are important for haploblock determination (e.g. breeding selections) that don't have any offspring in the data set
    - *Set genotypic data of dummy individuals to missing
    - *Set parents of dummy individuals to individuals who need offspring in data set

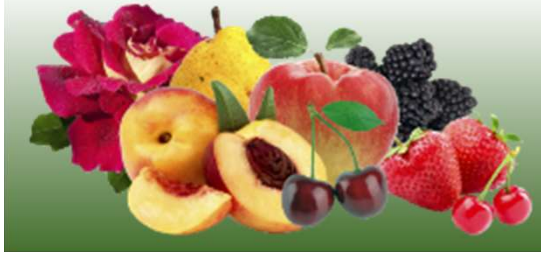

Creating haploblocks

3. Create a new FlexQTL™ project and use following settings
- skipSampleMarkers: 0
- REDprint: 0
- Markerblock: 5
- MSegDelta: 1
- DeleteDR: 1

4. Run FlexQTL™

5. Generate haploblock delimitation with Visual FlexQTL™ (Tools>Export>Export haplotype blocks file then select directory to save file in)

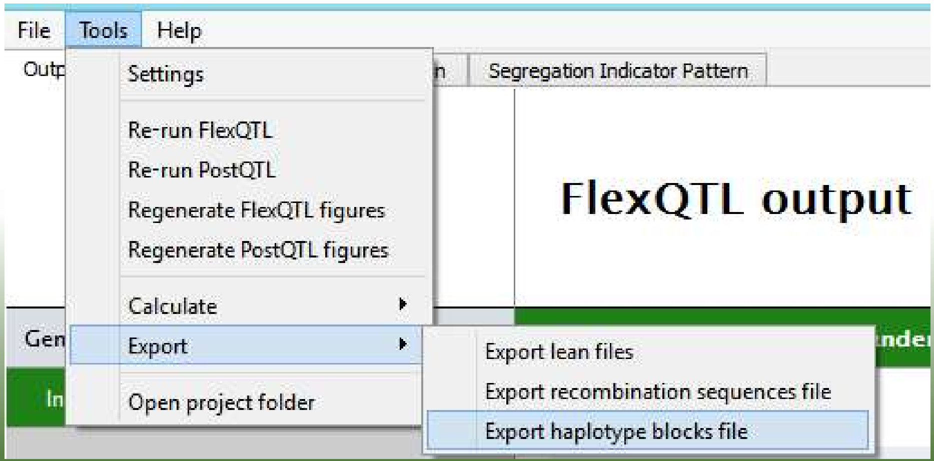

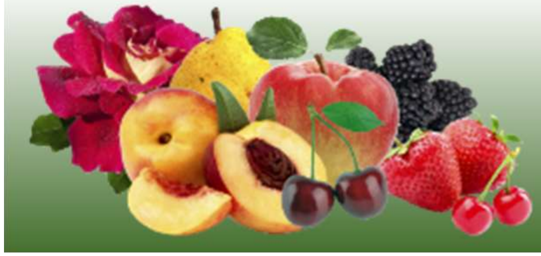

Creating haploblocks

6. Open generated HaploBlock.map file

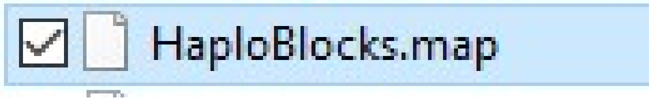

7. Adjust Haploblock designations as wanted

e.g. to ensure maximum size of haploblock is 1 cM

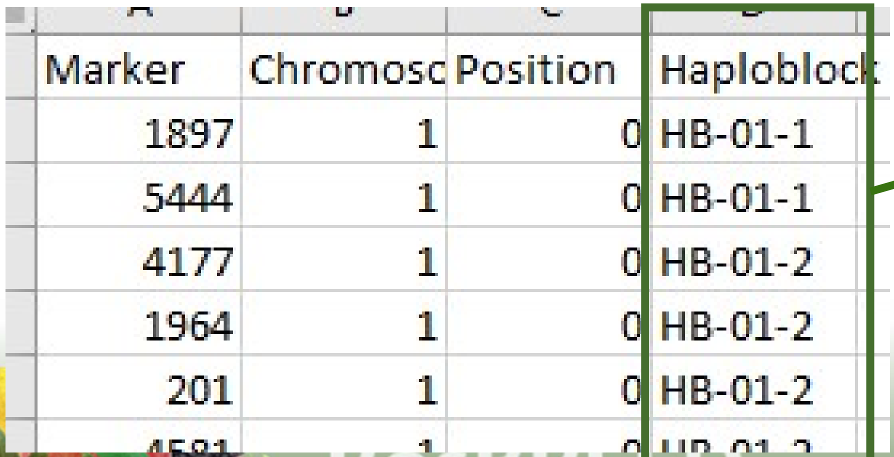

Marker	Chromosc	Position	Haploblock
1897	1	0	HB-01-1
5444	1	0	HB-01-1
4177	1	0	HB-01-2
1964	1	0	HB-01-2
201	1	0	HB-01-2
[illegible]	[illegible]	[illegible]	[illegible]

Assigned haploblock
*name can be
changed freely

United States Department of Agriculture

National Institute of Food and Agriculture

Determining haplotypes

RosBREED

DISEASE RESISTANCE × HORTICULTURAL QUALITY → SUPERIOR CULTIVARS

Creating input files

1. Remove dummy offspring from FlexQTL™ data input file used for haploblock determination

2. Create a new FlexQTL™ project and use following settings
    - skipSampleMarkers: 0
    - REDprint: 0
    - Markerblock: 5
    - MSegDelta: 1
    - DeleteDR: 1

3. Run FlexQTL™

RosBREED
DISEASE RESISTANCE × HORTICULTURAL QUALITY

Creating input files

4. Copy needed input files in a single folder
   *From latest FlexQTL™ run:
   - mhaplotypes.csv
   - flexqtl.par
   - flexqtl.sort

   *From FlexQTL™ run to determine haploblocks:
   - Generated (and adjusted) haploblocks.map

5. Create a new R project in the same folder to run PediHaplotyper

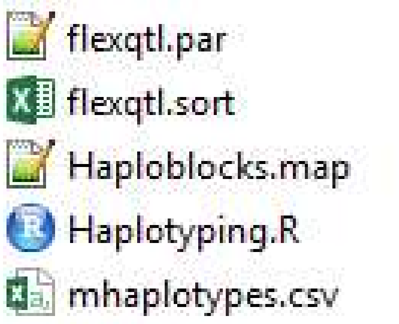

Running PediHaplotyper

6. In RStudio, install PediHaplotyper as a package (only needed once)
   *Tools>Install Packages…
      *Install from: "Package Archive" and Browse to find "PediHaplotyper_1.tar.gz"

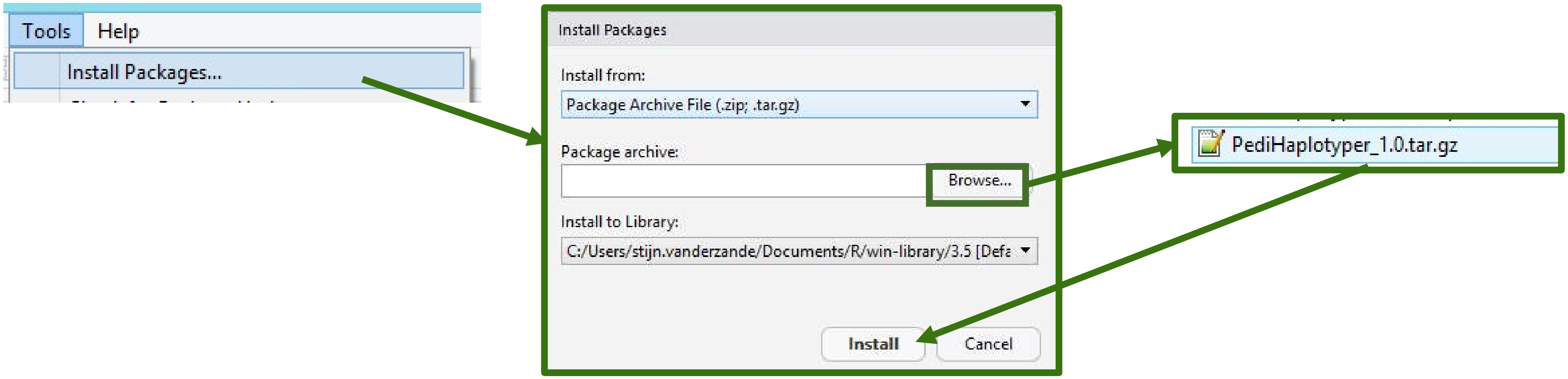

7. Set working directory to "Source File Location"

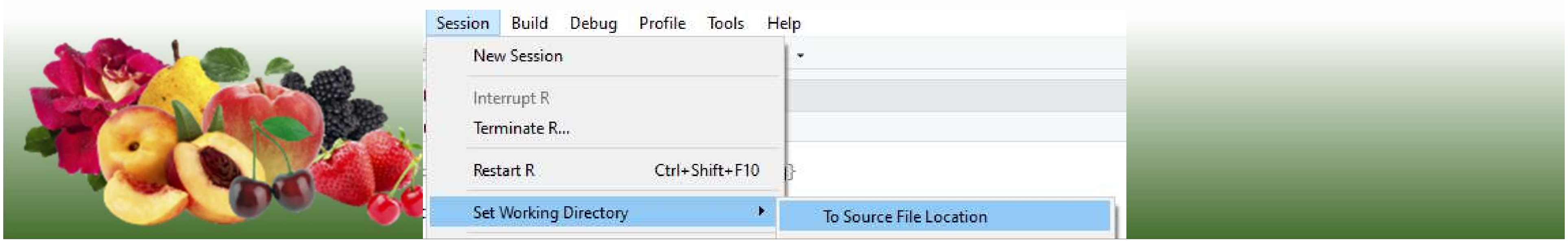

Running PediHaplotyper

8. Run the following code

```
library(PediHaplotyper)

fq_haplotyping_session(sessionID="20180613_HTDet_All", mapfile="Haploblocks.map")
```

Prefix for all output files

Name of haploblock definition file

```
> fq_haplotyping_session(sessionID="20180613_HTDet_All", mapfile="Haploblocks.map")
reading datafiles ...
hb 1 = HB-01-1: 27 initial alleles
hb 2 = HB-01-1a: 3 initial alleles
hb 3 = HB-01-2: 25 initial alleles
- - - - - - - - - - - - - - - - -
writing initial HS family haploblock alleles file ...
calculation haploblock alleles ...
hb 1 = HB-01-1: convergence = yes in 4 cycles
hb 2 = HB-01-1a: convergence = yes in 2 cycles
hb 3 = HB-01-2: convergence = yes in 3 cycles
hb 4 = HB-01-3: convergence = yes in 3 cycles
- - - - - - - - - - - - - - - - -
writing all requested output files ...
haplotyping session 20180613_HTDet_All finished.
>
```

Output files

"orig"-flag vs "final"-flag

-"orig": results after initial haplotype determination

-"final": results add end of haplotype determination (use these files)

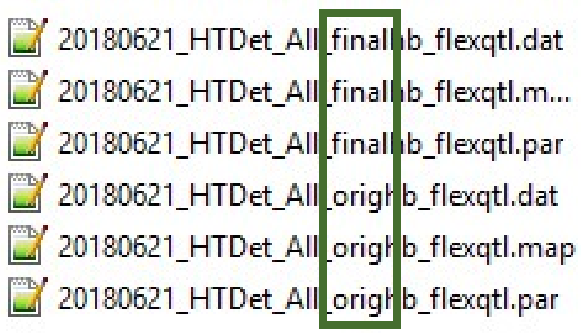

"mrk"-flag vs "hb"-flag

-"mrk": results at the single marker level

-"hb": results at haplotype level (use these files)

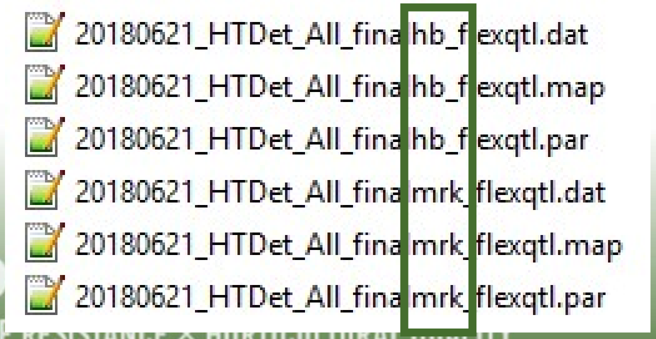

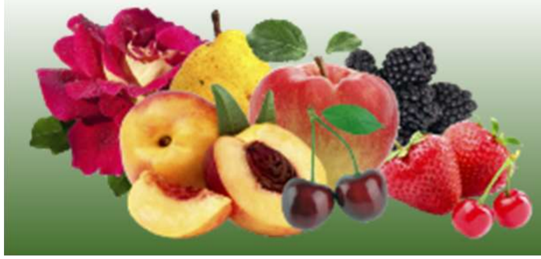

Output files

-All three FlexQTL™ input files (".dat", ".map", and ".par") created for each combination

20180621_HTDet_All_finalhb_flexqt.dat
20180621_HTDet_All_finalhb_flexqt.map
20180621_HTDet_All_finalhb_flexqt.par

-Pedimap input file (".ped") created for each combination

20180621_HTDet_All_finalmrk.ped

- "[…] _hballeles.dat" gives definition of each haplotype for each haploblock

20180621_HTDet_All_hballeles.dat

Haploblock number and name

Number of markers in haploblock

Marker names within haploblock

Marker alleles that create haplotype

hbnr	haploblock	markercount	hballeleID	hballelenr	hacount	freq	4177	1897	1964	7850
1	HB-01-1	4	1	1	4	214	-	-	-	-
1	HB-01-1	4	2	2	1	1	-	A	A	A
1	HB-01-1	4	3	3	1	6	-	B	B	A
1	HB-01-1	4	4	4	0	794	B	A	A	A

Haplotype name and number

Missing values within haplotype

How often haplotype occurs in data set

USDA

United States Department of Agriculture

National Institute of Food and Agriculture

Checking for errors in haplotypes

RosBREED

DISEASE RESISTANCE × HORTICULTURAL QUALITY → SUPERIOR CULTIVARS

Mendelian-inconsistent errors

1. Use "[….]_finalhb" FlexQTL^{TM} input files generated by PediHaplotyper to create a new FlexQTL^{TM} project
   *parameter file should be renamed to "flexqtl.par"
2. Use "mconsistency.csv" file to identify issues
3. Use "[…] _hballeles.dat" file to investigate haplotypes that cause P(P)C errors
   3.a If missing data within haplotype causes inconsistency: assign correct haplotype (if both parental haplotypes are possible, use information on flanking markers and minimize recombinations)

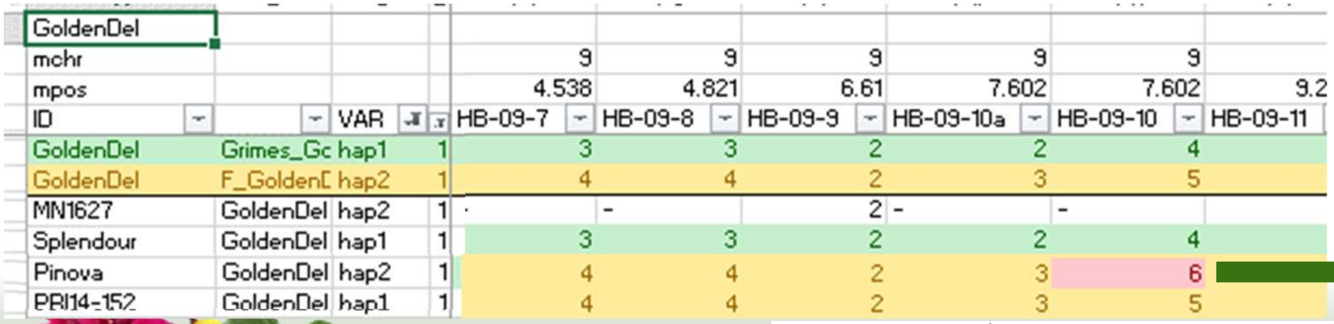

GoldenDel									
mchr				9	9	9	9	9	
mpos				4.538	4.821	6.61	7.602	7.602	9.2
ID		VAR		HB-09-7	HB-09-8	HB-09-9	HB-09-10a	HB-09-10	HB-09-11
GoldenDel	Grimes_Gc	hap1	1	3	3	2	2	4	
GoldenDel	F_GoldenD	hap2	1	4	4	2	3	5	
MN1627	GoldenDel	hap2	1	.	-	2	-	-	
Splendour	GoldenDel	hap1	1	3	3	2	2	4	
Pinova	GoldenDel	hap2	1	4	4	2	3	6	
PRI14-152	GoldenDel	hap1	1	4	4	2	3	5	

Haplotype 6 equals haplotype 5 except for a missing value

HB-09-10	439	6324	3144	6325	3146
4	A	B	A	B	A
5	B	B	A	A	B
6	B	B	A	A	-

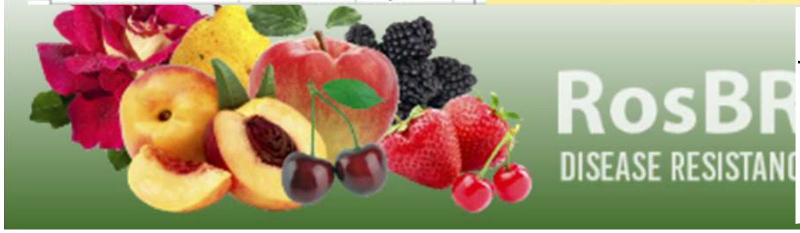

Mendelian-inconsistent errors

3.b If a recombination occurs within haploblock:

- 3.b.1 if recombination occurs within selected material: adjust haploblock borders in “haploblock.map” file and re-determine haplotypes for adjusted haploblocks
- 3.b.2 if recombination occurs in seedling: don’t adjust haploblock borders and do not adjust haplotype (or make it missing)

	A	B	C	D	Y	Z	AA	AB	AC	AD
1	Splendour									
2	mchr				1	1	1	1	1	
3	mpos				22.387	23.021	23.554	24.678	24.89	2
4	ID		VAR		HB-01-19	HB-01-19a	HB-01-20	HB-01-21	HB-01-21a	HB-01-22
65	Splendour	GoldenDel	hap1	1	4	4	4	4	3	
66	Splendour	Delicious	hap2	1	10	7	5	5	2	
3286	Sciros	Splendour	hap2	1	4	4	4	6	2	

HB-01-21	1970	4277	131	2566	6846
4	A	B	A	A	A
5	A	B	B	A	B
6	A	B	A	A	B

Recombination within HB

Mendelian-inconsistent errors

3.c If a genotype calling error occurred (check with GenomeStudio®): adjust haplotype to resemble correct genotype call

3.d If a marker order is incorrect: adjust marker order in “haploblock.map” file, and latest data and map file for single marker level. Re-run FlexQTL™ to create new PediHaplotyper files and rerun pedihaplotyper for affected haploblocks

Mendelian-consistent errors

4. Once Mendelian-inconsistent errors are resolved, check for Mendelian-consistent errors as was done for single marker level

5. Resolve Mendelian-consistent errors as was done for Mendelian-inconsistent errors with haplotypes

6. Enjoy your high-quality genotypic data set and make lots of great discoveries!!
